# Genome-wide identification of cassava *R2R3 MYB* family genes related to abscission zone separation after environmental-stress-induced abscission

**DOI:** 10.1038/srep32006

**Published:** 2016-08-30

**Authors:** Wenbin Liao, Yiling Yang, Yayun Li, Gan Wang, Ming Peng

**Affiliations:** 1 Institute of Tropical Bioscience and Biotechnology, Chinese Academy of Tropical Agricultural Sciences, Haikou 571101, China

## Abstract

Cassava plants (*Manihot esculenta* Crantz) resist environmental stresses by shedding leaves in leaf pulvinus abscission zones (AZs), thus leading to adaptation to new environmental conditions. Little is known about the roles of cassava *R2R3 MYB* factors in regulating AZ separation. Herein, 166 cassava *R2R3 MYB* genes were identified. Evolutionary analysis indicated that the 166 R2R3 MYB genes could be divided into 11 subfamilies. Transcriptome analysis indicated that 26 *R2R3 MYB* genes were expressed in AZs across six time points during both ethylene- and water-deficit stress-induced leaf abscission. Comparative expression profile analysis of similar SOTA (Self Organizing Tree Algorithm) clusters demonstrated that 10 *R2R3 MYB* genes had similar expression patterns at six time points in response to both treatments. GO (Gene Ontology) annotation confirmed that all 10 *R2R3 MYB* genes participated in the responses to stress and ethylene and auxin stimuli. Analysis of the putative 10 *R2R3 MYB* promoter regions showed that those genes primarily contained ethylene- and stress-related cis-elements. The expression profiles of the genes acting downstream of the selected *MYB*s were confirmed to be involved in cassava abscission zone separation. All these results indicated that *R2R3 MYB* plays an important regulatory role in AZ separation.

MYB proteins are involved in many significant physiological and biochemical processes, including hormone synthesis and signal transduction, regulation of ROS (reactive oxygen species), regulation of primary and secondary metabolism, and flavonoid biosynthesis^1^. The MYB gene was first identified in animal cells as an oncogene derived from retroviruses^2^; in the plant kingdom, the MYB transcription factors are also one of the largest and most diverse families of transcription factors. MYBs encode transcription factor proteins that share the conserved MYB DNA-binding domain. MYB proteins are classified into three major groups: *R2R3 MYB*, with two adjacent repeats; *R1R2R3 MYB*, with three adjacent repeats; and a heterogeneous group collectively referred to as the MYB-like proteins, which typically but not always contain a single MYB repeat^3^,^4^.

The *Arabidopsis* genome contains 126 *R2R3 MYB* genes^2^,^3^. Many *R2R3 MYB* genes are involved in regulating environmental stress responses, ROS signaling pathways, and hormone signaling pathways. *AtMYBR1* is suppressed by water stress and wounding, thereby regulating leaf senescence^5^. *GbMYB5* confers drought tolerance in cassava and transgenic tobacco^6^. *MdSIMYB1* from apples is induced by abiotic stresses and plant hormones^7^. *BnaMYB78* from canola (*Brassica napus* L.) is regulated by ABA treatment and abiotic conditions, and modulates reactive oxygen species (ROS)-dependent cell death^8^. *SbMYB15* from the extreme halophyte *Salicornia brachiata* is also induced by various stresses and regulates proline and ROS levels in transgenic plants^9^. Two *R2R3 MYB* genes, *SbMYB2* and *SbMYB7*, from *Scutellaria baicalensis* are induced by different stresses and abscisic acid and enhance resistance to oxidative stress in transgenic tobacco^10^. *OsMYB91* from rice is induced by abiotic stress and increases the levels of proline and the capacity to scavenge active oxygen in transgenic plants^11^. The *R2R3 MYB* protein *SlAN2* from tomatoes elevates ethylene synthesis^12^. *IbMYB1* from sweet potatoes displays increased antioxidant activities in transgenic plants compared with empty vector control plants^13^. *PtsrMYB* from trifoliate oranges is up-regulated by abiotic stresses (dehydration, salt, cold and ABA treatment) and decreases the levels of reactive oxygen species and increases the levels of polyamines^14^. *TaMYB4* from wheat is induced by salicylic acid, ethylene, abscisic acid and methyl jasmonate hormones, and it enhances the response to different stresses and promotes disease resistance^15^. *OsMyb4* from rice is induced by osmotic stress and enhances the cellular antioxidant capacity^16^. Four R2R3-type MYB transcription factors, including *MYB3, MYB4*, *MYB7* and *MYB32*, are induced by ethylene and regulate the ROS pathway^17^. *AtMYB15* from *Arabidopsis* is induced by ABA, drought and salt treatments and enhances the sensitivity to abscisic acid and improves drought tolerance^18^. *TaMYB73* from wheat is induced by heat stress and regulates antioxidant enzyme activity^19^. *SnMYB73* from tomatoes is induced by abiotic stress and regulates auxin signaling pathways^20^. *AtMYB62* from *Arabidopsis* is induced by environmental stress and regulates GA metabolism and signaling^21^. *AtMYB43* from *Arabidopsis* is induced by stresses and regulates secondary cell wall biosynthesis in *Arabidopsis thaliana*^22^. *MYB78* from sorghum is induced by abiotic stresses^23^. *AtMyb96* serves as a molecular link that mediates ABA-auxin cross talk during the drought-stress response as well as during lateral root growth^24^.

Cassava (*Manihot esculenta* Crantz) is one of the most important starch-producing plants in the world. Cassava plants have clear abscission zone structures in their leaf pulvinus-petioles, which confer resistance to environmental stresses^25^. The separation of abscission zones in cassava can be induced by numerous different stresses, including water-deficit stress and ethylene treatment. In our previous work, by using transcriptomic, physiological, cellular, molecular, metabolic and transgenic methods, we have found that ROS and ethylene regulate the separation of abscission zones under water-deficit stress. Moreover, ROS are increased by the accumulation of proline and polyamine in cassava abscission zones under water-deficit stress^25^. Under various environmental stresses, numerous stress-related *R2R3 MYB* genes have been identified in *Arabidopsis*^26^, cotton^3^, rice^4^,^27^, soybeans^28^, *Populus*^29^ and maize^30^. However, no studies have identified and characterized cassava abscission zone *R2R3 MYB* super-family members.

Here, we surveyed *R2R3 MYB* family members by using phylogenetic analysis of the 166 cassava *R2R3 MYB* genes and found that the number of members was approximately 1.32-fold higher than that in the *Arabidopsis* genome. Evolutionary analysis indicated that 166 cassava R2R3 MYB genes could be divided into 11 subfamilies. The amino acid motifs and phylogenetic tree were predicted and analyzed. Transcriptome analysis was used to identify the *R2R3 MYB* genes expressed in cassava leaf pulvinus-petiole abscission zones by comparing the *R2R3 MYB* gene expression profiles of ethylene- and water-deficit stress-induced leaf abscission. In total, 41 and 38 *R2R3 MYB* genes were expressed during ethylene- and water-deficit stress-induced leaf abscission, respectively. In total, 26 *R2R3 MYB* genes were identified as being expressed in AZs across six time points during both ethylene- and water-deficit stress-induced leaf abscissions. Comparative expression profile analysis of similar SOTA (Self Organizing Tree Algorithm) clusters at six time points during ethylene- and water-deficit stress-induced leaf abscission demonstrated that 10 *R2R3 MYB* subfamily genes exhibited similar expression patterns in response to both treatments. GO (Gene Ontology) annotation confirmed that all 10 *R2R3 MYB* subfamily genes participate in the pathways associated with responses to stress and ethylene and auxin stimuli. Analysis of the putative 10 *R2R3 MYB* promoter regions revealed that the genes primarily contained ethylene- and stress-related cis-elements. Finally, the expression ratios of 9 *R2R3 MYB* subfamily genes were confirmed by qPCR. Our results should be useful for determining the precise role of *R2R3 MYB* genes participating in the process of cassava abscission zone separation.

## Results

### Cassava *R2R3 MYB* gene identification

To identify MYB-related proteins in the cassava genome, BLASTP searches of the complete genomes in *Manihot esculenta* (annotation v.6.1) were performed by using *Arabidopsis* MYB protein sequences as queries^3^. A total of 216 putative amino acid sequences in the cassava genome containing MYB or MYB-like repeats were identified form the cassava genome database. Subsequently, the redundant sequences of candidate MYBs were discarded from the data set according to the similarity of the sequences. A simple modular architecture research tool was used to analyze all the putative MYBs to confirm the MYB domain^3^. The remaining cassava MYBs possessing incomplete ORFs were also excluded from further analysis. All cassava MYB proteins were manually inspected to ensure that the putative genes contained 2 or 3 MYB repeats. In total, 166 genes were defined as *R2R3 MYB* proteins in the cassava genome (Additional file 1). The gene set represented approximately (166/33,033) 0.5025% of the annotated genes in the cassava genome (33,033 genes), a proportion greater than that of *Arabidopsis* genes (0.4596%).

**Figure 1:**
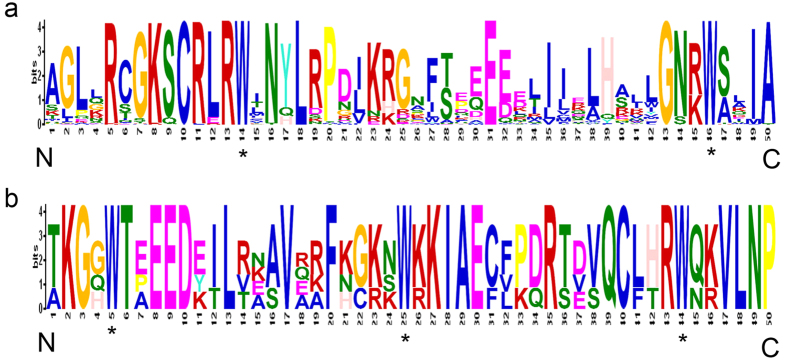
The R2 and R3 MYB repeats are highly conserved across 166 *R2R3 MYB* proteins in the cassava genome. The sequence logos of the R2 (**a**) and R3 (**b**) MYB repeats are based on full-length alignments of 166 *R2R3 MYB* proteins in the cassava genome. The bit score indicates the information content for each position in the sequence. Asterisks indicate the conserved tryptophan residues (Trp) in the MYB domain.

To confirm that the R2 and R3 MYB repeats are highly conserved across all 166 *R2R3 MYB* proteins identified form the cassava genome, multiple alignment analysis was conducted to identify the homologous *R2R3 MYB* domain in all 166 MYB proteins by investigating the frequency of the most prevalent amino acids within the MYB domain^3^. In general, the basic regions of the R2 and R3 repeats both had 50 basic residues (Fig. 1). The R2 and R3 repeats of cassava were highly conserved in sequences; 10 out of 50 in R2 and 24 out of 50 amino-acids in R3 were 100% conserved in all MeMYB proteins (Fig. 1). As compared with those in other plant species, the R2 and R3 MYB repeats in cassava *R2R3 MYB* family contain characteristic amino acids. For example, highly conserved Trp residues were distributed in 166 cassava R2R3 MYB that have been demonstrated to play a role in the sequence-specific binding domain^3^ (Fig. 1).

### Phylogenetic reconstruction of the *R2R3 MYB* superfamily between cassava and *Arabidopsis*

**Figure 2:**
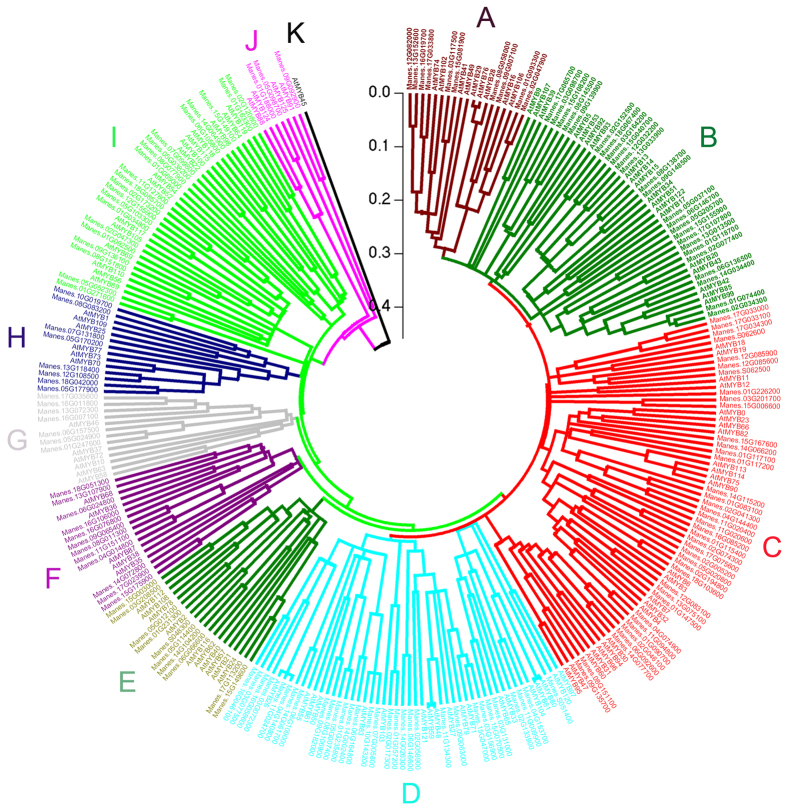
Phylogenetic tree of all *R2R3 MYB* transcription factors from cassava and *Arabidopsis*. The sequences of *R2R3 MYB* transcription factors from cassava (166) and *Arabidopsis* (126) were aligned using ClustalW, and the phylogenetic tree was constructed with MEGA 5.0 using the neighbor-joining method based on the p-distance model with 1000 bootstrap replicates. Each subfamily is represented by a specific color.

To study the phylogenetic relationships among the cassava *R2R3 MYB* super-family members in different plant species, all identified cassava *R2R3 MYB*s and all *R2R3 MYB*s from *Arabidopsis* were subjected to multiple sequence alignment^3^, and a phylogenetic tree was generated by using the *R2R3 MYB* amino acid sequences from cassava and *Arabidopsis*. The resulting phylogenetic tree contained 11 groups, termed groups A to K (Fig. 2). Most of the groups contained *R2R3 MYB* from both the cassava and the *Arabidopsis* genomes, and all these members from both *Arabidopsis* and cassava were grouped together, thus indicating that these genes may have the same function^3^. The C group had the most members, including 64 members from both cassava and the *Arabidopsis* genome. Specifically, 40 members were identified form cassava, and 24 were identified from *Arabidopsis*. The K group contained the least number of members. This group included only 1 member from the *Arabidopsis* genome. The D and J groups contained sister gene pairs identified in both *Arabidopsis* and cassava on the basis of the phylogenetic tree. The two sister gene pairs, AtMYB121 and Manes.02G058900 and AtMYB103 and Manes.02G017300 were grouped in the D group, whereas AtMYB91 and Manes.09G092900 were grouped in the J group.

### Phylogenetic analysis and analysis of conserved gene structures and protein motifs of the *R2R3 MYB* gene family in cassavas

**Figure 3:**
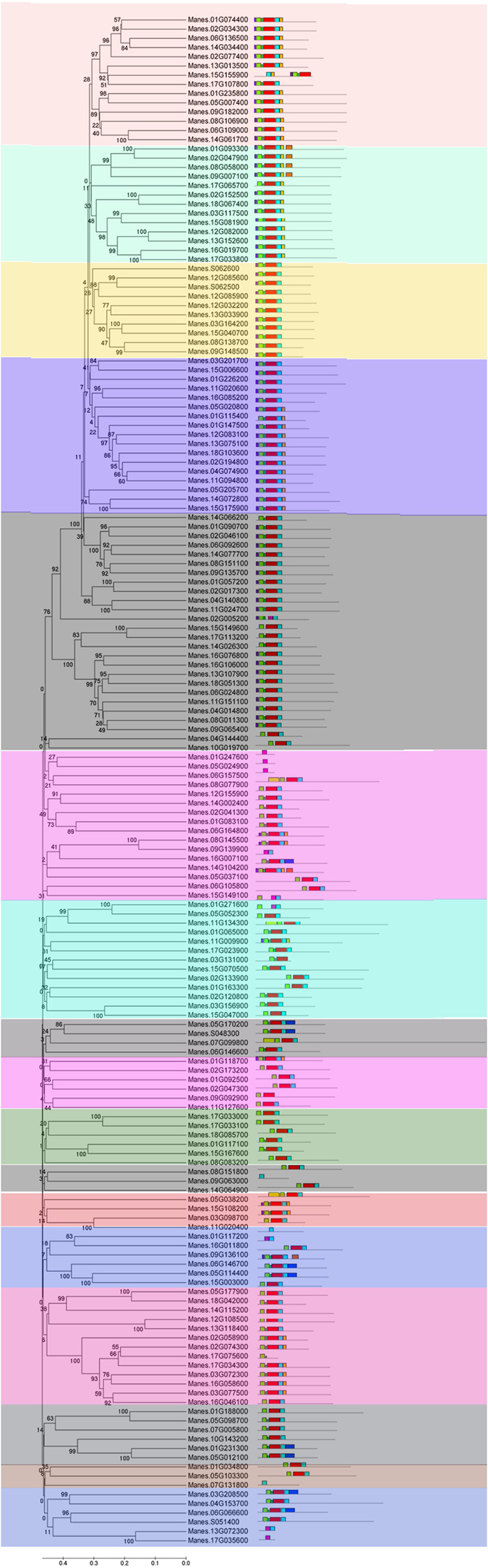
Phylogenetic relationships and motif compositions of 166 *R2R3 MYB* proteins in the cassava genome. The phylogenetic tree was constructed with MEGA 5.0 using the neighbor-joining (NJ) method with 1,000 bootstrap replicates based on a multiple alignment of 166 amino acid sequences of *R2R3 MYB* genes from cassava. Bootstrap values greater than 50% are presented on the nodes. The 17 major subfamilies are indicated, with S1 to S17 marked with colorful backgrounds. Protein motif schematic diagram of the conserved motifs in the *R2R3 MYB* proteins in cassava, which were elucidated using MEME. Each motif is represented by a number in the colored box. The black lines represent the non-conserved sequences.

To study the phylogenetic relationships among the cassava *R2R3 MYB* superfamily members, a phylogenetic tree was generated with 166 *R2R3 MYB* amino acid sequences from cassava (Fig. 3). On the basis of the clades with at least 50% bootstrap support, the 166 typical members of the cassava *R2R3 MYB* gene family were subdivided into 17 subgroups and designated as S1 to S17 (Fig. 3). Moreover, the tree topology after maximum likelihood (ML) analysis was essentially the same as that of the former unrooted phylogenetic tree^3^, thus indicating that these phylogenetic trees were in good agreement. The low bootstrap support for the internal nodes of these trees was consistent with phylogenetic analysis of MYBs in other organisms. A total of 62 sister pairs of putative paralogous genes were identified among the 166 *R2R3 MYB* genes, and 26 of them had high bootstrap support (≥98%).

In order to identify the conserved motifs in the MYB protein sequences, the Multiple Em for Motif Elicitation (MEME; version 4.9.0) program tool was used to analyze all the full-length protein sequences of 166 MeMYBs. Ten motifs were identified in the MeMYBs, and the motif lengths identified by MEME were between 8 and 50 amino acids (Fig. 3). The number of the conserved motifs in each MYB varied between 1 and 9. Most MeMYBs had motifs 1, 2, 3, and 6, and most members in the same subgroup shared more than one identical motif. The majority of the close members in the phylogenetic tree exhibited similar motif compositions, thus suggesting that the members may have the similar function in the same subgroup^3^.

### Transcriptome identification of *R2R3 MYB* genes expressed in cassava leaf pulvinus-petiole AZs during both ethylene- and water-deficit stress-induced leaf abscissions

**Figure 4:**
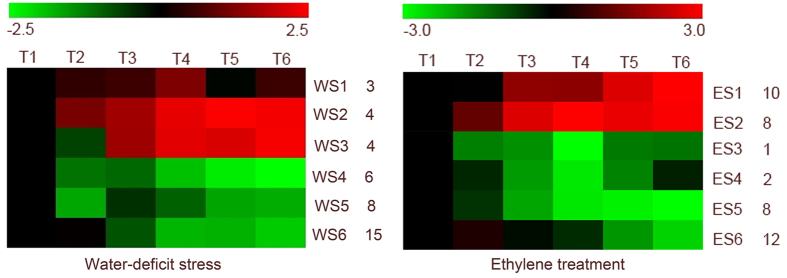
SOTA clustering showing the expression profiles of ethylene- and water-deficit stress-induced leaf abscission. SOTA clustering identified six *R2R3 MYB* gene expression clusters among the six leaf abscission time points (41 and 38 *R2R3 MYB* genes for ethylene- and water-deficit stress-induced leaf abscission, respectively). The signals are indicated using a red-green color scale, where red and green represent increased and reduced expression, respectively.

To analyze the *R2R3 MYB* gene expression profiles during ethylene and water-deficit stress-induced leaf abscission, cassava genome microarray (NimbleGen) analyses were performed. The results indicated that 41 *R2R3 MYB* genes were differentially expressed after ethylene-induced leaf abscission, and 38 *R2R3 MYB* genes were differentially expressed after water-deficit stress-induced leaf abscission (Additional table 2). In total, 26 *R2R3 MYB* genes were differentially expressed after both ethylene- and water-deficit stress-induced leaf abscissions. In contrast, 15 *R2R3 MYB* genes were differentially expressed exclusively after ethylene-induced leaf abscission, and 12 were differentially expressed after water-deficit stress-induced leaf abscission (Additional table 2). Of the *R2R3 MYB* genes induced by the water-deficit stress treatment, six SOTA clusters (WS1-WS6) were separated into four groups of primary expression patterns (Fig. 4 and Additional table 3). The first group, cluster WS1, was up-regulated at the early and middle stages during abscission compared with the control, whereas the second group, clusters WS2 and WS3, was up-regulated at the later experimental time points. The third group, clusters WS4 and WS6, was down-regulated at the later experimental time points T4, T5 and T6. The fourth group, cluster WS5, was down-regulated at the early experimental time points (Fig. 4 and Additional table 3).

The six ethylene-induced *R2R3 MYB* gene SOTA clusters (ES1-ES6) were divided into four main expression patterns (Fig. 4 and additional table 4). The first group, cluster ES1, was up-regulated at the later experimental time points. The second group, cluster ES2, was up-regulated throughout the abscission process, with the highest levels of expression at the early and middle experimental points. The third group, clusters ES3 and ES4, was down-regulated later at T4, T5 and T6. The fourth group, clusters ES5 and ES6, was down-regulated at the early and middle experimental time points compared with the control.

### Comparison of *R2R3 MYB* expression profiles between ethylene- and water-deficit stress-induced leaf abscissions indicated that *R2R3 MYB* subfamily genes are widely expressed in the cassava abscission zone

To identify the *R2R3 MYB* genes that participated in both ethylene- and water-deficit stress-induced leaf abscissions, the *R2R3 MYB* expression profiles were compared in both treatments by using SOTA clustering. Because the expression patterns of *R2R3 MYB* genes were nearly identical between ethylene- and water-deficit stress-induced leaf abscissions, the similar expression patterns at each time point were compared in both ethylene and water-deficit stress treatments.

**Figure 5:**
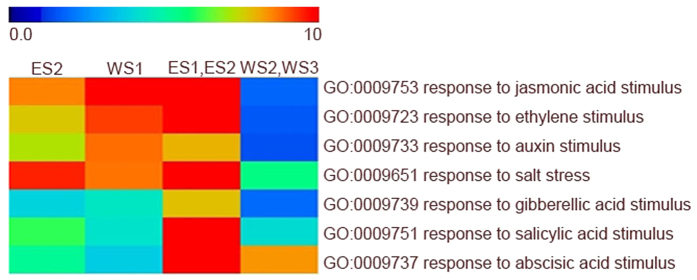
GO functional category enrichment among the clusters in both ethylene- and water-deficit stress-induced leaf abscission.

The *R2R3 MYB* genes that were up-regulated during the early and middle experimental periods in response to both treatments (Fig. 4) were first examined. During water-deficit stress treatment-induced leaf abscission, 3 *R2R3 MYB* genes in the WS1 cluster exhibited increased expression during the early stages of water-deficit stress-induced leaf abscission, and GO annotations indicated that all 3 genes participate in the responses to an ethylene stimulus (GO:0009723) and an auxin stimulus (GO:0009733) (Fig. 5). During ethylene treatment-induced leaf abscission, 8 *R2R3 MYB* genes in the ES2 cluster exhibited increased expression during the early and middle stages of leaf abscission. GO annotation indicated that 5 of the genes participate in the response to stress (GO: 0009651), 4 genes participate in the response to an ethylene stimulus (GO:0009723), and 4 genes participate in the response to an auxin stimulus (GO:0009733) (Fig. 5). Two genes, Manes.15G040700 and Manes.05G177900, were expressed during the early and middle stages during leaf abscission in response to both ethylene and water-deficit stress, and both participate in the pathways in response to an ethylene stimulus (GO:0009723) and to an auxin stimulus (GO:0009733). Manes.15G040700 encodes MYB15, a member of the R2R3 factor gene family. Manes.15G040700 exhibits a high expression ratio (compared with the T1 time point) at the T4 time point in both ethylene and water-deficit stress treatments. Manes.05G177900 encodes MYB73, a member of the R2R3 factor gene family. A high expression ratio (compared with the T1 time point) appeared at the T4 time point in ethylene treatment and the T3 time point in water-deficit stress.

Later in leaf abscission (T4, T5 and T6), WS2, WS3 (water-deficit stress treatment) and ES1, ES2 (ethylene treatment) exhibited similar expression patterns (Fig. 4). In response to water-deficit treatment, 6 *R2R3 MYB* genes exhibited increased expression. GO annotation indicated that 4 participate in response to an abscisic acid stimulus (GO:0009737), and 3 participate in response to stress (GO:0009651) (Fig. 5). In response to ethylene treatment, 17 *R2R3 MYB* genes exhibited increased expression. GO annotation indicated that 10 of the genes participate in response to stress (GO:0009651), 8 genes participate in response to an ethylene stimulus (GO:0009723), and 5 of genes participate in response to an auxin stimulus (GO:0009733) (Fig. 5). Comparative analysis indicated that 8 genes, i.e., Manes.05G114400, Manes.01G118700, Manes.15G081900, Manes.02G194800, Manes.06G066600, Manes.05G012100, Manes.14G104200, and Manes.03G117500, were expressed during both ethylene- and water-deficit stress-induced leaf abscission. Manes.05G114400 encodes MYB62, a member of the *R2R3 MYB* transcription family. Manes.05G114400 exhibited a high expression ratio (compared with the T1 time point) at the T6 time point in both ethylene and water-deficit stress treatments. Manes.01G118700 encodes MYB43, and a high expression ratio (compared with the T1 time point) appeared at the T5 time point in water-deficit stress treatment. However, this gene was down-regulated in ethylene-induced abscission. Manes.15G081900 encodes MYB4, a member of the *R2R3 MYB* transcription family, which is involved in wounding and the osmotic stress response. This gene exhibited a high expression ratio (compared with the T1 time point) at the T6 time point in both ethylene and water-deficit stress treatments. Manes.02G194800 encodes MYB3, and a high expression ratio (compared with the T1 time point) appeared at the T6 time point in both ethylene and water-deficit stress treatments. Manes.06G066600, which encodes MYB62, a member of the *R2R3 MYB* transcription family involved in regulation of phosphate starvation responses, exhibited a high expression ratio (compared with the T1 time point) at the T6 time point in both ethylene and water-deficit stress treatments. Manes.05G012100 encodes MYB78, a member of the *R2R3 MYB* transcription family. This gene exhibited a high expression ratio (compared with the T1 time point) at the T5 time point in ethylene treatment and the T6 time point in water-deficit stress treatment. Manes.4G104200 encodes MYB62, which had a high expression ratio (compared with the T1 time point) at the T6 time point in both ethylene and water-deficit stress treatments. Manes.03G117500 encodes MYB4, which exhibited a high expression ratio (compared with the T1 time point) at the T4 time point in both ethylene and water-deficit stress treatments.

### Promoter motif prediction for *R2R3 MYB* subfamily genes expressed during both ethylene- and water-deficit stress-induced leaf abscission

As described above, 10 genes were expressed in response to both ethylene and water-deficit stress treatments. To further understand the potential functions of the genes in regulating abscission zone development, the promoters of the 10 *R2R3 MYB* subfamily genes were analyzed to identify cis-elements. For this analysis, 2-kbp sequences of putative promoter regions were examined for potential cis-regulatory elements that are responsive to water-deficit stress and ethylene. Three drought stress response cis-elements, S000415, S000414 and S000176, as well as two ethylene response cis-elements, S000037 and S000457, were frequently identified within the promoter regions of the genes. The results suggested that all the genes contained more than 20 drought stress elements and more than 5 ethylene response elements (Additional table 5).

### Quantitative real-time RT-PCR analysis of expression profiles for 10 selected *R2R3 MYB* genes in cassava leaf pulvinus-petiole AZs during both ethylene- and water-deficit stress-induced leaf abscission

To confirm the reliability of the 10 selected *R2R3 MYB* genes that were up-regulated in the transcriptome data, FDR-corrected P-values < 0.005 were used as the significance criterion in at least one of the time points in both ethylene- and water-deficit stress-induced leaf abscission, to evaluate the expression patterns in transcriptome. As shown in Table 1, most of the FDR-corrected P-values of 10 selected *R2R3 MYB* genes in the transcriptome that were expressed at six time points in both ethylene and water-deficit stress treatments were less than 0.005. Moreover, some of the FDR-corrected P-values in at least one of the time points in both treatments were less than 1e-6. Compared with the FDR-corrected P-values in water-deficit stress-induced abscission, more values of the FDR-corrected P-values less than 1e-6 appeared in ethylene stress-induced abscission. Together, the data suggested that the 10 selected *R2R3 MYB* genes that were up-regulated in the transcriptome in both ethylene- and water-deficit stress-induced leaf abscissions were reliable.

**Figure 6:**
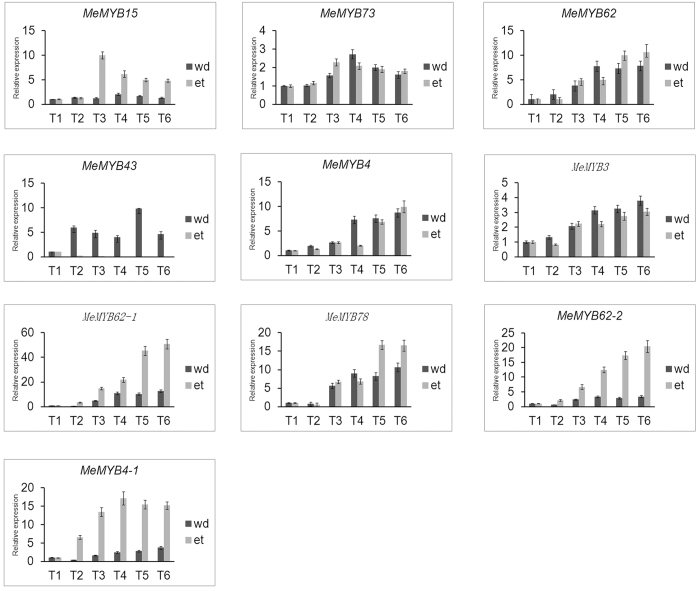
Expression profiles of 10 *MeMYB* genes in both ethylene- and water-deficit stress-induced leaf abscission.

To further confirm the expression patterns of 10 *R2R3 MYB* subfamily genes in leaf abscissions, their expression levels were identified by quantitative real-time RT-PCR. The relative expression of 10 *R2R3 MYB* genes was significantly different in both ethylene- and water-deficit stress-induced leaf abscissions (Fig. 6). Most of the selected genes had a similar expression pattern in both treatments, except for *MeMYB43*. For example, 9 of 10 selected genes exhibited up-regulated expression at the later stages of abscission in both treatments, whereas *MeMYB43* was up-regulated in response to water-deficit stress and down-regulated in response to the ethylene treatment. *MeMYB15*, *MeMYB43*, *MeMYB62*, *MeMYB62-1*, *MeMYB78*, *MeMYB62-2*, and *MeMYB4-1* were more up-regulated in ethylene-induced abscissions than in water-deficit stress-induced abscissions. The maximum expression ratio (nearly 60-fold) was observed for *MeMYB62-1* in the ethylene-induced abscission. *MeMYB73*, *MeMYB4* and *MeMYB3* were more up-regulated in water-deficit stress-induced abscissions than in ethylene-induced abscissions.

## Discussion

### Comparative analysis of cassava and *Arabidopsis* indicated more *R2R3 MYB* subfamily members in the cassava genome than in the *Arabidopsis* genome

Numerous *R2R3 MYB* genes have been identified in various plant species as more plant genomes are sequenced. The number of *R2R3 MYB* genes varies among the species. For example, 126 *R2R3 MYB*s have been identified in *Arabidopsis*, 192 in *Populus trichocarpa*, 244 in Soybean, 108 in *Vitis vinifera*, 88 in *Oryza sativa*, 205 in cotton and 157 in maize^3^. The study is the first to identify and characterize the *R2R3 MYB* genes in cassava. Herein, 166 cassava *R2R3 MYB* genes were classified into 11 subfamilies (Additional file 1 and Fig. 2). The number of cassava *R2R3 MYB* subfamily members (166) far exceeded that in the *Arabidopsis* genome (126), thus indicating that the abundance of *R2R3 MYB* genes in cassava have expanded potentially via gene duplications. Gene duplication is considered to be the primary driving force underlying new gene functions^25^,^31^. In this study, numerous *R2R3 MYB* genes were observed to participate in both ethylene- and water-deficit stress-induced abscissions, thus suggesting that the *R2R3 MYB* subfamily members have important functions in cassava plant development.

### *R2R3 MYB* subfamily genes suggested the function of regulating the progression of cassava leaf abscission by participating in various biological pathways

In our previous work, we have reported that ROS regulate the separation of abscission zones under water-deficit stress through transcriptomic, physiological, cellular, molecular, metabolic, and transgenic methods. Moreover, ROS are increased by the accumulation of proline and polyamine in cassava abscission zones under water-deficit stress^25^. The levels of proline, polyamine, ROS, ethylene and auxin have been proven to have the function of regulating the process of abscission zone development^25^. Here, molecular methods were used to identify the key *R2R3 MYB* genes that regulate cassava AZ separation in response to ethylene and water-deficit stress. To identify the *R2R3 MYB* genes expressed during cassava leaf abscission in response to both treatments, the *R2R3 MYB* gene expression profiles of pulvinus-petiole AZs were used for SOTA clustering. In total, 41 and 38 *R2R3 MYB* genes were expressed during ethylene- and water-deficit stress-induced leaf abscission, respectively (Additional table 2). Among the *R2R3 MYB* genes, 26 were expressed during both ethylene- and water-deficit stress-induced leaf abscission (Additional table 2), thus suggesting that similar genes regulate leaf abscission in response to these stresses. Comparative analysis of *R2R3 MYB* gene expression profiles during ethylene- and water-deficit stress-induced leaf abscission resulted in the selection of 9 *R2R3 MYB* genes distributed among the different time points, thus suggesting that *R2R3 MYB* subfamily genes play important roles in cassava abscission zone development.

During early and middle leaf abscission, high levels of *MeMYB15* and *MeMYB73* expression were detected (Fig. 4). *MYB15* decreases oxidative damage and increases proline in transgenic plants under salinity and dehydration conditions^9^,^18^. Under the stresses of salinity, dehydration and heat, many stress-responsive genes, such as *LEA5*, *ERD10D*, *PLC3*, *LTP1*, *HSF2*, *ADC*, *P5CS*, *SOD* and *CAT*, have been reported to act down stream of *MYB15*^9^,^18^. Proline and ROS regulate the development of cassava abscission^25^, and *ADC* and *P5CS* have been reported to up-regulate during the separation of cassava abscission zones^25^. Ethylene signaling plays essential roles in mediating plant responses to biotic and abiotic stresses. *MYB73* also regulates antioxidant enzyme activity^24^. In addition, wheat *Myb73* overexpression leads to the activation of the ABA-independent pathway genes *RAB18* and *CBF3*, as well as the ABA-dependent genes *ABF3* and *RD29B*, thereby improving salinity stress tolerance in *Arabidopsis*^24^. In addition, *AtMYB15* expression is increased in response to the constitutive expression of *ACO1* in *Arabidopsis*^18^, thus suggesting that ethylene signaling can regulate the function of *MYB15.* In addition, promoter motif analysis has also confirmed that *MYB15* responds to the ethylene pathway^32^. All these results suggest that *MeMYB15* regulates the separation of cassava abscission zones. High levels of *MeMYB15* and *MeMYB73* expression were detected at the early and middle leaf abscissions, thus suggesting that *MYB* genes participate in the regulation of abscission zone separation through ROS pathways and proline production.

Later in abscission, 7 *R2R3 MYB* genes, including 1 *MeMYB78*, 1 *MeMYB3*, 2 *MeMYB4* and 3 *MeMYB62* genes, were highly expressed between T2 and T6 in response to both ethylene and water-deficit stress treatment (Fig. 4). The genes that exhibited this expression pattern primarily contributed to cassava leaf abscission. *MYB78* regulates reactive oxygen species (ROS)-dependent cell death^8^ in transgenic *MYB78* plants. The expression of many ROS-related genes is up-regulated. For example, *RbohB* is up-regulated in response to H_2_O_2_ accumulation, exhibiting approximately 6-fold increased expression in transgenic plants compared with controls. *APX*, *CAT3* and *GST* are most prominently increased, with an average of 3- to greater than 10-fold increased expression in transgenic plants compared with controls^8^,^23^. In our previous work, we have found that the ROS levels decrease after co-overexpression of the ROS-scavenging proteins SOD and CAT1 in cassava. ROS also regulates cassava abscission zone separation^25^. In addition, *MeMYB78* regulates ROS-dependent cell death, thus suggesting that it participates in the process of PCD in abscission zone. *MeMYB3* regulates ROS via antioxidant biosynthesis pathways^33^. *MYB3* also contains a conserved MYB domain and an ethylene responsive element binding factor-associated amphiphilic repression (EAR) repression domain^17^,^33^, thus suggesting that this gene is involved in the response to ethylene. *MYB4* has been reported to respond to salicylic acid, ethylene, abscisic acid and methyl jasmonate hormones and also to enhance cellular antioxidant capacity through radical scavenging mechanisms and increased activities of phenylpropanoid and isoprenoid metabolic processes involving various abscisic acid (ABA), jasmonic acid (JA), salicylic acid (SA), ethylene and reactive oxygen species (ROS) responsive genes^15^,^16^. MYB62 regulates responses to environmental stresses via changes in GA metabolism and signaling^21^. GA metabolism and signaling have been reported to induce programmed cell death (PCD) in wheat aleurone cells^34^. The expression of ASYMMETRIC LEAVES1 (AS1), a R2R3 MYB transcription factor from *Arabidopsis*, is also regulated by the gibberellin (GA) pathway. AS1 regulates abscission zone placement in *Arabidopsis* flowers via restricting expression of the *KNOX* genes and *BREVIPEDICELLUS (BP*) in the sepals. Moreover, abscission of the medial sepals is delayed in as1 flowers^35^,^36^. Cassava MYB62 and *Arabidopsis* AS1 have been found to have high sequence similarity through blast analysis. *KNOX* genes regulate organ abscission via the IDA peptide signaling pathway through the HAESA (HAE) and HAESA-LIKE2 receptor-like kinases^35^,^36^. We have also confirmed that GA regulate cassava abscission zone separation^25^. *MeMYB62* potentially participates in regulating cassava abscission zone separation.

### The expression profiles of the genes acting downstream of the selected *MYB*s indicate involvement in cassava abscission zone separation

**Figure 7:**
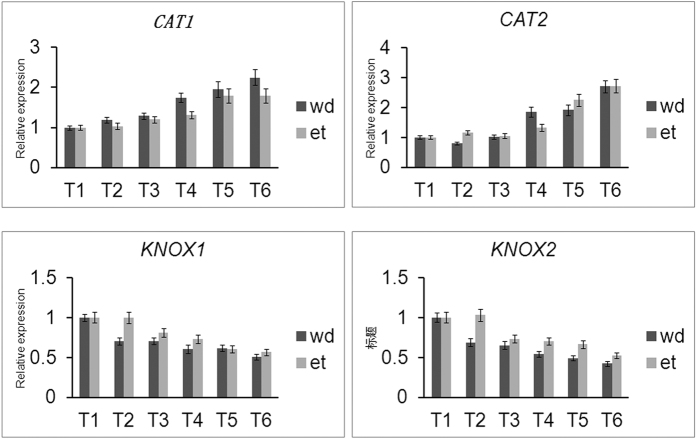
Identification of the expression profile of *CAT*s and *KNOX*s involved in cassava abscission zone development and acting downstream of the selected *MYB*s. wd: water-deficit stress; et: ethylene.

The above analyses identified many genes acting downstream of the selected *MYB*s; therefore, we analyzed the involvement of the putative downstream genes in the process of cassava AZ. *CATs* acted downstream of both MYB78 and MYB15, which regulate reactive oxygen species (ROS)-dependent cell death in AZ separation^8^,^25^. Two *CAT* genes were confirmed to express in abscissions in both ethylene and water-deficit stress treatments (Fig. 7). These two genes were expressed at high levels at late stages in both ethylene- and water-deficit stress-induced abscission. The highest expression ratio of *CAT1* and *CAT2* appeared at the T6 time point at 2.3-fold and 2.7-fold increased expression, respectively, compared with the T1 time point (Fig. 7). *KNOX*s act downstream of R2R3 *MYB*^35^,^36^, and negatively regulate the development of AZs. Two *KNOX*s were confirmed to be downregulated in abscissions in both ethylene and water-deficit stress treatments (Fig. 7). The two genes were expressed at low levels at late stages in both ethylene- and water-deficit stress- induced abscissions. The lowest expression ratio of *KNOX1* and *KNOX2* appeared at the T6 time point at 0.42-fold and 0.5–fold reduced expression, respectively, compared with the T1 time point (Fig. 7). *ADC*, *P5CS* and *ACO1* act downstream of *MYB73* and *MYB15*^9^,^18^ and regulate cassava AZ abscission, as noted in our previous work^25^. *MYB3* and *MYB4* respond to ethylene, and ethylene has also been confirmed to increase in the process of abscission in cassava AZs^25^. All these genes that regulate AZ separation acted downstream of the selected *MYB*s and exhibited distinct expression in both ethylene- and water-deficit stress-induced abscissions in cassava. These results indicated that the selected *MYB*s regulate cassava AZ separation via these functional genes.

In conclusion, this study sought to identify the *R2R3 MYB* genes that regulate cassava abscission zone development. More *R2R3 MYB* subfamilies were identified in the cassava genome than in the *Arabidopsis* genome. Comparative analysis of transcriptome expression profiles in response to ethylene and water-deficit stress indicated that most *R2R3 MYB* genes were expressed in response to both treatments. These results suggested that the same *R2R3 MYB* genes may function in leaf abscission in response to different treatments. Comparisons of *R2R3 MYB* genes expressed at the same time points during abscission in response to both treatments indicated that 9 *R2R3 MYB* subfamily genes were highly expressed during leaf abscission. Further analysis of promoter cis-elements confirmed that the *R2R3 MYB* subfamily responds to ethylene and regulates cassava abscission zone development. These data should provide a foundation for future research on the functional characterization of *R2R3 MYB* genes and signal transduction pathways.

## Materials and Methods

### Identification of cassava *R2R3 MYB* family genes

Cassava genome sequences suggested to contain a MYB domain were isolated and identified as candidate *R2R3 MYB* genes by using the *Arabidopsis* genome as a refs 3,37. *R2R3 MYB* genes were isolated from the cassava genome (JGI database, version 6.1) using annotations and BLAST analysis with an E-value cutoff set as 0.00001^3^. The *Arabidopsis R2R3 MYB* genes were identified using the *Arabidopsis* Information Resource (TAIR)^3^,^37^. Each putative cassava *R2R3 MYB* gene was searched against the TAIR database by using BLAST to ensure that no additional related genes were selected^3^,^37^.

### Phylogenetic analysis

The phylogenetic tree was constructed using neighbor-joining methods. Phylogenetic analysis was performed using MEGA software, version 5. The resulting tree was tested for reliability using bootstrapping with 1,000 replicates and amino acid p-distance parameters^3^,^38^.

### Gene motif detection in cassava

In order to investigate the conserved motifs other than MYB repeats in the MYB protein sequences, we used the program of Multiple Em for Motif Elicitation (MEME; version 4.9.0) tool to analyze all of the full-length protein sequences of 166 MeMYBs^3^. The following parameter settings were used: distribution of motifs, zero or one per sequence; maximum number of motifs to find, 10; minimum width of motif, 6; maximum width of motif, 300 (to identify long R2R3 domains). Other options used the default values. Only motifs with an e-value of <1e-20 were retained for further analysis^3^.

### Plant materials and treatments

*SC5* plants were grown as previously described^25^,^30^. In detail, cassava plants were planted in plastic pots at 28 °C under a 16-h light photoperiod (130 μmol·m^−2^·s^−1^) for 3 months in a greenhouse^25^. Three plants were planted in one pot, and three pots served as a biological replicate. Three-month-old cassava plants with uniform growth statuses were chosen for ethylene and water-deficit stress treatments. Water-deficit stress and ethylene treatments were evaluated using the chlorophyll fluorescence parameter Fv/Fm^25^. For ethylene treatment, leaves were sprayed with 100 μM ethylene. Water-deficit stress treatment plants were planted in a pot without water. Fv/Fm values were used to select six time points for AZ sample collection. Samples (approximately 1–2 mm) were cut from each pulvinus-petiole, including the AZs. AZs were collected from the middle of the cassava plants in each pots, and three pots served as a biological replicate. The procedure was repeated 3 times for each time point. AZs that were approximately 1–2 mm in size were frozen in liquid nitrogen for RNA extraction. RNA was purified using the plant RNA reagent from Invitrogen (Carlsbad, CA) to produce a pure and high-quality RNA preparation as indicated by spectroscopic and gel electrophoresis analysis^25^.

### The cassava genome microarray: design, hybridization, and data analysis

To compare the gene expression profiles during ethylene and water-deficit stress-induced cassava leaf abscission, leaf chlorophyll fluorescence (Fv/Fm) values were measured and represented as six time points (T1-T6) as described previously^25^. To verify the reliability and accuracy of differential gene expression profiling, AZs samples were collected with the same Fv/Fm values for both ethylene- and water-deficit stress-induced leaf abscission. Then used time course cassava genome microarray analysis to examine the AZ gene expression during ethylene- and water-deficit stress-induced leaf abscission using the T1 time point as a control. A time series of cassava microarray analyses based on the principle of the “loop design” was performed as previously described^39^. For either ethylene or drought treatment, 18 distinct AZ samples (three biological replicates at each of 6 time points) to be compared, the experimental design included 36 two-color microarray slides for both treatments, allowing three technical replicates of each sample to be observed. The cassava microarray was constructed as previously described^25^,^39^. In detail, Two public databases were used for cassava microarray construction: the great majority of the ESTs originated from JGI database (http://www.phytozome.net/cassava.php) and the minority based on sequences from NCBI with E < 1e-5. Custom-designed 60-mer nimblegen DNA microarrays were synthesized by maskless *in situ* photolithographic synthesis^25^. The fluorescent dye (Cy3-dCTP)-labeled cassava cDNA was produced as previously described using CapitalBio cRNA Amplification and Labeling Kit (CapitalBio). After completion of double-stranded cDNA (dsDNA) synthesis, the dsDNA products were purified using a PCR NucleoSpin Extract II Kit (MN). The resulting cRNA was labeled according to Nimblegen recommendations. The procedures of Array hybridization, washing, scanning and data analysis were performed at CapitalBio Corporation (Beijing, China) according to the NimbleGen’s Expression user’s guide. The expression data of probes were normalized using quantile normalization and expression data of genes were generated using the Robust Multichip Average (RMA) algorithm^25^.

### Time course analysis

To identify differences in *R2R3 MYB* gene expression, statistical analysis was used to screen the microarray data for genes that were differentially expressed in the treatment samples (T2-T6) compared with the T1 control. For comparative analysis, differences in gene expression between samples (T2-T6) and reference (T1) were identified using significant analysis of microarray software (SAM, version 3.02)^25^,^39^. Changes in gene expression exceeding a threshold of <0.5 or >2.0-fold change with a Wilcoxon Rank-Sum test significance level of 0.01 (P < 0.01) and a false discovery rate (FDR) threshold of <1% in the SAM output were considered to be differentially expressed. Time-dependent differentially expressed genes were classified with self-organizing tree algorithm clustering (SOTA) using MeV 4.0 software^25^,^40^.

### GO analysis

GO annotation of gene clusters was performed using BiNGO according to Maere *et al.*^25^,^41^. Significant GO categories were identified using a hypergeometric test with a significance threshold of 0.01 after a Benjamini-Hochberg FDR correction^42^. GO categories were classified by hierarchical clustering using MeV 4.0 software.

### Analysis of cassava *R2R3 MYB* subfamily putative promoter region cis-elements

The putative promoter regions were analyzed for cis-elements according to Wu *et al.*^43^. In detail, the 2-kbp putative promoter regions upstream of each *R2R3 MYB* subfamily coding DNA sequence were examined for cis-elements by using the PLACE website (http://www.dna.affrc.go.jp/PLACE/).

### Real-time RT-PCR validation of *R2R3 MYB* subfamily and the downstream gene expression in cassava

RNA from three independent biological samples were reverse transcribed and used for real-time qRT-PCR with SYBR Green I (Carlsbad, CA) detection on a STEP-ONE system. Gene-specific primers were designed using IDT Primerquest tools (http://www.idtdna.com/Primerquest/Home/Index)^25^. The SYBR Green PCR kit (Applied Biosystems) was used to perform QPCR^44^. The real-time PCR primer sequences are listed in Additional table 6 and 7. To avoid non-specific amplification from other family genes, the primers were designed to span intron-exon boundaries or target the untranslated regions and the primer pairs were confirmed using the cassava genome database to ensure their specificity. The expression ratios of *R2R3 MYB* genes and the downstream genes at each time point are presented using T1 as a control first in *SC5*, and then to cut the T1 control expression ratios with those of *SC5* at each time point. The QPCR procedures were as follows: 10 min of denaturation at 95 °C, followed by 40 cycles of amplification with 15 sec of denaturation at 94 °C, 30 sec of annealing according to the melting temperatures provided in Supplemental Table S2, 35 sec of extension at 72 °C, and the fluorescence data collection at 72 °C. After a final extension at 72 °C for 10 min, the specificity of the amplified product was evaluated by melting curve^44^.

## Additional Information

**How to cite this article**: Liao, W. *et al.* Genome-wide identification of cassava *R2R3 MYB* family genes related to abscission zone separation after environmental-stress-induced abscission. *Sci. Rep.* **6**, 32006; doi: 10.1038/srep32006 (2016).

## Supplementary Material

Supplementary Information

## References

[b1] Qi L., Yang J., Yuan Y., Huang L. & Chen P. Overexpression of two R2R3-MYB genes from Scutellaria baicalensis induces phenylpropanoid accumulation and enhances oxidative stress resistance in transgenic tobacco. Plant Physiol Biochem. 94, 235–243 (2015). 26115549 10.1016/j.plaphy.2015.06.007

[b2] Zhang X. *et al.* The R-R-type MYB-like transcription factor, AtMYBL, is involved in promoting leaf senescence and modulates an abiotic stress response in *Arabidopsis*. Plant Cell Physiol. 52(1), 138–148 (2011). 21097474 10.1093/pcp/pcq180

[b3] Qiu L. H. *et al.* Genome-wide identification of R2R3-MYB genes and expression analyses during abiotic stress in *Gossypium raimondii*. Sci Rep. 6, 22980 doi: 10.1038/srep22980 (2016). 27009386 PMC4806351

[b4] Yanhui C. *et al.* The MYB transcription factor superfamily of *Arabidopsis*: expression analysis and phylogenetic comparison with the rice MYB family. Plant Mol Biol. 60, 107–124, doi: 10.1007/s11103-005-2910-y (2006). 16463103

[b5] Jaradat M. R., Feurtado J. A., Huang D., Lu Y. & Cutler A. J. Multiple roles of the transcription factor *AtMYBR1/AtMYB44* in ABA signaling, stress responses, and leaf senescence. BMC Plant Biol. 28, 13–192, doi: 10.1186/1471-2229-13-192 (2013). PMC4219380 24286353

[b6] Chen T., Li W., Hu X., Guo J., Liu A. & Zhang B. Cotton MYB *transcr*iption factor, *GbMYB5,* is positively involv*ed in plant ada*ptive response to drought stress. Plant Cell Physiol. 56(5), 917–929 (2015). 25657343 10.1093/pcp/pcv019

[b7] Wang R. K., Cao Z. H. & Hao Y. J. Overexpression *of a* R2R3 MYB gene *MdSIMYB1* increases tol*erance* to multiple stresses in transgenic tobacc*o and a*pples. Physiol Plant. 150(1), 76–87 (2014). 23647377 10.1111/ppl.12069

[b8] Chen B. *et al.* Identification, cloning and characterization of R2R3-MYB gene family in canola (*Brassica napus*L.) identify a novel member modulating ROS accumulation and hypersensitive-like cell death. DNA Res. 23(2), 101–114 (2016). 26800702 10.1093/dnares/dsv040 PMC4833418

[b9] Shukla P. S., Gupta K., Agarwal P., Jha B. & Agarwal P. K. Overexpression of a novel SbMYB15 from Salicornia brachiata confers salinity and dehydration tolerance by reduced oxidative damage and improved photosynthesis in transgenic tobacco. Planta 242(6), 1291–1308 (2015). 26202734 10.1007/s00425-015-2366-5

[b10] Wang H., Wang H., Shao H. & Tang X. Recent advances in utilizing transcription factors to improve plant abiotic stress tolerance by transgenic technology. Front Plant Sci. 7, 67 (2016). 26904044 10.3389/fpls.2016.00067 PMC4746321

[b11] Zhu N. *et al.* The R2R3-type M. Y. B. gene OsMYB91 has a function in coordinating plant growth and salt stresstolerance in rice. Plant Sci. 236, 146–156 (2015). 26025528 10.1016/j.plantsci.2015.03.023

[b12] Meng X. *et al.* Physiological changes in fruit ripening caused by overexpression of tomato SlAN2, an R2R3-MYB factor. Plant Physiol Biochem. 89, 24–30 (2015). 25698665 10.1016/j.plaphy.2015.02.005

[b13] Park S. C. *et al.* Overexpression of the IbMYB1 gene in an orange-fleshed sweet potato cultivar produces a dual-pigmented transgenic sweet potato with improved antioxidant activity. Physiol Plant. 153(4), 525–537 (2015). 25220246 10.1111/ppl.12281

[b14] Sun P., Zhu X., Huang X. & Liu J. H. Overexpression of a stress-responsive MYB transcription factor of Poncirus trifoliata confers enhanced dehydration tolerance and increases polyamine biosynthesis. Plant Physiol Biochem. 78, 71–79 (2014). 24636909 10.1016/j.plaphy.2014.02.022

[b15] Al-Attala M. N., Wang X., Abou-Attia M. A., Duan X. & Kang Z. A novel TaMYB4 transcription factor involved in the defence response against Puccinia striiformis f. sp. tritici and abiotic stresses. Plant Mol Biol. 84(4–5), 589–603 (2014). 24293360 10.1007/s11103-013-0156-7

[b16] Park M. R. *et al.* Supra-optimal expression of the cold-regulated OsMyb4 transcription factor in transgenic rice changes the complexity of transcriptional network with major effects on stress tolerance and panicle development. Plant Cell Environ. 33(12), 2209–2230 (2010). 20807373 10.1111/j.1365-3040.2010.02221.x

[b17] Zhou M. *et al.* Changing a conserved amino acid in R2R3-MYB transcription repressors results in cytoplasmic accumulation and abolishes their repressive activity in *Arabidopsis*. Plant J. 84(2), 395–403 (2015). 26332741 10.1111/tpj.13008

[b18] Ding Z. *et al.* Transgenic expression of MYB15 confers enhanced sensitivity to abscisic acid and improved drought tolerance in Arabidopsis thaliana. J Genet Genomics. 36(1), 17–29 (2009). 19161942 10.1016/S1673-8527(09)60003-5

[b19] Ergün N., Özçubukçu S., Kolukirik M. & Temizkan Ö. Effects of temperature - heavy metal interactions, antioxidant enzyme activity and gene expression in wheat (Triticum aestivum L.) seedlings. Acta Biol Hung. 65(4), 439–450 (2014). 25475983 10.1556/ABiol.65.2014.4.8

[b20] Zhao Y. *et al.* The ABA receptor PYL8 promotes lateral root growth by enhancing MYB77-dependent transcription of auxin-responsive genes. Sci Signal. 7(328), ra53 (2014). 24894996 10.1126/scisignal.2005051 PMC4298826

[b21] Devaiah B. N., Madhuvanthi R., Karthikeyan A. S. & Raghothama K. G. Phosphate starvation responses and gibberellic acid biosynthesis are regulated by the MYB62transcription factor in *Arabidopsis*. Mol Plant. 2(1), 43–58 (2009). 19529828 10.1093/mp/ssn081 PMC2639739

[b22] Zhong R., Lee C., Zhou J., McCarthy R. L. & Ye Z. H. A battery of transcription factors involved in the regulation of secondary cell wall biosynthesis in *Arabidopsis*. Plant Cell. (**10**), 2763–2782 (2008). 18952777 10.1105/tpc.108.061325 PMC2590737

[b23] Johnson S. M., Lim F. L., Finkler A., Fromm H., Slabas A. R. & Knight M. R. Transcriptomic analysis of Sorghum bicolor responding to combined heat and drought stress. BMC Genomics. 15, 456 (2014). 24916767 10.1186/1471-2164-15-456 PMC4070570

[b24] Kim J. H. *et al.* Loss of the R2R3 MYB, AtMyb73, causes hyper-induction of the *SOS1* and *SOS3* genes in response to high salinity in *Arabidopsis*. J Plant Physiol. 170(16), 1461–1465 (2013). 23809151 10.1016/j.jplph.2013.05.011

[b25] Liao W. *et al.* Reactive oxygen species regulate leaf pulvinus abscission zone cell separation in response to water-deficit stress in cassava. Sci. Rep. 6, 21542; doi: 10.1038/srep21542 (2016). 26899473 PMC4761936

[b26] Stracke R., Werber M. & Weisshaar B. The R2R3-MYB gene family in Arabidopsis thaliana. Curr Opin Plant Biol. 4, 447–456 (2001). 11597504 10.1016/s1369-5266(00)00199-0

[b27] Katiyar A. *et al.* Genome-wide classification and expression analysis of MYB transcription factor families in rice and Arabidopsis. BMC Genomics. 13, 544, doi: 10.1186/1471-2164-13-544 (2012). 23050870 PMC3542171

[b28] Du H. *et al.* Genome-wide analysis of the MYB transcription factor superfamily in soybean. BMC Plant Biol. 12, 106, doi: 10.1186/1471-2229-12-106 (2012). 22776508 PMC3462118

[b29] Wilkins O., Nahal H., Foong J., Provart N. J. & Campbell M. M. Expansion and diversification of the Populus R2R3-MYB family of transcription factors. Plant Physiol. 149, 981–993 (2009). 19091872 10.1104/pp.108.132795 PMC2633813

[b30] Du H., Feng B. R., Yang S. S., Huang Y. B. & Tang Y. X. The R2R3-MYB transcription factor gene family in maize. Plos One 7, e37463, doi: 10.1371/journal.pone.0037463 (2012). 22719841 PMC3370817

[b31] Moschou P. N. *et al.* Spermidine exodus and oxidation in the apoplast induced by abiotic stress is responsible for H_2_O_2_ signatures that direct tolerance responses in tobacco. The Plant Cell 20, 1708–1724 (2008). 18577660 10.1105/tpc.108.059733 PMC2483379

[b32] Chen D. *et al.* A wheat aminocyclopropane-1-carboxylate oxidase gene, TaACO1, negatively regulates salinity stress in *Arabidopsis thaliana*. Plant Cell Rep. 33(11), 1815–1827 (2014). 25048023 10.1007/s00299-014-1659-7

[b33] Rowan D. D. *et al.* Environmental regulation of leaf color in red *35S:PAP1 Arabidopsis thaliana*. New Phytol. 182(1), 102–115 (2009). 19192188 10.1111/j.1469-8137.2008.02737.x

[b34] Xie Y. *et al.* Hydrogen sulfide delays GA-triggered programmed cell death in wheat aleurone layers by the modulation of glutathione homeostasis and heme oxygenase-1 expression. J Plant Physiol. 171(2), 53–62 (2014). 24331419 10.1016/j.jplph.2013.09.018

[b35] Gubert C. M. *et al.* ASYMMETRIC LEAVES1 regulates abscission zone placement in *Arabidopsis* flowers. BMC Plant Biology 14, 195 (2014). 25038814 10.1186/s12870-014-0195-5 PMC4223632

[b36] Shi C. L. *et al.* *Arabidopsis* Class I KNOTTED-Like Homeobox Proteins Act Downstream in the IDA-HAE/HSL2 Floral Abscission Signaling Pathway. The Plant Cell 23 **(7)**, 2553–2567 (2011). 21742991 10.1105/tpc.111.084608 PMC3226213

[b37] Li M. Y. *et al.* Genome-wide analysis of AP2/ERF transcription factors in carrot (Daucus carota L.) reveals evolution and expression profiles under abiotic stress. Molecular Genetics and Genomics 290 **(6)**, 2049–2061 (2015). 25971861 10.1007/s00438-015-1061-3

[b38] Charfeddine M. *et al.* Genome-Wide Analysis and Expression Profiling of the ERF Transcription Factor Family in Potato (Solanum tuberosum L.). Mol Biotechnol. 57 **(4)**, 348–358 (2015). 25491236 10.1007/s12033-014-9828-z

[b39] Breeze E. *et al.* High-resolution temporal profiling of transcripts during Arabidopsis leaf senescence reveals a distinct chronology of processes and regulation. The Plant Cell 23 **(3)**, 873–894 (2011). 21447789 10.1105/tpc.111.083345 PMC3082270

[b40] Li P. *et al.* The developmental dynamics of the maize leaf transcriptome. Nat Genet. 2010, 42 **(12)**, 1060–1067 (2010). 10.1038/ng.703 21037569

[b41] Maere S., Heymans K. & Kuiper M. BiNGO: a Cytoscape plugin to assess overrepresentation of gene ontology categories in biological networks. Bioinformatics 21 **(16)**, 3448–3449 (2015). 15972284 10.1093/bioinformatics/bti551

[b42] Benjamini Y. *et al.* Controlling the false discovery rate: a practical and powerful approach to multiple testing. J R Statist Soc B. 57, 289–300 (1995).

[b43] Wu H. *et al.* Genome-Wide Analysis of the AP2/ERF Transcription Factors Family and the Expression Patterns of DREB Genes in Moso Bamboo (Phyllostachys edulis). Plos one 10 **(5)**, e0126657 (2015). 25985202 10.1371/journal.pone.0126657 PMC4436012

[b44] Shi Y. H. *et al.* Transcriptome profiling, molecular biological, and physiological studies reveal a major role for ethylene in cotton fiber cell elongation. The Plant Cell 18, 651–664 (2006). 16461577 10.1105/tpc.105.040303 PMC1383640

