# Supplementary material for: Genome-wide identification of cassava R2R3 MYB family genes related to abscission zone separation after environmental-stress-induced abscission
Source: Sci Rep. 2016 Aug 30;6:32006. doi: 10.1038/srep32006 (PMC5004182; doi:10.1038/srep32006)
Supplement: Supplementary Information [file srep32006-s1.pdf]

Supplemental Information

Title: Genome-wide identification of *R2R3 MYB* family genes related to abscission zone separation exposure to environmental stress induced abscission in cassava

Authors:

Wenbin Liao

Institute of Tropical Bioscience and Biotechnology, Chinese Academy of Tropical Agricultural Sciences, Haikou 571101, China

Yiling Yang

Institute of Tropical Bioscience and Biotechnology, Chinese Academy of Tropical Agricultural Sciences, Haikou 571101, China

Yayun Li

Institute of Tropical Bioscience and Biotechnology, Chinese Academy of Tropical Agricultural Sciences, Haikou 571101, China

Gan Wang

Institute of Tropical Bioscience and Biotechnology, Chinese Academy of Tropical Agricultural Sciences, Haikou 571101, China

Ming Peng

Institute of Tropical Bioscience and Biotechnology, Chinese Academy of Tropical Agricultural Sciences, Haikou 571101, China

Email: mingpengcatas@gmail.com

Additional file 1. The amino acid sequences of 166 R2R3 MYB genes form cassava

>Manes.01G074400

MGRQPCCDKLGVKKGPWTAEEDKKLINFILTNGQCCWRAVPKLAGLRRCGKSCRLRWTN
YLRPDLKRGLLTEAEEKLVIDLHARLGNRWSKIAARLPGRTDNEIKNHWNTHIKKKLLKM
GIDPVTHEPFHKEAKTEESSSTSRPDNLLPESGSTTNNSSLHENDGIVNSEENSSSPHENCCT
DESTLLDSICNDETLLNSLWMDEPPLVDASWNNNINPPATDDAKNGEMGYPYPYPLWEDN
CTWLFDCQDFGVHDFGFDCFDNLEFKSLNSLEMEEKH
>Manes.02G034300
MGRQPCCDKLGVKKGPWTAEEDKKLINFILTNGQCCWRAVPKLAGLRRCGKSCRLRWTN
YLRPDLKRGLLTEAEEQLVIDLHARLGNRWSKIAARLPGRTDNEIKNHWNTHIKKKLLKM
GIDPVTHEPFHKEAKTEEESSSISQTDNLLPESANSNNNNSSSMQENDGIVNSEENTSSPHEN
CCSNESILIDSICKDETLLNSLWMDEPPLVDSSWNNNNPPAMGNTNYNGEMGYPYPSWED
NCSWLLDCQDFGVHDFGFDCFDNLEFKSLNTLEMGDKH
>Manes.06G136500
MGRQPCCDKFGVKKGPWTAEEDKKLVNFILTHGQCCWRAVPKLAGLRRCGKSCRLRWTN
YLRPDLKRGLLNEAEEQLVIDLHARLGNRWSKIAARLPGRTDNEIKNHWNTHIKKKLIKM
GIDPVTHEPLHKQANPEAAPCKNIKYDGDQQEIPNNNYAQASSFSSDSSSTTLTESSEPVSD
DDPLMSYILSNTFLEDSTWDFHFPVMREEYCEFGMSSSEGIGDDYFGLGRFNDVDLSALD
MSNKH
>Manes.14G034400
MGRQPCCDKFGVKKGPWTAEEDKKLVDFILTHGQCCWRAVPKLAGLRRCGKSCRLRWTN
YLRPDLKRGLLNEAEEQLVMDLHARLGNRWSKIAARLPGRTDNEIKNHWHTHIKKKLIKM
GIDPVTHQPLHKQDNKGAAASHDVIYYDHQPNFSGDQQQISKNNCEAHASSSCNTTPTESS
EPSSNDDPLMSYIFSDSFLEDSKWNFPVEYCEFGMFSPEGNCPWFLEHKDIGDDCFGL
>Manes.02G077400
MGRQPCCDKVGLKKGPWTAEEDKKLINFILTNGQCCWRAVPKLAGLLRCGKSCRLRWTN
YLRPDLKRGLLSEYEEKMVIDLHAQLGNRWSKIASHLPGRTDNEIKNHWNTHIKKKLRKM
GIDPLTHKQLPTTETSQPQEPQQVQEQKQAQSCSTIAMPEPELEHKKETETSIQSSITEESRVE
EDKSIRSTFETMELMNNGFCVDEVPLIEPHEMLVPCAASSSSTTSSSSPSSSSHGSNNNFLED
LQFSDFEWPDNDIDLWGDDLSSCWDLLMNDADSDRKQAAAIDHHPPPINQCPRMVLDQES
WTYGIL
>Manes.13G013500
MGRKPCCDKVGLNRGPWTIEEDQKLMNFILSNGIQCWRLVPKLAGLLRCGKSCRLRWMN
YLRPDLKRGALTEAEEDQIIELHSRLGNRWSKIAAHFPGRTDNEIKNQWNTKIKKKLKLLGI
DPKTHKPVEKEKDFASEEARQQPAETSREELKFQDNNPGFMGNNQIENNQVDQCCSPGQE
SNLVQKSETMITDIDAAQSESFSSTSMQEGSHQHWIDNLEDYLLSWDWFSNLEEIFPLDTH
Q
>Manes.15G155900
ASYSSSLSADESNNVSFGESSSVQDMDSVLSWGSFNHLVDDIFFMENSQQCNIPTDSVFKY
CDNEIKNHWNTRIKKRLRQMGLDPVTHKPIEQMNNETTPVSHDDQAMISDCLVTKKADEK
RDGEDEIVAMDDEKSDLLSNYEMLCGSLDLGSWINQLETNTMGRQPCCDKIGLKRGPWTI
EEDHKLMNFILNNGIHCWRMVPKLAGLLRCGKSCRLRWINYLRPDLKRGSFTEMEENQIIQ
LHSRLGNRWSKIASHFPGRT
>Manes.17G107800
MGRQPCCDKIGLKRGPWTIEEDHKLMNFILNNGIHCWRMVPKLAGLLRCGKSCRLRWINY
LRPDLKRGGFTEMEENQIIQLHSRLGNRWAKIASHFPGRTDNEIKNHWNTRIKKKLKHIEQT

QMSINGGNNETIPERIKEENMEIKSQDDQANLISTDDKSKGGKDDELVSMEETSELLSNYE
MLNGSMDMGSWIKQLETNDTTSYSSSLSVDENKNLSIGESLSLEDMDSILSWDSFNYHLLD
DIFFLENTQYCNIPADSVPKYC
>Manes.01G235800
MGRHSCCYKQKLRKGLWSPEEDEKLLRHITKYGHGCWSSVPKQAGLQRCGKSCRLRWIN
YLRPDLKRGTFSQQEENLIIELHAVLGNRWSQIAAQLPGRTDNEIKNLWNSCLKKKLRQRGI
DPVTHKPLSEVENNGQDKNPPANKSQEKASGVSNELNLLEANNSKPGPTSQEKTQSYHLE
GKGSSNSKTMSSNTNNHSNNSNLMSPISNKDFFLERFATSHHEGSTTNCQPSDLVGHFPLQQ
LNYASNSRLVTNSIPSHWFTQTSKSLDMNSEFSSSSIPTILPPTTSSFLSTSMPFKPSVTVPSDD
PSLASFPINSSRFWEAVALSNNSNSSTGSSGNAELQSTFFESTIFSWGLGDCSSTEKEGQNQL
MGSQQEDVKWPEYLHNPLIMAAALQNQSLPQSLYNEIKSETHFLTQNSSAVWPQNQQQQE
PFQNSDICPKDIQRLTAAYGHI
>Manes.05G007400
MGRHSCCYKQKLRKGLWSPEEDEKLLRHITKYGHGCWSSVPKQAGLQRCGKSCRLRWIN
YLRPDLKRGTFSQQEENLIIELHAVLGNRWSQIAAQLPGRTDNEIKNLWNSCLKKKLRQRGI
DPVTHKPLSEVENNGEDKNPPANNVQDKASGVSNELNFLQANNSNRGQSLQEKKRSPASA
QAYQLEGQGSNSKTMSSNANNHRNNSNLLTPVSNKDFFLERFATSHQEGSTSNCQPSDLHF
PLQQLNYASNARLITNSIPSLWFTNTSKPLDMNSEFSSSSIPSILPPTTSSFLSTPLAFKPSVTVP
SDDPPLASFPISSSRYWEAGAPSNNSNSSTGSSGYTELQTNSSFFENTLFSWGLGDCSSTEKE
GQNQLMGSQQEDVKWPEYLHNPLIMAAALQNQSPQSLYNEIKSETQFLSENSSGMWPHN
QQQQEPFQTSNICAKDIQRLTAAYGHI
>Manes.09G182000
MGRHSCCYKQKLRKGLWSPEEDEKLLNYITKHGHGCWSSVPKLAGLQRCGKSCRLRWIN
YLRPDLKRGAFSQQEENLIIELHAVLGNRWSQIAAQLPGRTDNEIKNLWNSCIKKKLRQRGI
DPNTHKPLSEVENDYKEKPPTNNKNNDKAFTVSNVNELNLIEPANSKPSTVSSSSKLTTNN
DHSSSNLTPTPPTQEFFLDRFASSHDSSTTSCRPSDLVGYFSFQKLSYKPNICFNSNSSSSEMIS
EFNSCMTPTILPPISSSMFQTSITVKPSISLPSDNPNSIGSCEVNGIQTWETSGFNNSGSSCSNG
SSSSIELQNNTTFFDTNTFSWGLPDCGKPGEEAHLRSLENEQDIKWSEYLSTPFLIGTTAIQN
QTSQPMYSEVKPETHFITEGSSTSWQQNHQHHQYHHHSQTSQPSENYAKNLQRLAVAFGQ
TF
>Manes.08G106900
MGRHSCCYKQKLRKGLWSPEEDEKLLNYITKHGHGCWSSVPKLAGLQRCGKSCRLRWIN
YLRPDLKRGAFSQQEENLIIELHAVLGNRWSQIAAQLPGRTDNEIKNLWNSSIKKKLRQRGI
DPNTHKPLSEVENDKEKQLANSKNNEKASFISNNELNLIETANSKPSTISSSSKITSNNDHSS
NLTPTPSTQEFFLDRYAASHESSTTSCRPSDLVGYLPFQKLSYRPNIGLSVNPNTSICFNPNSS
SSEMISEFNSSTTPSILPSISNSMFQTPIRVKPSVSLPSDNPSVRSCDVSGVQNWEASSFSNNG
SSCSNGSNSGIELQNTTTFFESNAFSWGLADCGKPGEEAQLRSLENDTEDMKWSEYLSTPF
LLGTAIQNQTSQPMYSDVKPETNFITEGSSGSWQQNHQHHHQQVSQPSDIYAKDLQRLAVA
FGQTL
>Manes.06G109000
MGRHACCLKQKLRKGLWSPEEDEKLYNYITRFGVGCWSSVPKLAGLQRCGKSCRLRWIN
YLRPDLKRGMFSQQEEDLVISLHEVLGNRWAQIAAQLPGRTDNEIKNFWNSCLKKKLMKQ
GIDPATHKPITEALEVKEEKICVDKVSLQIPQSKALPAVANTATQEPTFLINGTTCYSNGLTEN
SGEQFMNKQIFDPFSYYEFSAGIEPGRYNSSIPGLQRPNLRPFDQTQFETSSIFSFTSMPSLTSF

DHGSVSATDFSDNSGSRMSSMFLNEAKESSSNCSNISSYTGTGHQMNCMMENNAAFSWD
ADNKIDSMFQFPMNGIKMEELRPSPWQEGQLHTRNSVYFSSCPLTSLSEDLTGVNFEAYHQI
>Manes.14G061700
MGRHSCCLKQKLRKGLWSPEEDEKLYNYITRFGVGCWSSVPKLAGLQRCGKSCRLRWINY
LRPDLKRGMFSQQEEDLIISLHEVLGNRWAQIAAQLPGRTDNEIKNFWNSSLKKKLMKQGI
DPTTHKPISDALEVKEEKNCTDKASLQIPQSKTLPILSNSAQEPTFLINDTTYYSNGLTETSR
DQFMDKQAYDPLSYFEFSAGVEPSGYNSSVPALQHPNLRPFDQNQFETSSDFAFTSMPSLTS
FDHGSMSATDFSDNSASRMSSMFLNEAKESSSNSSNISSYTGYQMNSMVENNAAFSWDGE
NKIDSVFQFQVNGIKTEELRPSPWQEGQLHPQNSIDFSSYPLTSLSEDLTGANFDVFHQI
>Manes.01G093300
MGRSPCCDKVGLKKGPWTPEEDQKLLAYIEEHGHGSWRALPAKAGLQRCGKSCRLRWSN
YLRPDIKRGKFSLQEEQTIIQLHALLGNRWSAIATHLPKRTDNEIKNYWNTHLKKRLAKMG
IDPVTHKPKNDALLSSDGQTKDAANLSHMAQWESARLEAEARLVRESKLRSHSFQHQISST
GYVSSSGSASTSASASAQPQRSLDVLKAWNGGWSKSSEGNTVGGPNIAGIGGDLESPTSSL
TFSENAPPQIINSSGTGGQNSISLIELVGTSGSSETGIIKEEGEHDWKTLGNSDHHMPENSVSF
TSSLHHPDMSISMEGPWTPESLKLNSSHLHIGNVMEEGFTNLLLSDTGVRTLSESGKDSDNS
GSDYYEDNKNYWNSILNLVNSSPADSPIF
>Manes.02G047900
MGRSPCCDKVGLKKGPWTPEEDQKLLAYIEEHGHGSWRALPAKAGLQRCGKSCRLRWTN
YLRPDIKRGKFSLQEEQTIIQLHALLGNRWSAIATHLPKRTDNEIKNYWNTHLKKRLAKMG
IDPVTHKPKNDALLSSDGQSKNAANLSHMAQWESARLEAEARLVRESKLRSHSFQEQLSP
TGYVSGSGSGSVSASASTSAQPQQCVDVLKAWNGGWSKSSEGGNGLNTTGIGGDHHLESP
TSTLTFSENAPPLIMNSSGTGGENSIPMIELVGTSGSSETGIIKEEGEHDWKNLRNSSHHYHH
HHLMHENTVSLTSSLHHPDMTISMEGPWTPEFLKLSSSHIHVGNVDIEEGFTSLLLNDSGER
SLSDSGKDSDNSGGSGNDCYEDNKNYWNSILYNLV
NSSPTDSPMF
>Manes.08G058000
MVRSPCCEKGGLKKGPWTPEEDQKLLAYLKEHGHGSWQALPAKAGLQRCGKSCRLRWT
NYLRPDIKRGKFSLQEENCIIRLHALIGNKWSAIATHLPQRTDNEIKNYWNTHLKKRLDKM
GIDPGTHKPKVDAFGSGSGVLKDAAHLSHMAQWESARLEAEARMVRESKLASNQLGFPS
PHQLFTTTTAAAAAVPPVRPKCLDVLKAWQGIVSGKFFVSSDSLESPTSTLNFSAVNSVVEF
HQQSSVAIPPPQFPVCNITCKGEMGEDVSNQMAPQVKEALDGSISVHGMSAYTTENAWAL
DSFEAAANENASIGNIAEGLSAIVPYNCGEQNASMPGEKTATSESCGGGGNLEDLQGDYW
NSLLLNLVDDPLLQSVMITYQGK
>Manes.09G007100
MVRSPCNEKGGLKKGPWTPEEDQKLVAYIEEHSHGSWQALPAKAGLRRCGKSCRLRWINY
LRPDIKRGKFSSQEEQTIIQLHALLGNKWSAIATHLPRRTDNEIKNYWNTHLKKRLDRMGID
PMTHKPKADAFGSGSSQYKDAANLSHAAQWESARLEAEARLVRESKRPLHKQFGFSSSAA
SASSLHLPKFSPPSKATAAALSVRPKCLDVLRAWQGMVSGGGLESPTSTLNFPENALLTPVV
ASIPQLQFPTCNITCKGGIDEDASHSENEWKSFEKSNQMAHQVKETIDESNILHEMTMYISE
NAWVYDSFRATASDIMGNIVEGVSDIMAYNNGEQNSSMAGENVTTTSQSCCANLEDMQG
NYWNSLLLNLVDGPVPFGSPVL
>Manes.17G065700
MVRSPFCYSSSLKKGPWTPEEDEKLVDYINRNGHESWTNLPKLAGLNRCGKSCRLRWINY

LRPDIKRGKFSQEEERLIVNLHSVLGNKWSRIATHLPGRTDNEIKNFWNTHIRKKLLQMGID
PNTHRPRTDFNHLSNLSFLLFGNLTSPWNNVFKLQHSDVAHLINIRLLHNLLQITNTRSFPSIT
VSGGTQNLNPLEQLLNKTATTSLYIEEPFPRTEDLPLQAPTEVTDAANIWAALEGEMLLDLD
MENNKLMNSCCDTQIGNSLLDQLISASSSSSPESSMVNQMESNGNPLDDNEGSGSGSGSGS
PTFSVFDSWEKLIDDESGSFWNDIQE
>Manes.02G152500
MGRSPGCDESGLKKGPWTPEEDQKLVDYIQKHGHGSWRALPKLAGLNRCGKSCRLRWTN
YLRPDIKRGKFSQEEEQTILNLHSVLGNKWSAIASHLPGRTDNEIKNFWNTHLKKKLIQMG
FDPMTHQPRTDLLASLPHLMALLDLKDLMNHRLLDDHSMRLQAEAVQLTKLQYLQYLLQ
SENSIASNSYGQNGIADVEILNLLNQIPAIKETPFLNSSQFENPASSYFFGLATSQPLHYSNQL
PQMSDPQVLFNNQPSLNSEIGQAATLTTMVSQGDNNNNIDPSDSLWVLPSPTSSIPPTLPETS
MSNPGDAFSAASSSGGGTYSHWPEIFLDDDSIMHEIS
>Manes.18G067400
MGRSPCCDENGLKKGPWTPEEDQKLVDYIQKHGHRSWRALPKLAGLNRCGKSCRLRWTN
YLRPDIKRGKFSEEEEKTILNLHSVLGNKWSAIASHLPGRTDNEIKNFWNTHLKKKLIQMG
FDPMTHQPRTDIFASLPHLVALLNLKDLLDRHPLDEHAMRLQAEAIQLAKLQYLRYLLQSA
TSITSNSYTQNGTTDMEILSLLSQIPVMKETPLLNSSQLENINPASSNPFGIATSQPLHYSNLL
PQLSDPQVPFNCQPSLNNEMGQAATLSAMLNDGDNSNPSDSSWVLRSPTPSIPPTVTDTSIS
NNLGDASSTSSYGGGTSSYWPEFFLDDSIMHQIS
>Manes.03G117500
MGKSACCDNNGLKKGPWTAEEDQKLMDYIQKHGHGRWRTLPKNAGLRRCGKSCRLRWT
NYLRPDIKRGKFSFEEEEAIIQLHSILGNKWSAIASRLPGRTDNEIKNYWNTHIKKRLLRMGI
DPVTHKPRLDLLQLYSVLNSSIYNSSHQINISNLLGIGPTLNPNLLNLATSLLSSQSNSQDTSP
ENVQENQLDNPQIHQNQFQSLQPNQYQACIDSSAQFLNETQLLESNLEQLNSTSQANSIFQ
NSLQWEETRNFPDMGESLTPYCGYYEMGNQDMIKSSFENLSNMGFSSLISTASSSTTPLQSS
STTYVNGGIEDERDSFCSNILMYDIPNNLDANNGLL
>Manes.15G081900
MGKSACCDKNGLKKGPWTAEEDQKLMDYIQKHGHGKWRTLPKNAELKRCGKSCRLRWT
NYLRPDIKRGKFSAEEEEAIIQLHGVLGNKWSAIASRLPGRTDNEIKNFWNTHIKRRLLRMG
IDPVTHKPRLDLLQLYSLLNSSLCNSSHQINISSLLGIGSMLNPNLATSLLSTQSNSHDISPGN
FQEHQNDNSQVQNQFQSLQPNQIMDSSPQFLNETQLLQANLEQISTSQTNFNCLNSLQSLW
QETGKSANMGESLMPNCGYNEMSNQAMMKCSFENISNLGLNSSLLSTPSSSTTPLQSSSTT
YVNGGTEDERDSYCSNIMMFDIPSALDVNGLL
>Manes.12G082000
MGRAPCCDKNGLKKGPWTPEEDNKLISYIQLHGPGNWRALPKNAGLQRCGKSCRLRWTN
YLRPDIKRGRFSFEEEETIIQLHSIMGNKWSSIAARLPGRTDNEIKNYWNTHIRKRLLRNGID
PVTHSPRLDLLDLSSILGSALCNPSLLNLSSLLGNHAILNPELLRLATILSSLKQENTEMFLQN
LQDNQLLSSLVQNQFPLSQVSQFQNPVEEATSAPFLSPTQLMQTDLGGLSCLNSLENSVPSS
LSDCLVSQPNSVSCNTNPPIPDKLVENSGLQPITNGCQNYSIESVLSTPLSSPAPLNSSSTFVN
SSSPEDERESYCSSLFKFEIPESLNMDDFL
>Manes.13G152600
MGRAPCCDKNGLKKGPWTPEEDHKLITYIQLHGPGNWRTLPKNAGLQRCGKSCRLRWTN
YLRPDIKRGRFSFEEEETIIQLHSIMGNKWSAIAARLPGRTDNEIKNYWNTHIRKRLLRNGID
PVTHAPRLDLLDLSSILGSALCNPSLFNLSSLLGTQTILNPEVLRLATTLSSLRQENPEMFLQ

HLQDNQVLSSQVQNQVSLSQANQFQNTIQEPTSSQFLSPTQLMQTDVGGLSCQNSLQNVV
PSSLDDCLVSQVPNFVSCNTNPSIPDKLAGNSGFQPINNSCQNYSIESVLSTPLSSPAPLNSSS
TFVNSSSTEDERESYCSTLFKFEIPESLDMDDFL
>Manes.16G019700
MGRAPCCDKNGLKKGPWTPEEDQKMIDFIQKHGYGNWRTLPKKAGLQRCGKSCRLRWT
NYLRPDIKRGRFSFEEEETIIQLHSILGNKWSAIASRLPGRTDNEIKNYWNTHIRKRLLRMGI
DPVTHSPRLDLLDLSSIFGSSFYNSSQMNISRFLGIQPMVNPELLRLATSIISSQRENHNFVPQ
NCQENQLCNLQIQNQYHPVIQADQFQSQVQEMPSCTTLANPFVHFSGETQLMDEMNHQE
WQRDDEMASNLTDNFMTLQSYENYYGASDQTAMMDPSCETTSTFISKNSNQNLSLASVLS
TPSSSPKPLNSNSTYINCSSSTEDERESYCSNMLKFEIPDILDVSNFM
>Manes.17G033800
MGRVPCCDKNGLKKGPWTPEEDQKLIDYIQKHGYGNWRTLPKNAGLQRCGKSCRLRWT
NYLRPDIKRGRFSFEEEEAIIQLHGIWGNKWSAIAARLPGRTDNEIKNYWNTHIRKRLLRM
GIDPVTHSPRLDLLDLSSILGSSLYNSSQMNISRFLGMQPVVNPELLQLASSIISTQRQNQNF
VSQNGQENRLCDLSPQNQYHPVVQASQFQSQVQEMPTCTTLTTPFVPFSNETQLMDPNITD
LCSQNSQVNHQEWQSDTGMASNLTDYMTTLPSLNDYYVSDQTAIMDPSSETSSAFISNNSN
QQNFSFASVLSTPSSSPTPLNSNSTYISGSSSTEDERESYCSNMLKFEIQDILDVNFM
>Manes.S062600
MVRTPSCDKSGLRKGTWTPEEDRKLTAYVTRYGCWNWRQLPKYAGLSRCGKSCRLRWM
NYLRPNIKRGNYTGEEEEIILRLHESLGNRWSAIAAQLPGRTDNEIKNYWHTNLKKRKSND
TAKQDFIGLSQPKKTRKVKIQPNIIDPLNPATTQIVESSTLCPTPSTSDFSSSTADNAAVTSSCS
DLKSDDEFAFLEAYEAPCGNFWTEPFLADNYYMPFEFLAPSLDPLSDPLLDGELLYSYDVC
DYGILNWQL
>Manes.12G085600
MVRSPFFDKNGLKKGAWSPEEDYKLRTYIVRYGHWNWRELPKFAGLQRCGKSCRLRWM
NYLRPGVKHGKYSKEEEDLIIKLHNQLGNKWSRIAAELPGRTDNEIKNHWHTHLKKRSKE
SQRESGLKEKYSIEQSSETSQLNGDLKVESFVPNTPSHAILESFPLSPAPSSSEISHWTSDSNL
ATLSSTSTSSDWIAAEDSLPSFETFENTSEDFWTQPFVADQDGYKFPMLDEGLISSFLTSYED
SIDLFYKVMQELPGN
>Manes.S062500
MVRAPFFDKSGLKRGAWSPEEDEKLRAYVLRYGHWNWRKLPKFAGLSRCGKSCRLRWM
NYLRPGVKHGSYSQQEDDLIMKWHQDVGNKWSLIAAKLQGRTDNEIKNYWHSHLKKIAK
NKQNASGLKKVASETSQSDARENAETHEAQVFFPNSPSHLILESSPLSPETYSGKFSPITSDS
AHVTPQDQSWSAESFRSFEESLGDFWTEPFVADSTYNQDVGFMPLSVFDYDDNVELLYQV
MQELPGN
>Manes.12G085900
MVRTTAVDKNGLKKGSWNKEEDDKLRDYIQKYGHWNWRELPKYAGLSRCGKSCRLRWV
NYLRPGVKHGNFSKEEDDLIIQLHQELGNKWSRIATKLPGRTDNDIKNHWHTHLKKRGKQ
SRASKAEVEAQSTETPESLTSPSKKLEAADQSDVVNKTPSLPTILESFPLSKESSYRSSASAG
LNFFAEDSLTSMEIFGESGEDFWTRPFVADNAYDQDDYISSYTLYYDDNLDFFYQVLQELPD
NYM
>Manes.12G032200
MVRAPCCEKMGLKKGPWTAEEDQILVNYIQQYGHGNWRALPKQAGLLRCGKSCRLRWIN
YLRPDIKRGNFTREEEDTIIKLHEMLGNRWSAIAARLPGRTDNEIKNVWHTHLKKRLKQNP

GTPEIKTTSIAMISRVAQESKQESELVTLSNLPGPESPEGVEYRSISPQQCSSSEISSVITGDDA
SHNNMSSMKVEESDDFPEMDENFWSEVLSSDNSSSGSNFPAAAQFQIPFSTVGNAMEPAVQ
YGYDSMDFWYNLFTKAGESPELPEI
>Manes.13G033900
MVRAPCCEKTGLKKGPWTPEEDQILISYIQQYGHSNWRALPKQAGLLRCGKSCRLRWINY
LRPDIKRGNFTREEEDTIIQLHEMLGSRWSAIAARLPGRTDNEIKNVWHTHLKKRLLKQNP
GDPKIKRRSIDMSRVDKQLKTEPDAKLVNLSNHTGSESSDGLEYRPISPQQCSSSEISSVITG
DYANNNMSSMKLELWDDFPEMDENFWSEVLSSDQSTSASDFPATAEAVQFHIPFSTLENAM
EPAAQYACNSSMHDSMEFWYNLFTRAGKSPELSEI
>Manes.03G164200
MVRAPCCEKVGLKRGPWTPEEDKILISHIQNYGHSNWRALPKQAGLLRCGKSCRLRWINY
LRPDIKRGNFTEEEEEAIIKLHEMLGNRWSAIAAKLPGRTDNEIKNVWHTHLKKKLKQKQD
FKSNNKQLHETVPKCDEVSVGNQSDSENYSNVPTLTGLHEIPGMHSPMSPQPSSSSSDHST
VTETSGATAETDNITFENMDSSEIIFPVIDEDFWSDEAGLASEIMIPSGFYGNGPTSIDDDMDF
WYNLFVKSGGIEELL
>Manes.15G040700
MVRAPCCEKMGLKKGPWTPEEDKILISHIQKYGHSNWRALPKQAGLLRCGKSCRLRWINY
LRPDIKRGNFTEEEEETIIKLHEMLGNRWSAIAAKLPGRTDNEIKNVWHTHLKKKLKQKQD
SNNQQLYVTVPKCEDLSISNPSESENSNIPALPGQYESPGQSAMSPQPSSSDLSTATYTSGAT
AETDDVKVETMDSSEIYFPVIDQDFWSEAELIDSSSMELASDMVAASGFNGNCLSIDDDMD
FWYDLFVKAGDIEELL
>Manes.08G138700
MVRIPCCENMGLKKGPWTPEEDQILVSYIQRFGHGNWRALPKQAGLLRCGKSCRLRWINY
LRPDIKRGNFSNEEEEAIISLHQILGNRWSAIAAKLPGRTDNEIKNYWHSHLKKRLEEKQAN
PSPSSSAGNCTKTSKMAVINNARSSHQPENFPTHIGIHHLLHAQVFPQEQSQEPLATLEATNG
FNTVNDTQFWYDLFMKAGNSKEVHGNL
>Manes.09G148500
MVRIPCCENMGLKKGPWTAEEDLILVSYIQRFGHSNWRALPKQAGLLRCGKSCRLRWINY
LRPDIKRGNFSNEEEETIIKLHQIIGNRWSAIATKLPGRTDNEIKNYWHTHLKKRVDGKQATP
STSSSADQTTKTSSNIKAVINNARSSQQPESFPTHQNIRYATEEKSQEPVATAVEAINGFSTVN
DTEFWYDIFMEAGNSREMHGDLRV
>Manes.03G201700
MGRAPCCEKVGLKKGRWTAEEDEILTKYILANGEGSWRSLPKNAGLLRCGKSCRLRWINY
LRADLKRGNITKEEEETIVKLHTALGNRWSLIAAHLPGRTDNEIKNYWNSHLSRKIYSFSKH
GSLPTAGTTTVSINIAKIAGPRKSRSATRKRHKENTSISVSTTPKTETLTEAVVPEGSEPPPSSN
DNSTGSFDHGSQIMGLVPESSTSELKGSSSCIDNGEKLNAVAESESWGPYEWLDSEINRLKY
VLECEPVNPSGDFDTIDDKEREEKKVDELGRGDGEVMGPETVAAANESCSSNGWSPNAEG
GELYNSGSSISFDEDWYDLSFDWDSTGSIDDSAIIKELWDEGDKFMSWLRG
>Manes.15G006600
MGRAPCCEKVGLKKGRWTAEEDEILTSYILVNGEGSWRSLPKNAGLLRCGKSCRLRWINY
LRADLKRGNITKEEEETIVKLHSTLGNRWSLIAAHLPGRTDNEIKNYWNSHLRRKLYSFSN
NNSLSSSINIAKLAALRKSGGGRSATRKHKPNTCMSAPKPNNIETLAEAVPDPPSPNNTSDE
GNQRMGLVTESFTSEVENCRRGLGNGGVQGCSWKEKDETFSAVGVTCPRKEGQTEDWGP
HVLLDSEINILKYALEGEDVDPSGNHIIDTLNKEREIGGSEERGHEVMGPDRVAANEQERAS

TSTATTAWSSNAETGELYNCGSSQFDEEWYNLSFDWESIGGIDDTVIRELWDEGEKIMSWL
WD
>Manes.01G226200
MGRAPCCEKVGLRKGRWTAEEDEKLMKYIQANGEGSWRSLPKNAGLLRCGKSCRLRWIN
YLRTDLKRGNISSDEEEIIFKLHASLGNRWSLIACHLPGRTDNEIKNYWNSHLSRKVHIFRKP
GNENRQLVIDTANLGTRTGTSKRGKTPGRSCAVKNNNKSQLHKQPASLTNETDEYSTGFSK
DDGIIPMPQTPVLEKETLPVASEDMTILDLDQCVEDEDKEQMDLVIPCPCRETAEGNREGLV
LNSSEEKESQLVSCGDEKESEMLGPCRWFDGEMLLFDDIIQNELLDDEGALNEGREDDFSLI
RLEGEKKITGRNEDHGKQIVETGNPNNSRDELYRCSSIGSCSDEWSWENLVQWDENVDNM
VTCFWEGDNLGEGENQKLQEMDNAKQNAFVAWLLS
>Manes.11G020600
MGRSPCCAKEGLNRGAWTAMEDKILKDYIQIHGDGKWRSLPKKAGLKRCGKSCRLRWLN
YLRPGIKRGNITHDEEELIIRLHNLLGNRWSLIAGRLPGRTDNEIKNYWNTIIGKKLQNQECS
SSSRHDKQIKFKQSQRRIKPTIQTSISIVNADAKKIFWTKASKCSKVVIPIKTGSQNPVDDNT
VVPPPSMPNISGDLHRYWQFTSEDEDNNKNSSSSSDLMLDFEMDEELLSDLLNADFSLLNY
NLENGAASETNTVCEHPKLSLNSEKTLLLLDDEIQDSGFPSMDALIEFGEINWIQDLENKGA
AEEEKV
>Manes.16G085200
MGRSPCCSKEGLNRGAWTAMEDRILTAYVTANGEGKWRNLPKRAGLKRCGKSCRLRWLN
YLRPDIKRGNISHDEEELIIRLHKLLGNRWSLIAGRLPGRTDNEIKNYWNTTLRKKASAQST
SPQSNYSRQKRLATEPNSSPQPAKVIRTRATRCTKVLIPSQSPPLLPEFHPSQDLDSSPPLHCG
AVTNEEVDQDLDLLNFLDCRGFQDSHGDGALLDFQSKDLALEDPPMFKDLANTASLEDNA
NLDLDSLVYLLDSEEWPLH
>Manes.05G020800
MGRAPCCSKVGLHRGPWTPREDTLLIKYIQSRGEGNWRSLPKKAGLLRCGKSCRLRWMN
YLRPDIKRGNITPDEDDLIIRMHSLLGNRWSLIAGRLPGRTDNEIKNYWNTHLSKRLRSQGT
DPNTHKKLAETVQQQGKKKKKNNNNTSRNSKNKNNKAKVELEKAKVIHLPRPVRFSSLS
LLRNDSFECNTSLSSCQGGEGEGEGGLSAEAVTENPWCSFKYDHDDGISFLVGDVDHDIVN
GPDLGCVPASNANLEKLYEEYLQLLKTDDDHGKLDYSFAESLSL
>Manes.01G115400
MRKPCCDKQGNNNKGAWSKQEDQKLIDYITTHGQGCWRSIPQAAGLHRCGKSCRLRWR
NYLRPDIKRGNFAQDEEDLIIKLHALLGNRWSLIAGRLPGRTDNEVKNHWNSYLRKKLINM
GIDPNNHRLNQILPRSQPVDDDEEACKSNKSKADTDGVSDAATSSLEEDETSAGSYHINLDL
TIAVPSPAHAPNLETKPENCGIAATGEVLQNETSPALLLFL
>Manes.01G147500
MGRSPCCEKAQINKGAWTREEDERLVAYIQAHGQGSWRTLPKAAGLLRCGKSCRLRWINY
LRPDVKRGNFTVEEDELIIELHSLLGNKWSLIAGRLPGRTDNEIKNYWNTHMRRKLLSRGI
DPSNHRSTSELYVDSMSNSLGAVLKEENIAGGFRYKHQKNPYERKCQELNLDLKICPPFQH
QSEPLNREVLCFYCRLGLQKSKKCSCSDDLLHMKNGDGGETYSRELGYCLQGSM
>Manes.12G083100
MGRSPCCEKEHTNKGAWTKEEDERLINYIKLHGEGCWRSLPKAAGLLRCGKSCRLRWINY
LRPDLKRGNFTEEEDELIIKLHSLLGNKWSLIAARLPGRTDNEIKNYWNTHIKRKLYSRGID
PQTHRPVSSGAAAATDTFNATTATAALGKCRSSSTNHNKNNKSSINVSKIEIHNPLSQSIEA
RECINTNNMKICSSSNKKVGTDSSAEDSNCSSGVTTEEICPEINLDLSIGLPRQQPQVSSINFI

KQKQADNYQQQQYHHQQQQPQRQNLTHVFSEASAKSVCLCCSVGLQSNQTCSCRVMETS
LTAGHFSRYYRSLNF
>Manes.13G075100
MGRSPCCEKEHTNKGAWTKEEDERLINYIKLHGEGCWRSLPKAAGLLRCGKSCRLRWINY
LRPDLKRGNFTEEEDELIINLHSLLGNKWSLIAARLPGRTDNEIKNYWNTHIKRKLYSRGID
PQTHRSLNSTAAATTTTTTTATAPLSNSRSSSSNYNNNQNNKNNTNVSQIETQDSLAQFMK
APDCINMNICNSNIKIPTDSSAEESNCSSGVTTEEVCPEINLELYIGLPYQQKPQVSSNFKEKK
QANHHHHQQTQQQNLTYGCSGASAKDVCLCCNLGLQSNQTCSCRVIETSFTADSFSRYYK
PLNS
>Manes.18G103600
MGRSPCCEKAHTNKGAWTKEEDQRLIDYIRVHGEGCWRSLPKAAGLLRCGKSCRLRWIN
YLRPDLKRGNFTDEEDELIIKLHSLLGNKWSLIAGRLPGRTDNEIKNYWNTHIKRKLMNQG
IDPQTHRPLNEKTTTTTTTTTTTTTTTTTGAVKPSTNRVTQLNFENASPQSISEINLLKSNIDF
NYSNFSPIKTESVEENNCTSSGMTTDEEHHHRYHHHQERESSHENEEVNLELTIALAPTRNE
LTPRYSSNTADSKLQQQAPYQLLEKIVTGTVCTCCQLGSQRSEMCRNCQNSNGFYRYYYY
>Manes.02G194800
MGRSPCCEKAHTNKGAWTKEEDQRLIDYIRVHGEGCWRSLPKAAGLLRCGKSCRLRWIN
YLRPDLKRGNFTDEEDELIIKLHSLLGNKWSLIAGRLPGRTDNEIKNYWNTHIKRKLISRGI
DPQTHRPLNVKPTTTTTTTTTTTTTTTTTSAGAAKPTANRVTQLNFENASPQSKSEITLLKS
NIDFKYSNSFNPIKAESIEDNNCTSSGMTTEEEHHHLHHHRQEEERNSHENQEVNLELTIGL
PPMQSELTRTSSSNSADAESKLQQQTPYQFLSKMVTWGVCTCCQLGSQRSELCRNCQNSN
GFYRFYH
>Manes.04G074900
MGRSPCCEKAHTNKGAWTKEEDDRLIAYIRAHGEGCWRSLPKAAGLLRCGKSCRLRWIN
YLRPDLKRGNFTEEEDELIIKLHSLLGNKWSLIAGRLPGRTDNEIKNYWNTHIRRKLLNRGI
DPATHRPLNEPPQEASTTRTTTTISFNHVKEENEMISNTTPVVCKEEKNPVQEKCPDLNLEL
KISLPYQSQVPEPMKTGARDLCFACRLGLQNSKHCSCNLGAHIGSSSGSTNSGYEFLAMKS
GVLDYRSLEMK
>Manes.11G094800
MGRSPCCEKAHTNKGAWTKEEDDRLIAYIRAHGEGCWRSLPKAAGLLRCGKSCRLRWIN
YLRPDLKRGNFTEEEDELIIKLHSLLGNKWSLIAGRLPGRTDNEIKNYWNTHIRRKLLNRGI
DPATHRPLHEPAQESAVAATTTTTISFSHVKEEKDKIICTTAAVCKEEKNPIQERCPDLNLELR
ISLPYQSQQPEAMKTGARGLCFACRLGLQNSKDCSCNLGSSSSGSSNSGYDFLATKSGVLD
YRSLEMK
>Manes.05G205700
MGRIPCCEKDNVKRGQWTPEEDNKLSSYIAQHGTRNWRLIPKNAGLQRCGKSCRLRWTN
YLRPDLKHGQFSDAEEQTIVNLHSVVGNRWSLIAAQLPGRTDNDVKNHWNTKLKKKLSG
MGIDPVTHKTFSHLMAEIATTLAPPQVAHLAEAALGCFKDEMLHLLTKKRIDFQLQQPNSN
AAPGNTSLPYIVTKRDDNEDTIEKIKLGLSRAMQEPSMIPPNKTWESAGATSANFAGECSGF
PASITGFQCGPSSFGNEGAVSAWSQSMCTGSTFTAGDQQGQLHEKLEDENGEDSEGRKEIR
NGPSIFNTDCVVWDLPSDDLMNPIV
>Manes.14G072800
MGRPPCCDKLNVKRGLWTAEEDAKILAYVSKHGTGNWTALPKKAGLRRCGKSCRLRWTN
YLRPDLKHDSFTPQEEELIVRLHAAIGSRWSIIALQLPGRTDNDVKNYWNTKLRKKLSEMG

IDPVTHKPFSQILADYGNIGCLPKYGTRIGSLTRDLKNAFISKPAEPTEGIITNISNHLVPPKLE
PIHECFFNSKNTISTDANSNHSLDLLDQLQAIKLVTEVSSCSNCETISDHFFKEGSLSSLSSSSS
SSSSTCSTANQEKSAVNFSWRDFLLEDAFLPSDHPQEQENAMELSSKDLTNQAQNVIPQGQI
GCEVTVSERDNVGVERTELAIPSSSFQIPSSSSASFVEAMLHQGNKNFLDFPNLVEEPFSY
>Manes.15G175900
MGRPPCCDKSNVKRGLWTAEEDAKILAYVSNHGIGNWTLVPKKAGLNRCGKSCRLRWTN
YLRPDLKHDNFTPQEEELIINLHKAVGSRWSLIARQLPGRTDNDVKNYWNTKLRKKLTEM
GIDPITHKPFSQILSDYGNISGLSNPRNQIASFNKNLNSNLITKPEPSFVLTSSNNVILKQENS
WELLPQFQATSHELVQPHLFNEVSSSSSSSSSTSVAQSQPPLTPSSPSLWSEFLLGDPLVYVDF
QQQQQQKQDSLGAISSTSKQIDMLFQGKFASGNEDFGWYDQRGIYGDASSSSASSFVDGIL
DKDREMGSQFPEILDPFF
>Manes.14G066200
MEGGNTTTTTEYRKGLWTVEEDRILIDYIRVHGKGKWNRVARVTGLKRCGKSCRLRWMN
YLSPGVKRDNFSEEEDDLIIRLHKLLGNRWSLIAGRVPGRTDNQVKNYWNTHLSKRLGVK
NGKFKASAPSPGFSTKELREDFNASSSAETATNPACTNGVVADHDAMENGSKSTAMELTSN
QQRMPAGEWDSNPFLFLNDIDPNLYAPQFMEFLDESLDFVWHDF
>Manes.01G090700
MGRPPCCDKLGVKKGPWTPEEDIMLVSYIQEHGPGNWRAVPNNTGLLRCSKSCRLRWTN
YLRPGIKRGNFTDHEEKMIIHLQALLGNRWAAIASYLPERTDNDIKNYWNTHLKKKLKKL
QTTGHEGDSKNDILSSSTVSQPITRGQWERRLQTDIHTARQALYEALSPEKKSSLLLPEFKRS
NGGHSCAKPASTYASSTENIAKLLKGWMRNGPNKQAQTKSSATTKNSFNSIARTTDSICSE
GIPSKADKNGTELAQAFELLFGFESFDYPNSDFSHTDEANLFQVETKPNSSAQILPPLSLLEK
WLLDEGTLQGKDYLSEVSVDENNIF
>Manes.02G046100
MGRSPCCNKLGMKKGPWTPEEDITLVSYIQEHGPGNWRAVPTNTGLLRCSKSCRLRWTNY
LRPGIKRGNFTDHEEKMIIHLQALLGNRWAAIASYLPQRTDNDIKNYWNTHLKKKLKKLET
TSHAGHSKNELTSSTVSQPISRGQWERRLQTDIHTAKQALYQALSPEKQSNLLTELQQSGGE
HSYAKPASTYASSTENIAKLLKGWLRKTPKQTQTNLSATTQNSFNSITGTDSICSEGIPSKAE
KNGIELEEAFQSLFGFDKSFEHSSNSDFSQTMSPDEASLFQDESKPNSSSQMPPFPLLERWLF
NEEATQWKDYLGEVTLDESNLFFGTCF
>Manes.06G092600
MGRPPCCDKVGVKKGPWTPEEDIILVSYIQEHGPGNWRSVPTNTGLLRCSKSCRLRWTNY
LRPGIKRGNFTEHEEKMIIHLQALLGNRWAAMASYLPQRTDNDIKNYWNTHLKKKLKKLQ
DGKNDDGFSSASQQCKGQWERRLQTDIRLAKKALYEALSLDKSSSLTDTKPSTTNVSHPST
RPHQVEGSAYASSAENIARLLENWMKKSPKPAQTNSDTTQTSFNNAVATNSSSSEGAHSETA
LDHHGFDSLFSFNSSNSPDASQSVDENANLTPETSLFQDESKPNMENQVPFSLLEKWLFDE
GGAQGPEDLINMCLEDNTVGDF
>Manes.14G077700
MGRPPCCDKVGVKKGPWTPEEDIILVSYIQEHGPGNWRSVPTNTGLLRCSKSCRLRWTNY
LRPGIKRGNFTDHEEKMIIHLQALLGNRWAAIASYLPQRTDNDIKNYWNTHLKKKLKKLQ
DGQNHGGFSASQPKGQWERRLQTDIRLAKQALCEALSLDKSSSLTDSKPSTTNVTRPHHVF
GPTYASSAENIARLLENWKKNSPKQAQTNSDTAANSSCSETTPDHGFEHSLFSFNSSNSSDA
SQSVDENVKLTPETSLFHEESKPNIENQVPFSLLEKWLLDEGCSQSHEDLINMSIDDSSVGLF
>Manes.08G151100

MGRPPCCDKIGIKKGPWTPEEDIILVSYIQENGPGNWRSVPTNTGLLRCSKSCRLRWTNYLR
PGIKRGNFTPHEEGMIIHLQALLGNKWAAIASYLPQRTDNDIKNYWNTHLKKKLKKSQSAL
DHNPMASQDSTTSTTHRFVSKGLFSERSRSLNLSPNSSSDLRLNQLTSSTYASSTENISRLLE
GWMRSSPRPDNHGINDPWNKAEGSIENSVAATSLQCYRPKDELETGGGGGLISHEEFESILS
FDQNLNNVAWDKSTCDSNTVSTVKVCRKNSENEEKEHEIITVAEKKQKSESNPPFSFLENW
LWDETATAQVEELSPIF
>Manes.09G135700
MGRPPCCDKVGIKKGPWTPEEDIILVSYIQEHGPGNWRSVPTNTGLLRCSKSCRLRWTNYL
RPGIKRGNFTPHEEGMIIHLQALLGNKWAAIASYLPQRTDNDIKNYWNTHLKKKLKKFQA
LDHPIAAHDSSSTTTTAANNQFVSKSFYERSRILNSSPNSSTLRLNQNSSTYASSTENISRLLE
GWMRSSPKPGNNHATNDLFKEKWNQNDNNLENPDGSIGISAATTSLQCYRPKADQEHGG
GSGNLISHEEFESILSFENLNTVAWDKSTRDFTNNFCAVNGIIHQDSASEEKENDTIAAEIRK
QKSDSNPPLSFLEKWLLDETTATVQVEESMELSPIF
>Manes.01G057200
MGHHSCCNQQKVKRGLWSPEEDEKLIRYITTHGYGCWSEVPEKAGLQRCGKSCRLRWIN
YLRPDIRRGRFTPEEEKLIINLHGVVGNRWAHIASHLPGRTDNEIKNYWNSWIKKKIRKPSA
STTTVAATSSGIDHSHINYGSNQIDLVHQDLTTRPHVQETLFSSPAPLFMFDTGTTPLDGIAD
NSVRGEFFNEPASLNTETWNTNQHHHHQVQILPPQATFSIGMDTNYLPPLIENMENMVPIE
MQSCSMDDEGEIALGCLQRQELNEWVDSQQCSNFLFWDNVDGPLGGEEIAPNSSSMGAA
LSSFPSSL
>Manes.02G017300
MGHHSCCNQQKVKRGLWSPEEDEKLIRYITTHGYGCWSEVPDKAGLQRCGKSCRLRWIN
YLRPDIRRGRFTPEEEKLIINLHGVVGNRWAHIASHLPGRTDNEIKNYWNSWIKKKIRKPSA
SPTTVAATSRGTEHSSNQIDLMYQDLTTRPQVQETLFSTPAPLFMFDTGTSPFDATAHNSVR
GELFNDPASLTAETWNSNQHHHHHQVQAFPHHQATFSIGIDTNNYLPPLIDNMIPMDEEGDI
ALKCMQRQELNSNFLFWDNVDEPLGGEEIAAISSSMEASLSSFPSSL
>Manes.04G140800
MGHHSCCNKQKVKRGLWSPEEDEKLINYISTYGHGCWSSIPKHAGLQRCGKSCRLRWINY
LRPDLKRGSFSPQEAALIVELHSILGNRWAQIAKHLPGRTDNEVKNFWNSSIKKKLICHDVP
GLASFTDVHNPNGGSEEAFFSLTGNPNLILSTAQQDQIYLPSPASMLQNFGQGDFKFNQPNN
YNLDLAHFTSSIVPPPLNNSTTSFDPMWALPYLTHHLDPNHQEDQILSNGAGTHYIGDHKFI
QDQNIPIMPHYENQGMVPMMPKLCEIIEGSVCCNIQSSSSSSAGSQEVLIDPVARLPCFPSGS
YNPQEPLVPTNQMEYIDAIMSSLPSSSSSSSLSAFSSGQFGANPNNNINNLPSCCSWDA
>Manes.11G024700
MGHHSCCNKQKVKRGLWSPEEDEKLINYISTYGHGCWSSVPKLAGLQRCGKSCRLRWIN
YLRPDLKRGSFSPQEAALIVELHSILGNRWAQIAKHLPGRTDNEVKNFWNSSIKKKLISHDV
HSLASFTDVHNPNVSEEAFFSLTGNPNLILTAQQDQLYLPSPASMLQSFGQGDFKFNQPNNY
NLDFAHFAPPTIPPAQLNNSSSSFDPVWTLPYPPQHIGTNHQEDQILSNGAGPHYIGSKANQ
DETLAILPNYENQTMVPMMPKLCDIIESSACCNIPSSSSSSSTLQEVLIDPVSRLPCFPAGSYP
HEPHVPANIQMEYIDAIMSSLPSSSSSSSLSAFSSSQFGANPNNNNNNNNNNKLPSSCSWDA
>Manes.02G005200
MGRAPCCSKVGLHRGQWTPREDKLLINYIRAHGEGHWRSLPKKADIKRGNITPDEDDLIIR
LHSLLGNRWSLIAGRLPGRTDNEIKNYWNSHLSKRLNKTSGTRSSSSESKKKNKEGNKDG
ANGKAAKSKIDLPKAIRVTSVSITRTSSTSSLRGSSKTHAEVHNLPWLEMVCPNLEVRDGII

NDGETLPCNEDHDDLDMPTNENMLDEMFEEYEQLLRTDNYAQLDSFIDSLLA
>Manes.15G149600
MEKKPCNSLGNEVRKGPWTMEEDFILINHIANHGESVWNSLARAAGLKRTGKSCRLRWL
NYLRPDVRRGNITPEEQLLIMELHAKWGNKWSKIAKHLPGRTDNEIKNYWRTRIQKYIKQE
ETFAGQSCEIKEHASTSLQVSGMIDTYSPQSYQETLETFPGATLAPQSTQACWSFEDVWSTH
FA
>Manes.17G113200
MDKKPCISEEAEVRKGPWTMEEDMILINYIANHGEGAWNSLAKAAGLKRTGKSCRLRWL
NYLRPDVRRGNITPEEQLLIMELHAKWGNRWSKIAKHLPGRTDNEIKNFWRTRIQKHIKQE
EAFSGQSSCEINEHPSTSLQASAMMETYSPSSYQESTMEAFPAETLAPDQSTDNYWSMEDL
WSMQLFNGDYLINSRVN
>Manes.14G026300
MGCRSSDKPKPKHRKGLWSPEEDQKLRNYVLKHGHGCWSSVPINAGLRRNGKSCRLRWI
NYLRPGLKRGTFSLQEEETILNLHRLLGNKWSQMAQHLPGRTDNEIKNYWHSHLKKKIHK
ADHGVLEEPNAHDTSSDNLESNNRDQSVVQLSDHSPKKAYRSSLPKLMFAEWLSLDSFSSL
NEPIMGSLDQNNSRFQDNLMQGCVLNEGTFCNSEFHNSISDASAEEMFSSQFRFEWESSGN
DQFVDFGSGDDICSEFTVNNDLMFIQNYKH
>Manes.16G076800
MGRAPCCDKANVKRGPWSPEEDATLKRYLETYGTGGNWIALPQKAGLKRCGKSCRLRWL
NYLRPDIKHGGFTEEEDKIIFTLYSQMGSRWSLIASHLPGRTDNDVKNYWNTKLKKKLLLG
GNPSLAIKNNTEIITPADHNYNATTAPSATSSLPNVIPRTETTCSFTFWDSLTHSSVTLPILSDV
VYDQHLLDPSQILSLDPVDQFSFTPGIMDNLSELGSNLINNHNIVSSSQEGSSISDSSSIVMDH
NLSMPAGILMDSGFGFPYDPVTALLFEDKAGEVASSGYAEIKH
>Manes.16G106000
MGRAPCCDKANVKRGPWSAEEDATLKNYLQKHGTGGNWISLPRKAGLKRCGKSCRLRW
LNYLRPDIKHGGFTEEEDDLICSLYSSMGSRWSVIASRLPGRTDNDVKNYWNTKLKKKML
EGKLDAKKSSKIRNKIDISINNCNDPVVQFSAFSVPESETYNPGNSSPCFSAYSTTLPPLMEM
GSFQQCCDPQFQGLILNQTQFPISSFMEVPSFGTSGCDSYSVSSSHQEASSLVPSDSNRYSISS
GNGSLVKDNGFEFLYDELLNDLGFQGKFSPEVAPFLGNN
>Manes.13G107900
MGRAPCCDKANVKKGPWSPEEDAKLKAYIEQNGTGGNWIALPQKIGLKRCGKSCRLRWL
NYLRPNLKHGSFSEEEDNIICSLYISIGSRWSIIAAQLPGRTDNDIKNYWNTRLKKKLLGKQR
KEQAARRATLRQEIKSESQSFMAPSGVVLNQQTPNYYWPELPSSAIMPAMNPSQDSHFCDQ
ESLKSLLIKLGGRFSDDHQESNMASTVYPLDGSCISTQDQPYSSSMNMLSSSGTSIASTESPC
SQLPNTNYAVSGAAGPSTYQGLDRFPVELHELMYGNQQQLESLESLYGIDNGANGVMSAG
ETTSWGNVSSLAYPQLVSELETCLQNQPQDHSSFEVSSYFGPK
>Manes.18G051300
MGRAPCCDKANVKKGPWSPEEDAKLKAYIEKHGTGGNWIALPQKIGLKRCGKSCRLRWL
NYLRPNIKHGGFSEEEDNIICSLYISIGSRWSIIAAQLPGRTDNDIKNYWNTRLKKKLLGKQR
KDHQARRASGIKQEMKRGNANPMVSADNKNNQNPYWPELPLLAPIPFSNQEPRFNDHASI
RKLLIKLGGRFSEDDQLIRNATSTQFPNGVSYAQQLYDQPINNVSSSASMDTSNDTAVQFAQ
AHYNIEGARLQMVQGESNFPAVGIEEMAYNNPQRLDGLEFLLSDDMLNDRIGTTSGESVV
GSMVEMSSLVYPPMASNCEGIQQGLLQECSLEELRYPGIL
>Manes.06G024800

MGRAPCCDKANVKKGPWSPEEDAKLKAYIEEYGTGGNWIALPQKIGLKRCGKSCRLRWL
NYLRPNIKHGGFSEEEDNIICSLYISIGSRWSIIAAQLPGRTDNDIKNYWNTRLKKKLLGRRK
QSNINRLSSETSDSNRVEESSSSMALSQSALERLQLHIQLQSLQNPFSFYNNPALWPKLHPFQ
EKMLLQSMNESSNTLMQQFLNSPHGNGQKLDIYEQLCGSDTLDQDYLKFDNPKVDCLEN
SLKGLASTDSSIPFVNGNNVADSSMGVRVDAVEQSDAANQPVSTFQPELENFISNKTSGFVS
QQDQFAEFDCFKEMNASKDSLIWWSNEFETKSASSTSWESTSALQTEGMFQDYELSYNM
>Manes.11G151100
MGRAPCCDKANVKKGPWSPEEDSKLKEYIEKFGTGGNWIALPQKAGLKRCGKSCRLRWL
NYLRPNIKHGEFSDEEDRIICTLFANIGSRWSIIAAQLPGRTDNDIKNYWNTKLKKKLMAVA
PQSQTKPFSFPSPHHQSSPLSSHSLLSLYKDSSSSSFPYFPPNFKPFTTVFDPISPIPSNLLAKNT
TTTATNSSSLFQTQESLLNPLHYYHPVKDSNNGSSSNNLLIFGSEASCSSNSDGSCSQISYGG
REIKQEDMGFQSYISNGYEENQKFMFSYGINSCNGSENLNQWTEKTTGYFGETSPFEYDLE
DVKQLISSSSNNSCNNNNNILLDENKTQEKYMYYY
>Manes.04G014800
MGRAPCCDKANVKKGPWSPEEDSKLKDYIEKFGTGGNWIALPHKAGLKRCGKSCRLRWL
NYLRPNIKHGEFSDEEDRIICTLFASIGSRWSIIAAQLPGRTDNDIKNYWNTKLKKKLMAMA
SHQSQRKSVPFSSPPNHQSPPLSYNSLSSLYKDSSADFSICFDPISPIPSNLLATNSSFLFQTQEP
ALLNPLQYYHPLKDNNNNCSSSSSSGNNNLLVFGSEASCSSNSDGSCSKISYGGSKEIIKQEE
MGFQGFISNGYEENQKFMLSGDLNQWTEKVTGYFGETLALDYCLEDVKQLISSSSSSNNSS
NNYYFLTDENKTREKFMYYYY
>Manes.08G011300
MGRAPCCDKANVKKGPWSPEEDTKLKDYIEKHGTGGNWISLPQRAGLKRCGKSCRLRWL
NYLRPNIKHGDFSDDEDRIICSLYANIGSRWSIIAAQLPGRTDNDIKNYWNTKLKKKLMGM
MIHPSQAKLPHQLPASFSSLLHQVSSSLSSPSPSTAISSSSAPSYTPARSFAEPVPFSSNNNSFTT
AASIFSPQDSSFLAAIQNYQMKDSCSSSDGSCNNHISHDKDLEYEYGGGGASATEQMGLQN
YFYYGVEESQKLLDGGGENPIIDYGLEEIKQLISSSSSCSNNFLFEENKTSEGRIMYY
>Manes.09G065400
MGRAPCCDKANVKKGPWSPEEDAKLKDYIEKQGTVGNWIALPQKAGLKRCGKSCRLRW
LNYLRPNIKHGDFSDDEDKIICKLYSNIGSRWSIIAAQLPGRTDNDIKNYWNTKLKKKLMG
MMMIHPSQTKLPHQLTTKFASLLCQASSSSSSIPSSPSTAISSPSSSYALARSFTEPIPFSSNNSF
TAANKSILPSQESSFVAALENYQMKDSSALLMCGGETSCSSSDGSCNNQITHEYGGGASSA
EQMGLQSYFYNGVEESQKLVGDGGWSEKQNGLWGENPIIDYGLEEIKLLISTSSCNNFLFE
ENKTAEESVMYY
>Manes.04G144400
MTAKREHKPIEAMTNMPTKKEISKGAWAPEEDKKLAEVIAIHGAKRWKIIAEKAGLNRCG
KSCRLRWLNYLRPNIKRGNISDQEEDLIIRLHKLLGNRWSLIAGRLPGRTDNEVKNYWNSH
LCKKINQKEKQSGASIGEESKGEKRTTEKADTVEVTREEKQSSCNNYTGGEESNTSFNVDD
FFDFSNEDRLNLEWMSPFLEMDGRFTGMS
>Manes.10G019700
MQNPTDDDVMITEDEAKTPFADSPTAGDSEVGGAAESVELVGPAGGGSGGVGASLNSRVK
GPWSPEEDAVLTQLVSKFGARNWSLIARGIPGRSGKSCRLRWCNQLDPCLKRKPFTDEEDH
IIISAHAIHGNKWAAIARLLPGRTDNAIKNHWNSTLRRRCMDQGRFKPGRSDAMEDGSHD
KTKASSEETLSVGDVNSFKLPEGRDVTIDDQPDQEEDKAQADKVPQTNGFDFAAETQVHP
TLPRPKARISAFSVYTPPSGPKTGYSRTVPTHGPLVQSSKTDFTACKFLEDIHVDPIVPLQCG

YGCCTTPSTGHPQSSLLGPEFVEYEELPAFSSQELISIATDLNNIAWIKSGLENSSNGITGNTA
NYKISQGAVVGSQMGVPEQNLRNGHMPYEEGRNKLMGMMTDNLSTSACTAFCNAS
>Manes.01G247600
MDRRRRKQHKRCSEEVCSIEWEFINMSDQEEDLIYRMYKLVGDRWALIAGRIPGRKAEEIE
RFWIMRHGEVFASRRKEIKLSNS
>Manes.05G024900
MDRRRKKQSKAATPRSEEVSSIEWEFINMSEQEEDLIYRMYKLVGDRWALIAGRIPGRKAE
EIERFWIMRHGEGFAGRRKELKKSKC
>Manes.06G157500
MCSETMDQKQTEATISESEEVSSIEWEFINMSEQEEDLIYRMYRLVGERWDLIAGRLPGRKP
EEIERFWIMRHHEAFAKKRKTA
>Manes.08G077900
MSEVKIEECCLENKQLTAASSSSISEGSGSAILKSPGVSSPATASPTHRRTTGPIRRAKGGWTP
EEDETLRNAVAAFKGKSWKKIAEFFPDRSEVQCLHRWQKVLNPDLVKGPWTQEEDDKITE
LVAKYGPTKWSVISKSLPGRIGKQCRERWHNHLNPDIKKDAWTLEEELALMNAHRIHGNK
WAEIAKVLPGRTDNSIKNHWNSSLKKKLDFYLATGELPPVVKSDIVFPSRKLPSVAKNGIQN
GTRDTNKPAVTKTSKESDSTAQTSSGTTDACKLEEDGKDQLESLALVRDMAASSSVFPNES
ADSEGVECTHSFVADLSCCDSEALPKCENYGINYETNEEKVFGSQLQFETSTYGSLYYEPPQ
LETCTPINLDPSVMHQVQHEYSSSPISSPISFFTPPCVKSSNLRAQSPESILRIAAKSYPNTPSIF
RKRKTGSQVHLLPSKIGKVGQESIEDRLQRRTEYTPEKAGSQKGNLHDSPHPDDSTSLPNG
KAFNASPPYRLKSKRTAVFKSVERQLEFTFDKERCDTTKSVNGSSLITEDCARATKMGVT
>Manes.12G155900
MENGRGSSSDSGKSCHRGHWRPAEDEKLRQLVNQYGPQNWNFIAEHLQGRSGKSCRLRW
YNQLDPNINKKPFTEEEEERLLKAHQIQGNRWASIARLFPGRTDNAVKNHYHVVMARRKR
ERLSSVYGKRSFHLHPTESNKKITTPGSNFESYYRLHQPLDHHSKLGFQSNGYFTMCSSSSP
SWTISASTITNESLNFDFLDGKGKGYMNSSSSSSHTKDGSHGFNGSMYGGYYQSLFGSSAQ
ISNSTKVAANNNNPLANLLSYGGGNHASKSATLQKELEDGAINQKDVSFIDFLGVGIS
>Manes.14G002400
MRKPEVCGKNNNKLRKGLWSPEEDDKLMNYMLNNGQGCWSDVARNAGLQRCGKSCRL
RWINYLRPDLKRGAFSPQEEELIIHLHSLLGNRWSQIAAGLPGRTDNEIKNFWNSTIKKRLK
NLTSSAASPNTSDSYSEPSKEVAAAAAAAIVGGGFISMQEQAMMNPMCTDPSLSSASSSNT
SMQAMFLNQMDSSPTLDHGLSMYGANAYFNNNAPPCMTPIGITSGDDLHGNQGILGGVNI
GIGGELHIPPLESIIIEENARTEDIATHGNTTNNYPFSNVNKMNSNCNKAENMAAGIGNLWQ
GEDLKVGDWDLEELMKDVSSFPFLDFSN
>Manes.02G041300
MAPVNSEESSNSKMVMNKGAWTAEEDKILAEYIEVHGAKRWKAVAMKAGLKRCGKSCR
LRWLNYLRPNIKRGNISDEEEDLILRLHKLLGNRWSLIAGRLPGRTDNEIKNYWNSHLSKKI
NKMERTPESSIPQESIPDNAAAAAQDMMEEGSQGAVFPELSFDADGFFDFSMEGSCSLEWV
NKFLELDEDPWLADKS
>Manes.01G083100
MAPCKNSEASANCKMVMNKGAWTVEEDRKLADYIEVHGAKRWKTIAFKAGLKRCGKSC
RLRWLNYLRPNIKRGNISDEEEDLILRLHKLLGNRWSLIAGRLPGRTDNEIKNYWNSHLSK
KINKKEKASDESPSTQPQKSITEKTCDAVAAVEEMVEEGSKEEAIPERSFDVNQFLDFSMEG
TYDLEWLNKFLQLDEDAWLAADN

>Manes.06G164800
MRKPEACGKNNNNNNKLRKGLWSPEEDDKLMNYMLNNGQGCWSDVARNAGLQRCGKS
CRLRWINYLRPDLKRGAFSPQEEELIIHLHSLLGNRWSQIAARLPGRTDNEIKNFWNSTIKK
RLKNLSSTASPNTSDSSSEPSKEATAASIGGGFISMQEQGMTPMYIYPSLSSSSSSNTSMQAM
FLNQMMDPLPTFDHGLSMYGASVYFNNDASPCMTQIGVSGDHDFYGNQGILGSVNIGIEG
ELHIPPLESISIEENTKTEDMYDTNNNSKDPYSHVNRNNSICSNNNKAQNMAAGVGNLWQ
AGEELKVGDWDLEDLMKDVPSFPFLDFSS
>Manes.08G145500
MKNPSSPISRKTTTTTTTPCCTKVGIKRGPWTPEEDELLSSYVKKEGTGRWRTLPSRAGLLR
CGKSCRLRWMNYLRPSVKRGRIAPDEEDIILRLHRLLGNRWSLIAGRIPGRTDNEIKNYWN
THLSKKLISQGIDPRTHKPLNPDHNSSQFAKDPNQNSGPKSVNLEETGRTYRVMATKVSQTF
NMTNLDHYQNPEVVEDGNDNRLNDNCDALVMELQSDQGHSNEEYHFNGNEDEDTFSSFL
ESLINENENLFLINQQQQQQHPNMIAPPVQHGVFSAQPFNNTATWEVEVAPSMAALGDE
MLL
>Manes.09G139900
MRNPSSSSSPSARKTTATPCCSKVGIKRGPWTPEEDELLSNYIKKEGAERWRTLPKRAGLLR
CGKSCRLRWMNYLRPSVKRGRIAPDEEDLILRLHRLLGNRWSLIAGRIPGRTDNEIKNYWN
THLSKKLISQGIDPRTHKPLNPNPNPSQRAQDPNQNSGLKSVHLEETGRTYRTIATKVSQNF
NMTNPDGYRNPIVDEGGNNNWLNFNGLVMGLQSDQGHNNAEYNYIANENEDPFSSFLDA
LIDENENLFTINQQNHHQQQHLNNMAAPSVQVQPFVSSAQTFNNTSIWEAEVAPSMAVL
GEEDVGLT
>Manes.16G007100
MSMADSVDYTSNETTSTQDSKEGVKSQDSRPEFSEDEESLIARMFSLVGERWSLIAGRIPGR
TAEEIEKYWTSKYSSSSER
>Manes.14G104200
MHSIRPPVRAAASVTKRMCNSTEEDSQLRRGPWTLEEDTLITHYIACHGEGRWNMLAKHA
GLKRTGKSCRLRWLNYLKPDIKRGNLTPQEQLLILELHSKWGNRWSKIAQQLPGRTDNEIK
NYWRTRVQRQARQLNIESNSKRFLDAVRCFWMPRLLQKVEQNCYSYSTLSTMDSQTDAA
ASASSTNFTVDNSLSSESFPIQTKWANYSNLPSEHSNSVTSPSVLSTDSKPISPQTQTLENPAS
ISPPVLDNTVYDNLIVGDCYYVENSGYDMDGLNTASVPEIVAFGDSTSECQMAESNWVFD
NDMADTLWNMDDTWQFRL
>Manes.05G037100
MGRTPCCDKKGLKKGPWTPEEDEILISYIKKNGHGSWRSLPKLAGLLRCGKSCRLRWTNY
LRPDIKRGPFTLEEEKLVIQLHGILGNRWAAIASQLPGRTDNEIKNLWNTHLKKRLLCMGLD
PQTHEPFTSCGPAIKAPASPATRHMAQWESARLEAEARLSRESSLFNPPTPGKSDNDYFLRI
WNSEVGDSFRKFNQADKTTRKSPASASQASSSTKCGSVSAVTADISPNLAGCSTTARNQNE
DTECKSCKSYAEDLNAGSDSSSSLELEDSSDSTLQLLLDFPINNDMSFLEENMDGYATYSA
M
>Manes.06G105800
MSDYFSDKPSMKYGSPFEAPPSSPSKGFSQDLHHFDQFHANGLSLNPIFGAQNASNFDFFD
ALPYGSSTNIDFSDYECKPFADNNGGHGQVMDNFQNGGFLNLPHNQSNSIGTMMGSNQG
QGNMSLGFQEMKPINFVVPDEVSCINANQEYHKKVGVDKNRASSSMGRTWRGRKKNNV
VKGQWTIEEDRLLTQLVEQYGVRKWSHIAQMLPGRIGKQCRERWHNHLRPDIKKDMWSE
DEDKILIHAHAEIGNKWAEIAKRLPGRTENSIKNHWNATKRRQYSKRRCRSKYPKGSLLQE

YIKSLNLGSVPAAAGRFLGKSSDARAVNNISMKAPNLQPQVSEISQNNNRLFPNYYDFNEV
PAFDFDERMIQEGCSFGSFLDEMANGDRGFDEKSFEMNVAMEEVSPFMDFGEKKELDLVE
MISQARM
>Manes.15G149100
MELDRKLREEFPYLSSLLSDFPLKHEIESGFSSHEAPFSSNKGLFQSIHHLDHHNLHLPSPFN
SQYHLDHFTIEGSSKNPFLGVSATCIDPLEPLPNGFSSDLNAFVSAALLPANGGESGYDHRPL
HGSLQRRSFGDYNPQKFDEANDPLGQKLTYHHQSLNMRSMNLAKLPDEVSCITGDNGYG
KEADHRKDQRFQIKKDGKVHKKAQIIKGQWTPQEDRMLVHLVKQNGVKKWSQIAKMME
GRVGKQCRERWHNHLRPDIKKDAWTEEEDEILIEAHKEIGNRWAEIAKKLPGRTENTIKNH
WNATKRRQFTRRKGKEANSKPTILQCYIKNLTSSSATNHHQENNNPQDYLHKETSVSSADH
HHHLLKFPSSSLMHCDHNEGPNKFCVDTNFLFNDSYGFASSSLEEIPCTSVVDESNLEYEISL
ELYSLMKGAAAPAKEEMDLLEMITQ
>Manes.01G271600
MEGKRDEIRKGPWKAEEDEVLRNHVEKYGARDWSSIRSKGLLQRTGKSCRLRWVNKLRP
NLKNGCKFSVEEERVVIDLQTQFGNKWAKIATYLPGRTDNDVKNFWSSRQKRLARILQTSA
TPSSSSSSNSKPRKPKKQVPLLDVPTLQAPLFNFSMEEESSAKAQSWSTSYIETPEPISMVPV
QFHIDIVNNELSSYDANLVLVECQKEDQDPFPQISEYQPDLTFSPESQELLARLEDPELFNVF
GAVDAPELEPQLSLGLPLFDPIPSCMNGAREGRNPASRSTFFDDFPSDVFDNIVPLPSP
>Manes.05G052300
MEAKRDEIRKGPWKAEEDEVLINHVQRYGARDWSSIRSKGLLQRTGKSCRLRWVNKLRP
NLKNGCKFSMEEERVVIELQAQFGNKWAKIATYLPGRTDNDVKNFWSSRQKRLARILQTS
ATPSSSSSSNFKPRKAKKEVSVFHEVPTHQAPVFSFSMEEESSTKAQSCSSSFIETTEPVSTVP
LPFHADLVKTELPSYEANLAQMEAQTQIPFPEISQNQPDLTFSPESQELLARLDDPFLFNMFG
AVDAPELGAQPSLGPPLFGPVSSCINGGREARSPTTHGTFFDDFPSDVFDNIEPLPSP
>Manes.11G134300
MKMVQNEIRKGPWTEQEDILLINFVHLFGDRRWDSIAKVSGLNRTGKSCRLRWVNYLHPG
LKRGKMTPQEEKLVLELHAKWGNRWSRIARKLPGRTDNEIKNYWRTHMRKKAQERKRT
MSPSLSSFNSSSTSNITTQNSSPFPETAEASFYDTGGPESSSSGGQISEAEQEGEKGYSMDDI
WKDIENSIEPAYDGFSEEGCNFSSFSIASPSWEYCSDTLWRMDEEESKMFLPYECGTVFLTG
>Manes.01G065000
MVKVKKEELYDVDFADQIIDAPPCSVYYGAGDAAFRRRATGPTRQSTKGHWTEEEDYILT
ESVRRFHGKKWRKIAECLPQRTVSQCFTRWNRVLNPAIVKGTWTKEEDDSIIKSVRKHGPR
KWSLIAKSLPGRLGKQCRERWYNHLDPAIRKSSWTEEEELTLTYYHGIYGNKWAEIARFLP
GRTDNAIKNHWNCIVKKKLDSDSPACAEDLHKVASLNFCSCAMKTESKEVKEERQNPDEL
VSVHGRMGLKCSADTGASKLLCRLVNGEQNQLEAKHGATFKTSVGMDELMNGIHFSDVG
ANGSVAMIESGRNNSKHDILNSSMELRFDVSASTRPCCSALDAAVPFTLNSTKSPNRLRGHE
FCILHSEFGNKTESSLSYTSTSATGEDKSVTEKEGKFPKLNVHMQSVDSNEQRLHHESAHP
KDLVTDLDGAQPSIIHHHVLHANSPFSCSTPSNCARYAFVSVNSPESILRNSARTFKNTPSIIR
KRAYKEAGVDKSSDVDKSSDVASTPSWKISCTNSTSEDINNADIPNGKQGCLSFFCKPGSSL
AVKSLRRQLDYAFEMERDAAAAKCGNPFPATASPNIEFFSNAMVIP
>Manes.11G009900
MRHTKNESDDGVLSKDQTESLLAEEGSCGGSANGLMLKKGPWTSAEDEILIEYVKKHGEG
NWNAVQKHSGLFRCGKSCRLRWANHLRPNLKKGSFTQEEEQLIIELHAKMGNKWARMAA
HLPGRTDNEIKNYWNTRIKRRQRAGLPLYPPEVSLQASYESQLSLNISGIYSGDKDHQDLLQ

TNSYEIPEVIFDSLKANHGISSYVPELPVITASSMLMRGLSSSQYSNFMSPTVHCQKHLREST
TLIPGYSGSGKTEFTLSDQFQHNSCDRVAQSFGLSFPFHLDPTNKNPESFGGNQVSHILANG
NFSASKPNYGFVKMELPSFQYPENDLGSWGISPPRLLEIIDTLNQSTPIGTVESSPHNSGLLD
ALLHESETLSSTKNHSSDKSSNSSIVTHGELAESSALNICNTEWEEYGDTLSSLGHTTTSLFG
ECTPISTCGSSMDEPSATETLTGSNVKLTAVDQARSSEREKETTTRLDITHPDALLASDWLED
GSGYVEDQDFMLDNIAPLLSDDFSSDFEQMSTGASTSNQGWGLGSCAWNSMPAVCQMSEL
T
>Manes.17G023900
MYLQQRLQLSSPLLHNPFSFCFLPMGRPPCCDKSNVKRGLWTAEEDAKILAYVSSHGIGNW
TLVPKKAGLNRCGKSCRLRWTNYLRPDLKHDDFTPQEEQLIINLHKAVGSRWSLIARQLPG
RTDNDVKNYWNTKLKKKLSKMGIDPITHKSFSQILSEYGNIRGISNNNGNHVASFNKNFNS
TLVSKPEQPSSSVLTGPSSSNVILKPPIEQVHENSFINNSHSWEFLPQFQVTNHDILKPYLFNE
VSSSSSSSCSSTATQLNSPQSYTCQESQPPLTPSSSCFWSEFLVNDPVISADFHHLQQQQHQ
DSHGVLSSTSISTQNNIFHGKFTSGNGDFGPYDHGVMNGNQTNSYADASSSSASSFVDGIL
DKDREMGSQFPQLLDPSFDY
>Manes.03G131000
MMSASNGGSEGGGGGGDDDFAAAAATNQGAGRDMHANVHHDGSGKGGNGNGDGNGE
THLKRGPWTAEEDAILAEYVRKHGEGNWNAVQKHSGLSRCGKSCRLRWANHLRPNLKKG
AFSLEEERLIAGFHAKWGNKWARMASLLPGRTDNEIKNYWNTRVKRHQRRGIPLYPPDIQP
QHPSSPHFHHHRSSSSCITPTTPTSSFTFPTPHPLAPTSATPPHLSLTTPSTSFPTLPLFDFSQPP
HHHHTSHSTPTTPTSSFSFHTQLPSPSHAHIQNAASSVSPLSSPSTNASTNFPTLPLFDFSIPRT
LPVLQTPMRFKRFSSSPNMATVTTTN
>Manes.15G070500
MISTINGGSEGAGFSGSIGGGVDVAAAAAIHQGGGDMHANVHHGVNGTGSCSGGNDGNG
ETHLKKGPWTAEEDAILAEYVRKHGEGNWNAVQRHSGLSRCGKSCRLRWANHLRPNLKK
GAFSPEEERLIVELHAKFGNKWARMATLVSIYGFNFTLFLL
>Manes.02G133900
MSKMTNGGEDNKRSKNRRDSPSVEEASNGPLKKGPWTAAEDAILVEYVTKHGEGNWNAV
QKHSGLSRCGKSCRLRWANHLRPDLKKGAFTPEEERRIIELHAKMGNKWARMAAELPGRT
DNEIKNYWNTRIKRLQRAGLPVYPPEVCQQVLNGSQESQNMGTLQNTDACGPDLIQSDHF
EIPEVEFKNLELNRGLIYSPTVLDIPTSSMLKSGVCSSHGSSFMLPTMHPHKRLRESQTIFPSL
DGGVGSGLSAFNQSTDYFSEKITESFDISSQYDSHTNTFGQPPLCVLPGSHALLNDNSSSSEP
LCGAMKLELPSLQYPETQQDSWGTPNSPLPSLESVDTLILSPPAEQTQSDCLSPRSSGLLEAV
LYESQTLKNSKKCSGHQTSDTSVATGDVEDCPFNTYNTEWEVHHDPNSPLGHSAASVFSAC
TPISGSSSDEPGFELKPETTNQVSTPYIEGKEAPKQIDFTRPDVLLGSGWFGLGSGFVNNQSV
RTDEVGACLGDDIDSEC
>Manes.01G163300
MERGGGGGGGGGGFGLGNLPNNPSIYQPTPPLTALDRFLWGRSHFSPQQSQSNVKTNETPV
STNGLYDFTPSTGAIAGVPLPRFQEINFVDALFFDGDNLNCIYERNLNAGLDEEVKVSARIS
KGQAKKSKTGSCVTLIKGQWTEEEDRKLIKLVKQFGVRKWAQIAEKLAGRAGKQCRERW
HNHLRPDIKKESWSEEEEKILVEAHSKIGNRWAEIAKLIPGRTENSIKNHWNATKRRQNSRR
KNKQTENHIEKPQSSILQDYIRSKDLKNTSVTNGTTNVTTPSHSSITTTNTPSSSTTSDDPSSQ
FNYFLSELSEPTLDDSPPLITETYNDELLFLQDFFTNSSMKPLLENVSTNTSMETETSHNVDL
HPKNASVLDSFGLYQDNGDQQLIETSDQCSFFTSTLTSPKTCSESLQAEERPSGHLYSDLYLS

YLLDGGSSCSSSIDYGYDNIMNMELVMDQNFTNGKKDMDLIEMVSSSQFFQGK
>Manes.02G120800
MEGGGGGGAGGFGLRNLPNNPSIYLPFPPLTAVDRFLWGRGQFSQQQNQSNIKNNETLVST
NGLYDLSASNGAIAGFPWPCFQERDFVEGDSLINWTCEINPRACLEGEVNVSGRICKGQAK
KAKKIPCPSLIKGQWTEEEDRKLIKLVKQFGVRKWAQIAEKLAGRAGKQCRERWHNHLRP
DIKKESWSEEEERILVEAHSKVGNRWAEIAKLIPGRTENAIKNHWNATKRRQNSRRKNKHT
ENKIAKPQSSILQDYIKSKNLKNPCPTSIGTPSHSTNTNINTPSSSSTSDDPSSQFNYFLPELSE
PNVDDSPPLITQTYDDELLLQNFFNDNFMESSPEKPATKNPMEMETSSNVDNQLKNPSLVL
DSLGLYQNNGDQQLADTSDQYDLFSSTLMSPIMCPSGLQAEERPTSYLYSDLYLSYLLNGA
TAISPSIDYGDNNSMNMELGIDQTRSNGKKEMDLIEMISSSHFFQGSNST
>Manes.03G156900
MSLGVMAGQLTWGGFIEEGWRKGPWTAEEDRLLIEHVRLHGEGRWNSVARLAGLKRNG
KSCRLRWVNYLSPDLKRGQITPHEESIILELHARWGNRWSTIARSLPGRTDNEIKNYWRTHF
KKKAKLSPENSEKAKTRLLKRQLFQQQQQQQLQQQQQQQMQLNQLDMKKIMSLLDE
NETKVPYMNQTRQDMTTTYPNTTEEHGLLYNLLNANASVPEASNEEFLWDGLWNLDDFH
GNFGVACASTKLAFTI
>Manes.15G047000
MSWGVMAGHLGWGGLIEEGWRKGPWTAEEDRLLIEYVRMHGEGRWNSVARLAGLKRN
GKSCRLRWVNYLRPDLKRGQITPHEESIILELHARWGNRWSTIARSLPGRTDNEIKNYWRT
HFKKKAKVSPENSEKARNRLLKRQQFQQQQQQQQQQQLQQQQQSQQQQLQLLQLNQL
DMKKIMSLLDESENKVPYVPQIRQDMATIYPNPAEEHGLLYNMFNANASVPEASNDEILWD
GLWNLDDVHGNFSAACASGKAGMHNLIAPFC
>Manes.05G170200
MGAMNMCSTSSDSSTSEFSFNGNVTTPRNVNKLERIKGPWSADEDRILTRLVERHGPRNWS
LISRYIKGRSGKSCRLRWCNQLSPNVEHRPFSPAEDETILAAHARYGNRWATIARFLPGRTD
NAVKNHWNSTLKRRAREQQHQMMMEGSFDNSSIGVGVVAAAPASTPRNEEEEVLTALTLA
PPGINGSSSNGRKEAERKAESLPAGFWDVIRNVIAGEVREYMSSTINESSWGFN
>Manes.S048300
MRVVANMPSSSSPPPPSLSKKSSNDDHNDHSDQLRRGPWTLDEDNLLVHCIARHGEGRWN
LLAKRAGLRRTGKSCRLRWLNYLKPDVKRGNLTPQEQLLILDLHSKWGNRWSKIARYLPG
RTDNEIKNYWRTRVQKQAKNLKIDANSSAFQEIIRYVWIPRLLQKIEGSSTSSSPSSSLSTYPT
VSDQPVNCSAPDLPPPPPPQQEVSGHHQGHVDHNSDSEHGSNSCISSTESMNRSQISELSEY
PASPFHSMCTFQKDSFYVDNLDTMTLATLSVTEGGFQNSTCEPHVSESNWVEYDFGDNM
WNMDEFMAI
>Manes.07G099800
MESDKSISAPSDGHNERVQRIQPLHGRTSGPARRSTKGQWTAEEDEILRKAVQRFKGKNWK
KIAECFKDRTDVQCLHRWQKVLNPELVKGPWSKEEDEIIIELVNKYGPKKWSTIAQHLPGRI
GKQCRERWHNHLNPSINKEAWTQQEELALIRAHQIYGNRWAELTKFLPGRTDNAIKNHWN
SSVKKKLDSYLASGLLEQFQGVPLVVHQNQPMQSSSSRVQSCGDDTGPKCGTETEEVSECS
QESVVAGCSQTASGLGNAVLHTREEFQLTEEACLGKEGSSSPASCSEQYFTSVGDVTFSIPEI
PCEVGCPSSFLQQNFSQNPLTCASSDYQFNLQELPNMSSLELGQDSSGLSTHCIAANESHEL
VNVSFQTSMGNITASSAKPDHILISDDECCRFLFSDAMNDGIFSSGNFTKGPNSVACIDSISG
QSSNYQISETDRTTQSFSPSKSGVLTTSCSQPFPSGPSLLSSDDSNPVCGKESNQLTNHSFAAP
EQELIRCEHDDDFIYTNGIDSSPCGDRTDSTCLQEQHYLKEPSKLVPVNTFASGYVTMQSCP

VDEMPNVQTEQQDAGALCYEPPRFPSLDVPFLSCDLIQSGSDMQQEYSPLGIRQLMMSSM
NCITPFRLWDSPSRDDSPDAVLKSAARTFTGTPSILKKRNRDLLSPLSDRRVDKKLEIDMTSS
LTKEFSRLDVMFDESETHRASLLSPSDQKRNSGSTYEDKENLDPALEGRQENGRDCCAFVD
KKVSEKDCDKSDSQDNKKHGTSDADAKNKVHADVEQPSGMLVEDSMNDLLLFSPDQVG
FKGDKAFAPSSRTPKNLYRKILGTLSEKCIALESSPGNSCIVVRSPTFCKKNHDLLPASTSAPL
ENTIDNTENDAGTENLSIFGGTPFKRSIESPSAWKSPWFINSFLPGPRVDTDISIEDIGYFMSP
GDRSYDAIALMKQLSEHTASAYADALEVLGNETPEALLEKRCSNENQENNDVLNNQLENH
SRLASNISTECRTLDFSECEAPGKGTEKGRASTAMTFSSPSSYLLKGCR
>Manes.06G146600
MGCKSSDKPKQKPKHRKGLWSPDEDQRLRNYVLKHGHGCWSSVPINAGLQRNGKSCRLR
WINYLRPGLKRGLFSSQEEETILTLHRLLGNKWSQIAQHLPGRTDNEIKNYWHSHLKKKVL
KGDQGTKEANVYNTTSDNLDNNSPKKITMMQTPDFESLLMNMENSSTDADQSVPRMSES
PKQPNRSSLPKLMFAEWLSLDSFSSLSCEGIMSSKDQNVSSSFQDNFMQGCFLNEGTFGNG
EYRNLLSYASPDDMFSSQLKFECQSSGNEQFVDFSCGEDICSEFNMRSEL
>Manes.01G118700
MGRQPCCDKVGLKKGPWTAEEDNKLINFILTNGQCCWRALPKLAGLLRCGKSCRLRWTN
YLRPDLKRGLLSEYEEKMVVDLHAQLGNRWSRIASHLPGRTDNEIKNHWNTHIKKKLRKL
GIDPLTHKPLPATETPPQEQQQAMAELQQNKETKTSAESRITEEETLEEDKRMTSTFETIEPT
NNSFCVDEVPLIEPHEMLVPCTAASSSSTTTTSSSSSSSHGSNNNLFLEGFQFADFEWPDNAI
DDLWGDDLSSSWDLLINDADSDRKQLHSHELHHHHALINQCSKMVFDQDSWTHGIS
>Manes.02G173200
MDEPVASNSCDDTKTGTTCPRGHWRPAEDDKLRQLVEQYGAQNWNSIAEKLQGRSGKSC
RLRWFNQLDPRINRKPFTEEEEERLLAAHRIHGNKWALIARLFPGRTDNAVKNHWHVIMA
RKQRERSKLSGKRSYQEGLSDSSSTPDDLHPRKSRSQDLFSSRIGFENGTRVLNFRNLSADR
IFSVVSSSPSWTFAPAAITVSNTSPSAVEHLSSRKEGRDCFNNSSFYTTESFNISDHTSIYRCYP
KSSMHRSSTVFGLPNYRRVVPSPFGYLKLEDEHENNIGMIKNEQVNFWDNSSMFKNNMKV
SKQAEQGDQESIRHEEVQFIDFLGVGISS
>Manes.01G092500
METLTDMGSQDICRSEPSDNKEAHHHLMSSSLLVPKCCDGSLAANQNSSFDDKDKEDKND
ECSRISGQSSKLWARGHWKVAEDAKLKELVALHGPQNWNRIAEKLQGRSGKSCRLRWFNQ
LDPRINKSAFSKEEEERLMAAHRICGNKWSLIARLIPGRTDNAVKNHWHVIMARKYREQSL
AYRRRKQTNDAKRRVQDGSGSASRDTPMNEVVAKSLNLCSGRIIEPSHHFPLAGFKSEGFY
DFTSSEGAGRSGHKSSDIVSQMEHCSFAVLQNKQQSSNHHFSAGFSYSMASPRSQVSEPSSS
SSLSFAENNATSDSVTTISPPFIDFLGVGAS
>Manes.02G047300
METFTHMAPYNICSCHTLNVHKGDHYMAFLSSPMPKCCTFSFGSNQNNDVEDKCNAGISL
ISDQESLMHHQQSSKLCARGHWKPAEDAKLKELVALYGPQNWNLIAEKLQGRSGKSCRLR
WFNQLDPRINKSAFSEEEEEKLMTAHRVYGNKWALIARLFPGRTDNAVKNHWHVIMARK
YREQSFAYRRRKQTEAAKRRVEDGSSSATRETAINVTETKSPYLCNAIITKPSLHFPLEGCKG
FCDVTSSEEAATSGTVLFVGSSNLLSLPHGSCPEQTPFDFFSGHKSYEISSQNGTTFTVSHNM
QQSSNHHLSAGFSDSMASPTSRVSNCEPSSSSSISSSDNNATCHFQTTISPPFIDFLGVGAS
>Manes.09G092900
MKERQRWRAEEDALLRAYVKQYGPREWNLVSQRMNTPLNRDAKSCLERWKNYLKPGIK
KGSLTEEEQRLVIRLQAKHGNKWKKIAAEVPGRTAKRLGKWWEVFKEKQQREQKENNKT

VEPIDEGKYDRILETFAEKLVKERSTPAFVMATSNGGFLHTDPPAPAPTLLPPWLSASNSSSA
VRPPSPSVTLSLSPSTVAAPPPIPWLQPERGQDNAPFVLGSLPPHGTVAACENLMISELMECC
KELEEGHRALAGHRKEAAWRLRRVELQLESEKTCRKREKMEEIESKINALREEQKASLDRI
EAEYREQLAELRRDAETKEQKLAEQWAAKHLCLSKFLEQMACRPRLAESNGR
>Manes.11G127600
MCTRGHWRPAEDEKLRELVERYGPHNWNAIAEKLQGRSGKSCRLRWFNQLDPRINRSPFT
EEEEERLLASHRIHGNRWAVIARLFPGRTDNAVKNHWHVIMARKCRERSRLQAKRAAQNL
VNDHKLSSSSSSSKQDRVLFNSETGNNLASFIDKYYCDRYYNQYPFPHNYLLRPFCQASEN
NPSLCEGKKQAIEFYDFLQVNTDSSRSEVIDNAKRDDEEVDQQAMEQQRKAGLQFIDFLSV
GKYSS
>Manes.17G033000
MALRKGKWRPEEDHKLICYIRRYGIWNWNEMPRAAGLSRSGKSCRLRWMNYLRPFIRHG
NFSKEEEDTIFKLHEKLGNRWSAIAAKLPGRTDNDIKNYWNSNLRKRLRNTASATKLDGLQ
TSGGQLKKHLSEGTVPKEIPKSSSSNMANKTDINQDVAHQNITAGLSKVSLDFKSLQGQSFS
MEGLHIREDNGKILHPFASDDDLLGFFPPMQNIDFSGELQYLTWHEEPVYSYNYQDNLFEY
PTLWYDEYRVIEENNNNELSELSGEVFQSLWEQQSCPIMEDLYTAKDESEREGAGHPFEDFS
ACYDSNQLSSGEIQFLWDYPFF
>Manes.17G033100
MALKKGKWSPEEDHKLISYIRRHGIWNWNEMPRAAGLSRSGKSCRLRWMNYLRPFIKHG
NFSKEEEETIFKLHETLGNRWSAIAAKLPGRTDNDIKNYWNCKLRKRLRNAASATKLDRLQ
TSGGQLKKKKHHLSESTVPKETPNSSSSDIFNKTDINQNVAPQNITAGLSKVSLGFNGLQAQ
SFSREGFHIMEDNGKTLYHPFASDDELLGFFPLMENIDFSGEAQYVTWHEEPVYSYDYQDD
LFEYPTLWHDEYQAQQPCPVIEDLYTATAKDEREHEGPGHPFDDFPACYDRNQISGEIQFLW
DYPVLL
>Manes.18G085700
MLLYIFFSTSLMVYFDDQEFKDKGCQHRMYCWHFSIMDEPGASSSYDDAKNSLTCPRGHW
RPAEDEKLRQLVEQYGAQNWNSIAEKLEGRSGKSCRLRWFNQLDPRINRRPFTEEEEERLL
AAHRFHGNKWALIARLFPGRTDNAVKNHWHVIMARKQREQSKLSGKRSYHEGLRESNSST
PDDFHPSKSRSEDPFSSRIGFENSTRVLEFRNPTADSWTFAPSNCSYSVADHLSPRKEGIDFFN
NSSFYTTQNSKISDHQSIYRCYPNSSMCRSSTVVGLPNYKRVVLRPFGCLNFEEDRENHGMI
KKDLASFCYSSSAFKKNNQAEQGDQGSVRHKEVQFIDFLGVGISS
>Manes.01G117100
MEGLFGVRKGAWTPEEDNLLRKCVDKYGEGKWHQVPLKAGLNRCRKSCRLRWLNYLKP
NIKKGEFEADEVDLIIRLHKLLGNRWALIAGRIPGRTANDVKNYWNTHLRKKEASARINAK
KHSTPTMTKASIIKPRPFNLPKKVFWSSNGKTPVTLTTHVPVIDNNNTLCKPCMPSPPLDDH
LNGWLESLFNDNEVKQKDTSCASGSGLRRDSQQWAFRRRNECSSCACGRHRGRRRGFQ
>Manes.15G167600
MESENQDKKRLWTEEEDKILVDYVKIHGKGRWNHISKKTGLRRCGKSCRLRWLNYLSPNV
KRGNFTEEEEDVIIRLHNLLGNRWSLIAKRVPGRTDNQVKNYWNTHLTKKLAAKDQNGRL
SIKQQSSKVAVTSSTISKVATNSGFSEGTATDQITVDIEIQRDTQVSDLHGLTFMDSYANPFW
HYYDPLELTTLGNEFLDGYSFDHA
>Manes.08G083200
MEIEMKEKTVVEVDGTNSGGGDVEDGAGSGDDLLHAVGEGGGSSRARRDRVKVPWSPEE
DAILSRLVSRTGARNWTLIARGIAGRSGKSCRLRWCNQLDPAVKHKPFTDEEEHIIATAHAV

HGNKWAVIARLLPGRTDNAIKNHWNSSLRRRSTERGRTRLASGNVDQDANLDKPKASSEE
TLSCGDANSFKSIEGKNVSSLDNLDDQCEEKAPLEIPSNNEAKESSTLFRPMARVSAFSVHN
PEIASPCPRPVPMQGPSIEAPIPDNGICKLLDRIYNERLVPHQCGHGCCKAQNGNNLVNSLL
GPEFVDFLDPPSFPNFELAAIATDISNFAWLKSGLENKTVPNDKAARITPCGSQLQMGHL
>Manes.08G151800
MGEIVDLNFLPPPAASQLSLVNTFGVIGKPIETETINTHVGFQILRHSMDLNGNPWSIHQVEN
RGTKRPNDGSDGLFGVQKKALSLNLDEEEEPNGSTPGKNGYTKLCARGHWRPAEDAKLK
ELVAQYGPQNWNLIAENLQGRSGKSCRLRWFNQLDPRINKRAFTEEEEDRLLAAHRLYGN
KWAIIARLFPGRTDNAVKNHWHVIMARKHREQSSIYRRRKPSSSSKVVPPIKELDVNLQTN
ACSESTTISSTIDESASTCTDLSLSPSSTKPHPKLFTRFSLQGAPMDSSAEKEVMMRNIAFDK
FYSCSGGKELYQTGSMGVVRVMDQCGQSDSNSEVSGTESVGTNRISPSLFGDNEDGNQKI
NSAFIDFLGVGAS
>Manes.09G063000
MGWAKIARRLPGRTDNEIKNYWRTHLKKKLQAQEGKQDFLYQKTDPSSWNYDMEEYKS
VGTTESSLDQNYELSSFTYLNSPYETRLHDWMSSELSGDQTEVKIHGDSGGFDSSFCYLKL
NSEDGDTSVSDSLGSLWDMN
>Manes.14G064900
MFISDNQSIKPSIKNGFPFELPSLPSKGFLEDFHHFDQFYANGLSLNPSFGVQSGCNFNSFDA
FPYGSSTNMDFYDYECKPFADTNGGHGQVMDNFQSGRYLNLPESNPTDMMVSNQNNNM
SLNFQEVKPINFLVPDEVSCVSANHEYHKKVGLNKNRASPSARRTWKGCKKNNLVKGQW
TIEEDRLLAQLVEQYGLRKWSHIARMLPGRIGKQCRERWHNHLRPDIKKDIWSEDEDKVLI
QAHAEIGNKWAEIAKRLPGRTENSIKNHWNATKRRQYSKRKCRSKYPRGSLLQEYIKSLNL
DSVPGRYQGTIKLKAKSSAAIDTTAVNMKARNQLQPQAASNVFPNDNRSVPNCYDFNEVP
DFDFDEKLFQEGCSFDSLLDEIANCDHVVDEKSFEQMDAAVEDVNPLFMDFEVKKELDLV
EMISQSKM
>Manes.05G038200
MVEEAALSDSEKGGLVSARPSVHDTSANNAVTDPISVKGRTTGPTRRSTKGGWTEEEDKIL
VAAVEKFNCRNWKKIAECVPDRTDVQCLHRWQKVLNPDLVKGPWKREEDDLIRDLVGKQ
GIKKWSEIAKHLPGRIGKQCRERWHNHLNPEIKRTAWTKEEELTLIDAHKIYGNKWAEIAKF
LNGRTENAIKNHWNCSVKKKIESCSARKFDIHSYNKGAEIRKSEMDNQSFDERMNPERSM
HACPLDLALGNSKTREFQLSASDKGNCKYAVKELYFGTLDAKPSPAFTLTSVEWKGSDNN
ANKIEHTNHLSNWSSKLCNTSSDDVARLQLPCERTLELSKTVNSLHSGALSAPLTIASSTVP
GYDIKTAELDDKKKGAGDPESVELNCGLLSSESLQLDKVLLQTGSAPSTGSYPRTTTLSPVS
SCAPLSHNGRISHDRSSPESILSILRSAARSFKNTPSIIRKRSSTSRAETFGDTKSVEKRLEYAF
NIGCDSDGKRFGDS
>Manes.15G108200
MRNPSPSASAGEGPQPAKTPCCSKVGLKRGPWTPEEDELLVNYIKKEGEGRWRTLPKRAG
LLRCGKSCRLRWMNYLRPCVKRGQIAPDEEDLILRLHRLLGNRWSLIAGRIPGRTDNEIKN
YWNTHLSKKLISQGIDPRTHKPLNLESFDHQKASSSKANRKTSGLMSNNNNSTVAAPSSGV
EETNSGGSTQIISNKENVCIENTSLDHHYQLTGNNNADPSHGYSSLLNCGNGTCGMHLISN
AQGLSNEEDEDINYGTDDVFSSFLNSLINEEAFTSQQMQQEGNLIVASSDPLLSTSTSTFGYG
PSWESVLMSPTNQNDPSKRVNDHLH
>Manes.03G098700
MRNPSMSASSGGAQAVTMAKTPCCSKVGLKRGPWTPQEDELLANYIKKKGEGRWRTLPK

RAGLLRCGKSCRLRWMNYLRPSVKRGQIAPDEEDLILRLHRLLGNRWSLIAGRIPGRTDNEI
KNYWNTHLSKKLISQGMDPRTHKPLKPECLDQNKASSSKATKSKTSSPKSNNKNPAAGAG
AGVEETPSGDKENVYIESTYIDYQYQITGNSNPNHGYSSLLNSGYGNCGMDFRSIIHGPINE
HGDINYCTDDVFSSFLNSLINEEAFTSQQHQQMQQQDNLIVPTSDPLVSLGASTFGYGPSCE
SELMSSTFNQNDPQQEG
>Manes.11G020400
MDPSTHDESVKREDKPVETMTNIPTKKEVNKGAWTPEEDRKLAQVIAIHGAKRWKIIAATA
GLNRCGKSCRLRWLNYLRPNIKRGNISDQEEDLILRLHKLLGNRWSLIAGRLPGRTDNEIKN
YWNSHLSKKINQKEKQSGASTREESMDGKMTAGKVDIVEVREENMSCNNTREEDSNATF
NVDDFFDFSNEDPLDLEWMNNFLEMDEGFTSM
>Manes.01G117200
MERVLESTRWSYELWCLLKLVFWVNRFKSNIVIGSNRSGKSILHRWSLIAGRLPGRTANDV
KNYWNTHLKKDASARINAKEDKRIFGPVVEKPLLLLTTHVPVNDNNNTLCKPCMPLPPSN
DHVKWWESLLKENEVNQKDTSCDSGSGETHISRLSGEEMNAAAVQVGVTKVGDVGFNKE
GHIWGSNFYFDVGLWDLFDTHVNIP
>Manes.16G011800
MAESERSSSDETFVNSQAESNEEKTLEFSEDEEALVIRMYNLVGKRWSLIAGRIPGRTAEEIE
KYWNSRYPTTE
>Manes.09G136100
MSVQHLRSESNSFSRYQNVNFLPPPAASHLSLATFGVMSKRRDTETVPPQMGFHISSHSMD
QKGRQWSIQQVENRGTKRPHDGSDGIFGVQKKDLSLDIGEEEEPKSCATGKNGHTKLCAR
GHWRPAEDAKLKDLVAQYGPQNWNLIAENLEGRSGKSCRLRWFNQLDPRINRRAFTEEEE
ERLLSAHRLYGNKWAMIARLFPGRTDNAVKNHWHVIMARKHREQSSIYRRRKPSSSSQIAP
PIKGLDVNSQKNACSESTTISSTIDESASTCTDLYLSPSSTKAPPMLFTRFSPQGAPMGPFAE
KEVAMGNVELDKLYLSRGKGFYQAGSIGLVTGLDQSGQSDSNSEVSASESVGTNGIGDNE
NRSQKINVTFIDFLGLGAT
>Manes.06G146700
MTSLEKKTRAPREEEVKKKKMGRTPCCDKDGVKKGAWSPEEDRILVQYIQKHGHGSWRS
LPKNAGLLRCGKSCRLRWTNYLRPDIKRGPFTPEEEATIIQLHGMLGNKWASIASQLPGRTD
NEIKNFWNTHLKKRLSSLDQKLQISCSSSEPNAKCDSPSTRHMVQWESARVEAEARLSMES
LLLNPSSSVKMEYDYFLQLWNCEVGESFRNINGKVGEACESPISQASSSTKFGSGDNENAA
QMMSIKQETVHEQEDNCKPNADVITGSDSISSNEFIDYSDTVLKMLLDVPVGNGMEFLE
>Manes.05G114400
MRAVTMSSLSSSSLCKKKSLSSSSDCDNSDQLRRGPWTLEEDNLLIHYIACHGEGRWNLLA
KRAGLRRTGKSCRLRWLNYLKPDVKRGNLSPQEQLLILDLHSKWGNRWSKIAQHLPGRTD
NEIKNYWRTRVQKQARHLKVDANSTAFQDIIKRFWIPRLLQKIEGSTTSSSSSSSSTILSENPT
VVAHQPVNYAAQNFQYPIPPPPPQEVPGNHQGRHDHNSDSENGSNPCISSTESINISQISQVS
EYPASLFHGMGSFPKDSYYVDNMEAMSLASLSVPAGVVQNLGESNWGGYDFGDMWSMD
ELMAI
>Manes.15G003000
MMDFKARYSTHKITGSSSSTTTTQSEEEMGGLRRGPWTAEEDFNLINYIATHGEGRWNSLA
RCAGLKRTGKSCRLRWLNYLRPDVRRGNITLEEQLMILELHSRWGNRWSKIAQHLPGRTD
NEIKNYWRTRVQKHAKQLKCDVNSKQFKDTMRYLWMPRLIERIQAANTTAASSTTAACTI
GSGTTTEATHHHHLNDNTNTDVGSGQWVVAHVGVFGGDFGVAQVIPTTYNTPETWNTAA

SSESFGTHIWPNDHFSLSVNPDNLQPEQVGYSESMISPSGYFNQVLDFQAMEHDNNLWVE
GGYTSDNLWKVEDMWFNM
>Manes.05G177900
MASTDREEIGRIKGPWSPEEDQALKRLVQNHGARNWSLISKSIPGRSGKSCRLRWCNQLSP
EVEHRPFSPEEDEAIIQAHARFGNKWATIARLLNGRTDNAIKNHWNSTLKRKCSSLFEDLSD
DAHAQVQQPLKRSASVGAATVSGLQLNPSSPSGSDVSDSSLPGMASSPVYRPLARTGSLQA
GLSIDAASSTTDPPTSLSLSLPGSDSFEASNQVSIIGSGSGFNHGVTPIHVVQTQLVLPTAVPV
EQAATTQQNGVGFEKQFFRPEFLAMVQEMIRKEVGRYMSGI
>Manes.18G042000
MASTARKDVDRIKGPWSPEEDEALQRLVQNYGPRNWSLISKSIPGRSGKSCRLRWCNQLSP
EVEHRPFSPEEDETIVKAHARFGNKWATIARLLNGRTDNAIKNHWNSTLKRKCSSLSDDLS
DDGNAQQPLKRSASVGAGTNISGLQLNPNSPSGSDVSDSSLPGMPSSPVYRPIAKAGSLVSP
GLLIDAVSSTTDPPTSLSLSLPGSDSCEASNPISISGSGSGFNQAVNPIQDVHTPVAQPAAGAV
QQAPAGQYKGIGYEKQFFSPEFLAVMQEMIRKEVRNYMSGIEQNGLCLQTEAIRNAVVKRI
GISRIE
>Manes.14G115200
MENKQVKPPIKKGLWKPEEDLILKTYVETHGEGNWATVSEKSGLMRGGKSCRLRWKNYL
RPNIKRGEMSQEEEDLIIRMHKLLGNRWSLIAGRLPGRTDNEVKNYWNTHLNKRCRTGKR
KPTDPSNHQNGNDKYKSKKQCNSQTTSSTSPKSNPEESDGKKKEKEESTVTNTWIQDAQS
MNYYIESPVMPVCNDAFVLNDEPFIAYWDSFVLFESFGL
>Manes.12G108500
MAVTGKDVDRIKGPWSPEEDEALRKLVQKHGPRNWSLISKSIPGRSGKSCRLRWCNQLSP
QVEHRAFTPEEDDTIIRAHARFGNKWATIARLLNGRTDNAIKNHWNSTLKRKCSMSAVDD
ACFGGRDVYDGNLDGNSQPLKRSVSAGSGMAVSTGLYMNPGSPSGSDVSDSGVPVLSSSH
VYRPVPRTGPVIHPVETTSSSNNNDPPTLLSLSLPGADSSEVSNRPAESTAVRVADSTPATNIIS
LMPAVNQVPSPAPASEVAVVGMQQRAVNGGLESGFVGFTADFMAVMQEMIRREVRNYMM
EQSGGGGGSGGGGGMCFQAIGGEGFRNVVMNRVGMSKIE
>Manes.13G118400
MAVTGKDVDRIKGPWSPEEDEALQKLVQKHGPRNWSLISKSIPGRSGKSCRLRWCNQLSP
QVEHRAFSPEEDETIIRAHTRFGNKWATIARLLNGRTDNAIKNHWNSTLKRKCLSSAVDDG
SFGSRDGYDGNLGGNCQPLKRSVSAGSGMPVSTGLYMNPGSPSQSDVSDSSVPVLSSSHV
YRPVARAGPVFPPTETTSSCNTNDPPTSLSLSLPGADSSEVSNRVAESTPPTNTISLMPATTQV
PPPATATATAQAQVAAAAVAAGMQQQAAVNGVGGGFVGFTADFMAVMHEMIRREVRNYM
MEQSSRGGGGGGNGGGVGMCFQAAGGEGFRNVAMNRIGVSKIE
>Manes.02G058900
MMDWGVVQQGWRKGPWTPEEDKLLSEYVKLHGEGRWTSVARGSGLNRSGKSCRLRWV
NYLRPGLKRGQITPQEEGIIIELHALWGNKWSTIARYLPGRTDNEIKNYWRTHFKKREKSSS
HKQEKRKAQVLNKKLQLQQQEQQQQQLGDDKMKAINFTSEGKIHEAQEKQEMAFMG
PDLESQCLPVMYQDIPSWADFMVEDGVLWGGLWNLDDQVDHASNCSKIAKQNQATAFSF
AGGSDNTYSSAGGYIF
>Manes.02G074300
MRKPCCDKEGNNKGAWSKQEDQKLIDYITTHGEGCWRSLPKAAGLHRCGKSCRLRWINY
LRPDIKRGNFAQDEEDLIIRLHALLGNRWSLIAGRLPGRTDNEVKNYWNSHLRKKLINMG
MDPNNHRLNQILPRPQPERVSSPVDENICKTKKSKGDYNDRTSDAPSSLEEDETGAGSSDIN

LDLNIAVPSPAVNTATLETRPENCDASATGEVRSETLPALLLFL
>Manes.17G075600
MRKPCCDKQDTNKGAWSKQEDQKLIDYIRKHGEGSWRTLPQSAGLLRCGKSCRLRWINY
LRPDLKRGNFAEDEEDLIIKLHALLGNRWSLIAGRLPGRTDNEVKNYWNSHLRRKLVNMGI
DPNNHRLNRNFPRLQNPQGSFSATSSELKSDTTKTRHDNNNNNNEQASDAASCLEDSPIAL
PDLNLDLTMSIPSSRSFNISPEAKQKITESNLSKEPEFAASSPTLLLFQ
>Manes.17G034300
MRAPNSDQIPLKKGTWSPEEDHKLIAYINRYGIWNWTQMPKAAGLSRSGKSCRLRWINYL
RSNIRHGNFTKQEEETIINLHEMLGNGYSN
>Manes.03G072300
MGHHCCSKQKVKKGLWSPEEDEKLIKFITTRAHVSWSSVPKLAGLQRCGKSCRLRWINYL
RPDLKRGSFTAQEEKTIIDVHRILGNKWAQIAKHLPGRTDNEIKNFWNSCIKKKLIAQGLDP
NTHKLLSPNYAQSYNNNTACTPSADSHYQPTSSSSVFHATSQMKDLSMDVKETPFTPSLTSI
ASHDANYFCLHPLHISSTLTTFCEHQNSNIQALLDHASQSSPMGSVPISCSATNPSGFGIIDEK
NLWGGYTEPIQPLRNEQMKVEQAVQFERTSEVYAGQNMDSLFQSSNFNFHFMECTQMPE
MYYSVNPIDQLTWDSQILQ
>Manes.16G058600
MEHQCCSKQKVKRGLWSPEEDEKLIKFITNHGHGSWSSVPKLAGLQRCGKSCRLRWINYL
RPDLKRGSFTAQEERTIIDVHRILGNRWSQIAKHLPGRTDNEVKNFWNSCIKKKLSAQGLDP
NTHKLLSPSYRKNCYNNTPCGLSADSIYNPISSPSAFSIVSSQMKDFSMDEKQTPFIPSFLSIP
PPDSSTSSSLHPLHASTTCERQNSDIQGSHDHASESISMASVNTSCFDSNPSGFEIIYDSRLWN
DAIKPIQSSRHEEMLVEQVVEIGKTNEYLSAGQNMDASFESSNFYLDLDFAECTLLPEMYY
SASSIDQLTWDLQAL
>Manes.03G077500
MGHRCCTKQKVKKGLWSPEEDEKLINHITTYGHGSWSSVPKLAGLQRCGKSCRLRWINYL
RPDLKRGSFSAQEEQIIIDVHRILGNRWAQIAKHLPGRTDNEVKNFWNSCIKKKLISQGLDP
KTHNLIPSRQRSNNKFAQAQAIILQSHQQPFSIITVNSQMRDVSMEMNSPILTPPAAPPDITQ
QPSSLQTSSGPSIFTSGDHQNPNILWTANGRQNSLDSAVFPSIQSTLISRASSPVNGLLDENFS
WGSNPIGENFEAPRMEVVKAQEQENNQAKENVDAANGVQDMDASFDSSCFGLDFVESTL
FSSSMCRELSSMDDLAWNF
>Manes.16G046100
MGHSCCRKQKVRRGLWSPEEDEKLINYITTYGHGSWSSVPKLAGLQRCGKSCRLRWINYL
RPDLKKGSFSAQEEQIIIDVHRILGNRWAQIAKHLPGRTDNEVKNFWNSCIKKKLISQGLDP
KTHNLIPSHQRASNKVAAGNMLQSQQQPFSIITVNLQMTDPSMEMNPPIITLPAAFSPNAIIQ
RPSSIQTSSVPILTSVDNQNPNILWTVNGRENSLDSSIFPCVSSIQNTPISPSVTPSWFGILDEN
CFWGHNTIAENFRAPRMDVLQAQGEENNQANEKVDVAEGVQDMDASFDSSSFGLEFVES
TFLSSSTCGDLGSMDDLAWNF
>Manes.01G188000
MQETKKKNNGGNEDSKKKERHIVTWTQQEDDILRQQISLHGTENWAIIASKFKDKTTRQC
RRRWYTYLNSDFKKGGWSPEEDMLLCEAQKIFGNRWTEIAKVVSGRTDNAVKNRFSTLCK
KRAKYEALAKENRKTYINSNNKRILFHNGFNADGILENAAPDKRTRRSHIPDHSEHCKLAD
RSHPKQQSRPPLADTGSTLTPYTTAGKTEQNQVEVGELHEDVPNELQSVLTKEQSNTDECE
KGIISLPNVTQGGTFPSFDEDTNADVVVSVSSSTEFSSPFQVTPVFRSLAAGIPSPKFSESERN
FLLKTLGVESPCLNPSTNPSQPPPCRRALLQSL

>Manes.05G098700
MQETKKKNNGGNEDSKKKERHIVTWTQQEDDILRQQISLHGTENWAIIASKFKDKTTRQC
RRRWYTYLNSDFKKGGWSPEEDMLLCEAQKIFGNRWTEIAKVVSGRTDNAVKNRFSTLCK
KRAKYEALAKENKNTFINSNNKRILFHNGFNADGTTENATPAKKTRRSHISAHSEYCKLAD
RSHPQCGNQQSRPPFAVLAQNLHNVNVAGQHQAKEVSGDASQDSKTQGTFLRKDDPKIAV
LMQQAELLSSLALKVNAENTEQSLENAWKVLQDFLNQKENDILRYRFTDMDFRLEDFKDL
IEDLRSSNDGSRPSWRQPDLYEESPASSEYSTGSTLMPYTATDKTEKTPVEIGALHEDIPNEL
QSVHVKEQSDIDECEKESISCANMSQGEIFSSFDEQANNDIVVSASSSTEFSSPLQVTPLFRS
LAAGIPSPKFSESERNFLLKTLGVESPCPNPSINPSQPPLCRRALLQSL
>Manes.07G005800
MRKPDLMGKERVNNNKAKLRKGLWSPEEDEKLIKYMLTNGQGCWSDIARNAGLQRCGK
SCRLRWINYLRPDLKRGAFSPQEEELIIHLHSILGNRWSQIAARLPGRTDNEIKNFWNSALK
KRLKIIGNNNNNNPSTTSPNESDSSEPRDHVVGNGMSMHHDHDLMTMCIDSSSSSSASIQA
MVAGNANGNQFDPFSILNNNRFEGTAAAGLFDMPTCLTQVGMGGDGFYGDYGILESHHN
KVGLERDLCLPPLESSRSLEEENNNNNNTNTNNVVTNHSIISMKSNINHNNNINNHLINNNS
CFNNTDHHQLQSLKVEDMFGFENHWQGENLRMGEWDLEGLMENISSFPFLDFQVE
>Manes.10G143200
MRKPDLMGKDKGVMNNNKAKLRKGLWSPEEDEKLIKYMLTNGQGCWSDIARNAGLQRC
GKSCRLRWINYLRPDLKRGAFSSQEEELIISLHSILGNRWSQIAARLPGRTDNEIKNFWNSTL
KKRLKINNNNPSTSSPNNDSDSSEPRDHVIGNIMPMHKDDLDLITMCMDSSSSSSASIQPMV
GAGGGNQFDPFFILNNNQLDFTGAAALFDMSTCLNQVGMGDGFYGDCGILESHHNEIGIE
RDLCVPPLEICSRSIDEEEEKKTNNNAVTNHSIINNNNIINNHLNNNNSCFNNTDHHLHHQN
FKVEDMFGFENHWQGDNLRMGEWDLEGLMDNISSFPFLDFQVQ
>Manes.01G231300
MDTQVRNHDCATYQNEENIDIKKGPWTAEEDVILAEYVAIHGEGRWNTAARCAGLKRTGK
SCRLRWLNYLRPDIRRGNITLQEQLLILELHSRWGNRWSKIAQQLPGRTDNEIKNYWRTRV
QKQAKQLKCDVNSKQFRDSMRHIWIPRLIERIQAASRSPTDQSTTYSSYKDNHNDIPPSSEA
VQMNDPMIEWMMPEPSRTSSESLETQVSLVSSVTKYQNQPNKQNLYGLYSEEESNRWVEM
ETLAEESLDSLWNQENIWFLQQQLI
>Manes.05G012100
MGAQVRNYGCATYQNEEDADIRKGPWTVEEDAILAEYVAIHGEGGWNAAARCAGLKRT
GKSCRLRWLNYLRPDVRRGNITLQEQLLILELHSRWGNRWSKIAQYLPGRTDNEIKNYWR
TRVQKQAKQLKCDVNSKQFRDAMRYIWMPRLVERIQAASGSSTGHSNYSSINHNGVPISNE
TGEINNPMIELVMPEPSGSSLESLDTQVSPVSDVTEYQNPTSVQNVSGLYPEGESDRWIEME
MQSNIVNGGESLESLWNEENIWFLQQQLM
>Manes.01G034800
MANNCVIYSTSEKNPNCFSPSSSSSSSGMVLVDIASLSLSPSYGVIPPSSSSSSSSMETGRGSW
VFSLMGKGSDCSDVFLENNDTENHNANSNDENPNNENINSGKETDSGQSKLCARGHWRP
AEDTKLKELVAHYGPQNWNLIAEKLEGRSGKSCRLRWFNQLDPRINRRAFTEEEEERLMQ
AHRLYGNKWAMIARLFPGRTDNAVKNHWHVIMARKYREQSSAHRRRKLTQSVYRRSEET
SSFLCRDPATRSEPPPPPPPTYCLNIPNAVGLTNLSPYPVGPFNGGVDYGLNGSRIQLPLTGFC
AQQTPFDFFPGPKSNDMIGSIFSHRPADEPHNSSFYPQRHHDTMAMQQSNYQTPYYLSAST
PPPPPPPPPPQVSATEPSPSSSSSSVAENTRTV
HFETIPPPPFIDFLGVGAT

>Manes.05G103300
MASKCVIYSPSENNPICYSSSPCSSSSHCSSAGMVLADIGSLSLSPNYGVIPPASSSHELEIERS
SWVFPFTGSHQSGDVVLEGKGSDCSDAFGENNDTANRNANSIDENPNNENMNSGKETDSG
QSKLCARGHWRPAEDTKLKELVALYGPQNWNLIAEKLEGRSGKSCRLRWFNQLDPRINRR
AFTEEEEERLMQAHRLYGNKWAMIARLFPGRTDNAVKNHWHVIMARKYREQSSAYRRRK
LSQSIYRRSEETSSFVCTDPGSKAEPPPYCLNIPNAGGLTSLSPYPIGTFNAGVDYGLNGSPH
MTSGGEAASSIQVQVQVPLTGFCAQQTPFEFFPGSKSNDVMGMFSHSRSWDRPSDEPHISG
FYPQLHDSYIMAMQQSNYQNPYYFSADSKASTPPQVSATEPSPSPSPSPSVAENTRTGHFETI
PPPFIDFLGVGAR
>Manes.07G131800
DATIVQAHALHGNKWATIARLLPGRTDNAIKNHWNSTLRRKRAADLSSASSESNSVMKRP
YVDVSVESGSGSDSGVKNKRQSLGASPEYSSFDGDARIMGPETSLTLSPPGDGFVSVATVG
EKVEEVEVNGGEECGERRRENKCGVQIEETCLLTIMQRMIAAEVRSYIDRLRAEDGLDGPS
TRKDL
>Manes.03G208500
MMTMMDVKGINSNRKSSSSSSSSSTTQSEEEMGAHDLRRGPWTAEEDFNLINYIATHGEG
RWNSLARSAGLKRTGKSCRLRWLNYLRPDVRRGNITLEEQLMILELHSRWGNRWSKIAQH
LPGRTDNEIKNYWRTRVQKHAKQLKCDVNSKQFKDTMRYLWMPRLIERIQAANTNATSTS
TSTATTTAAATTGVANITTDPTYHHHLINNTDMGNRQWAAVVHGGAAGLVGNEFGAAHVT
PTTYTTPETSSTGASSDSFGTRVSPNDYYNMNPDYFQSVQVGYSDSMISPSGYFNQVMDFQ
TMEQNNHQLWVDGGDTSENLWNVDDIWFNM
>Manes.04G153700
MSRTTNESDDGVLSKDQTESPLAEEGSCGGSANGGVVLKKGPWTSAEDAILIEYVKKHGE
GNWNAVQKHSGLSRCGKSCRLRWANHLRPNLKKGAFTQEEEQLIIELHAKMGNKWARMA
AHLPGRTDNEIKNYWNTRIKRRQRAGLPLYPPEVSLQALHESQRGLNISGINSGDKGHQDL
CRTNNYEIPDVIFDSLKANHGISPYVPELPDITASSMLMKGLGSSQYGSFMLPTIHRQKRLRE
STTLIPGYGGSVKTEFPLFDQFQGNPCDKVAQSFGLSFPFDPDPTNKNPQSFGDNQGSHTFA
NGNFSASKPASGLVKMELPSLQYPDIDLGSWGTSPPPLLETVDTFIQSPPMGTVECSPRNSG
LLDALLQEAKTLSSGKHHSSEKSSNSSTVTPGELAESSALNKCKTEWEDYGDPLSPLGHTA
TSLFSECTPMSTSGSSLDETPVTETLTGCNVKSELTTRAWSPEREQETTTRLNITRPDALLAS
DWIEQDSSYVKDQVVMTDNIASLLGDDLSSDYKQMSAEASTSNEGWGLSSCAWNNMPAV
CQMSEFPSENCYLSNL
>Manes.06G066600
MRSMRAPSVANRMCNSREDESELRRGPWTLEEDTLLTHYIARHGEGRWNMLASYAGLKR
TGKSCRLRWLNYLKPDIKRGNLTPQEQLLILELHSKWGNRWSKIAQHLPGRTDNEIKNYW
RTRVQRQARQLNIESNSKSFLDAVRCFWMPRLLQKVEQNCYSSPSSTLDSQTHAIASASSSN
FEQVANSFSSETFPPQPKLTQYSNPASEHSCSVTSSSVLSTDFIPISHQTEILENPASSCPPLLDS
TVYNNLLLSDIYYVENSGYGMDAFNPASMPEFDTFGDSTSECNMADGNWVFDDYMADPL
WNMNDMWQV
>Manes.S051400
MISNGGAAKETGLSGQKATNQSLKKGPWTATEDAVLIDYVKKHGEGNWNSVQKNSGLMR
CGKSCRLRWANHLRPNLKKGSFTPEEERIIIELHAKLGNKWARMASQLPGRTDNEIKNFWN
TRMKRRQRAGLPIYPQEFQEETIPFHIKNQIQHQHQEQNHPNVTNPSSSSFSSLFPSPRKASY
NPSLTLLDPINFSPALDPLNNNLTRSFYSNPAVQFKSFPDNNASNCGLALPLSSYGRSPSSITG

FNQNFPAQSIPMTPPSLHYSTSDFETNMSFTSLIMGAQVEPNELFPGLGSEIPSDQTPPRPNTP
FSSNTSGGVCVREESSKNTDNDSETVVPEMMHDNRNSGLLDALLLESQNLSRKEGKLTGE
NSLVATDQKGKRVVDESAEEEEEETEKEAA
KRVKLSAMNGSENSGENNCCDDLSSSQSSIGVKPNEEPMDEMNSMDDDLLSLLDNFPTTT
PLPEWYRSRNIASGLSSSTVVEGGEVEAEQEASLAGGETTDETPNVDWAFGSSYWNNMPG
IC
>Manes.13G072300
MADLDHSSSDDVSVDSREESSQESKLEFTEDEETLITRMYNLVGERWPLIAGRIPGRTAEEIE
KYWNSRFSSSQ
>Manes.17G035600MAESEHSSSDETYVNSQERRNLEARLEFSEEEEELVIRMFNLVGERWPL
IAGRIPGKTAEEIEKYWKSRYSTSE

Additional table 2. 166 R2R3 MYB genes expressed in both ethylene- and water-deficit stress-induced leaf abscission

gene	KEGG orthology	treatment	T1	T2	T3	T4	T5	T6
Manes.05G098700	AT2G02820.2	et	0	0.378401	0.295723	0.309293	0.330788	1.226632
Manes.05G007400	AT4G01680.1	et	0	1.044814	0.27965	0.051859	-1.04961	-1.9771
Manes.05G007400	AT4G01680.1	wd	0	0.877587	0.015069	-0.69245	-1.52699	-0.99022
Manes.01G235800	AT4G01680.1	et	0	-0.59074	-1.5454	-1.23175	-2.37782	-2.98051
Manes.08G106900	AT1G09540.1	et	0	0.534958	-0.87541	-0.74855	-3.15072	-2.39289
Manes.08G106900	AT1G09540.1	wd	0	-0.25017	-1.15104	-1.87197	-2.28279	-1.94508
Manes.02G047900	AT3G01140.1	wd	0	0.235605	-0.84089	-1.31043	-1.89057	-1.61838
Manes.01G226200	AT5G49330.1	wd	0	-1.68131	0.866156	-0.82393	-1.27928	-2.44693
Manes.08G151800	AT1G69560.1	et	0	2.484628	2.692851	2.938963	3.175397	3.567424
Manes.12G108500	AT4G37260.1	et	0	-0.53784	-0.55809	-0.80465	-1.18508	-1.33862
Manes.12G083100	AT4G38620.1	wd	0	-0.7051	-0.6783	-1.074	-1.17723	-1.94009
Manes.06G066600	AT1G68320.1	et	0	1.813607	3.888714	4.453695	5.505084	5.668091
Manes.06G066600	AT1G68320.1	wd	0	-1.26569	2.308536	3.459327	3.360954	3.677035
Manes.05G114400	AT1G68320.1	et	0	0.032524	2.284218	2.313391	3.320066	3.402067
Manes.05G114400	AT1G68320.1	wd	0	0.999278	1.915521	2.957673	2.872494	2.969215
Manes.14G077700	AT5G62470.2	et	0	0.814509	0.28404	0.397036	-0.50943	-1.06462
Manes.14G077700	AT5G62470.2	wd	0	0.339822	-0.72023	-1.72309	-1.39821	-2.24948
Manes.08G011300	AT5G57620.1	et	0	-0.70392	-1.13289	-1.07704	-1.13543	-0.56897
Manes.03G052800	AT3G13540.1	et	0	0.254715	1.440899	1.327342	2.012497	2.452912
Manes.03G052800	AT3G13540.1	wd	0	-1.15586	-1.35993	-0.67669	-1.25291	-0.24161
Manes.01G118700	AT5G16600.1	et	0	-1.92741	-3.20423	-3.87408	-3.72738	-4.64025
Manes.01G118700	AT5G16600.1	wd	0	2.581954	2.283418	1.99675	3.290115	2.217231
Manes.05G020800	AT1G22640.1	et	0	-1.74082	-1.66658	-3.4112	-1.36885	-1.45759
Manes.05G020800	AT1G22640.1	wd	0	-1.75779	-1.79336	-2.28069	-1.90509	-3.48036
Manes.01G074400	AT4G12350.1	et	0	0.245252	-2.50144	-4.66933	-4.24332	-5.8039
Manes.01G074400	AT4G12350.1	wd	0	0.309875	-0.74637	-0.9487	-1.40163	-2.76415
Manes.05G012100	AT5G49620.1	et	0	-1.02503	2.830783	2.769856	4.062286	4.049247
Manes.05G012100	AT5G49620.1	wd	0	-0.41254	2.498583	3.171911	3.046578	3.417056
Manes.16G085200	AT5G35550.1	et	0	0.313826	0.006478	-0.04186	-1.07552	-0.96438
Manes.16G085200	AT5G35550.1	wd	0	-0.28051	-0.60231	-0.57777	-0.35107	-1.90725
Manes.15G040700	AT3G23250.1	et	0	0.370388	3.316334	2.624148	2.019844	2.270021
Manes.15G040700	AT3G23250.1	wd	0	0.437015	0.306612	1.035272	-0.54835	0.405121
Manes.11G134300	AT3G46130.1	et	0	-0.16553	-0.9526	-1.46953	-2.89539	-4
Manes.11G134300	AT3G46130.1	wd	0	-0.9032	-1.35292	-2.0517	-2.94231	-2.89432
Manes.02G074300	AT4G38620.1	wd	0	-1.4135	-0.50042	-0.84606	-1.13226	-1.41465
Manes.01G115400	AT4G38620.1	wd	0	-2.52949	-1.49411	-1.95232	-2.83033	-2.95232
Manes.04G144400	AT5G14750.1	et	0	0.464041	0.492315	0.62499	-1.10998	-1.82623
Manes.04G144400	AT5G14750.1	wd	0	-0.79236	-1.58251	-2.88897	-3.59423	-2.84581
Manes.11G127600	AT1G17950.1	et	0	-0.91757	-1.82828	-2.50144	-2.10408	-2.87832

Manes.11G127600	AT1G17950.1	wd	0	-0.50512	-0.84296	-2.45878	-2.18246	-1.52367
Manes.14G104200	AT1G68320.1	et	0	1.143589	2.738746	3.645851	4.119688	4.351876
Manes.14G104200	AT1G68320.1	wd	0	-0.61198	1.265437	1.730575	1.549176	1.770322
Manes.11G020600	AT2G16720.1	et	0	-0.53659	0.170822	0.678342	1.653381	1.914985
Manes.03G117500	AT4G21440.1	et	0	2.709379	3.747226	4.096388	3.943528	3.920389
Manes.03G117500	AT4G21440.1	wd	0	-1.32085	0.715718	1.288063	1.511417	1.893595
Manes.01G231300	AT2G47190.1	et	0	#NUM!	3.559369	3.728149	6.024694	5.911814
Manes.05G177900	AT4G37260.1	et	0	0.205393	1.185486	1.056167	0.925696	0.847115
Manes.05G177900	AT4G37260.1	wd	0	0.035905	0.65205	1.441749	-0.00405	0.679244
Manes.14G034400	AT4G12350.1	wd	0	-1.40469	-0.90077	-2.72738	-4.14816	-4.12281
Manes.15G081900	AT4G21440.1	et	0	#NUM!	1.402067	1.005112	2.770744	3.310718
Manes.15G081900	AT4G21440.1	wd	0	0.97475	1.395885	2.858001	2.915636	3.117462
Manes.08G145500	AT3G13540.1	wd	0	-1.62593	-0.39916	-0.74034	-1.42701	-1.06975
Manes.03G208500	AT3G06490.1	et	0	0.460061	2.393691	2.481971	3.336812	3.544498
Manes.06G109000	AT5G26660.1	et	0	-0.56235	-1.1078	-3.30472	-3.32337	-4.45166
Manes.08G151100	AT1G08810.1	et	0	1.007985	-0.26986	-0.21709	-0.80768	-1.80591
Manes.08G151100	AT1G08810.1	wd	0	0.34426	-0.54519	-1.69385	0.340847	-0.86145
Manes.15G006600	AT2G47460.1	et	0	-0.0613	-3.18442	-2.67208	-4.08314	-4.42043
Manes.15G006600	AT2G47460.1	wd	0	0.766807	-1.09974	-0.84192	-2.21759	-1.75341
Manes.02G194800	AT1G22640.1	et	0	-0.2676	1.159177	1.148934	1.460166	1.606774
Manes.02G194800	AT1G22640.1	wd	0	0.39594	1.043624	1.656679	1.70075	1.928238
Manes.02G017300	AT1G63910.1	wd	0	-1.53324	-1.05559	-3.0315	-3.59248	-3.5649
Manes.01G057200	AT1G63910.1	et	0	-0.90293	-0.75901	-1.87938	-3.4852	-3.52118
Manes.01G057200	AT1G63910.1	wd	0	-0.75706	-1.32301	-2.9737	-2.74371	-2.90725
Manes.11G020400	AT5G14750.1	wd	0	-0.54036	-1.52077	-1.80239	-2.09357	-1.46395
Manes.02G034300	AT4G12350.1	et	0	0.02432	-2.53701	-4.27649	-1.8762	0.258459
Manes.02G034300	AT4G12350.1	wd	0	0.482848	-0.86907	-1.81502	-1.87673	-1.7471
Manes.06G136500	AT4G12350.1	et	0	0.638584	-0.75633	-0.56512	-3.12532	-4.85726
Manes.06G136500	AT4G12350.1	wd	0	-0.89943	-1.75195	-3.88257	-4.39593	-5.29336
Manes.09G135700	AT3G28910.1	et	0	0.173639	-0.90644	-1.09696	-1.18017	-1.69759
Manes.09G135700	AT3G28910.1	wd	0	0.887369	0.358846	-0.55216	1.384492	-0.19429
Manes.18G103600	AT4G09460.1	et	0	0.606821	1.480782	1.62396	2.052346	2.047818
Manes.14G061700	AT5G26660.1	et	0	0.244644	-0.38007	-1.23379	-2.04931	-3.64747
Manes.14G061700	AT5G26660.1	wd	0	0.735262	0.257976	-1.32554	-0.42721	-1.68919
Manes.15G003000	AT3G06490.1	et	0	#NUM!	1.142087	0.562865	1.231371	3.047573
Manes.14G066200	AT5G14750.1	et	0	-1.01829	-2.39593	-2.91376	-3.3555	-4.24332
Manes.14G066200	AT5G14750.1	wd	0	-1.53032	0.165944	-0.54309	-1.69806	-1.89808
Manes.18G042000	AT5G67300.1	wd	0	0.927214	0.747516	1.180784	0.161307	0.551393
Manes.S062600	AT2G31180.1	et	0	1.351515	3.179256	1.002166	0.585251	-0.57949
Manes.01G083100	AT5G14750.1	et	0	0.750349	2.637147	4.49211	0.901572	1.47254

Red font represents the R2R3 MYB genes that express in water-deficit stress induced abscission; Black font represents the R2R3 MYB genes that express in ethylene induced abscission; and yellow shadow represents the R2R3 MYB genes that express both in ethylene- and water-deficit stress-

induced abscission.

Additional table 3. SOTA clustering of R2R3 MYB genes expressed in response to water-deficit stress induced abscission.

id	T1	T2	T3	T4	T5	T6	Cluster
Manes.15G040700	0	0.437015	0.306612	1.035272	-0.54835	0.405121	WS1
Manes.05G177900	0	0.035905	0.652051	1.441749	-0.00405	0.679244	WS1
Manes.18G042000	0	0.927214	0.747516	1.180784	0.161307	0.551393	WS1
Manes.05G114400	0	0.999279	1.915521	2.957673	2.872494	2.969215	WS2
Manes.01G118700	0	2.581954	2.283418	1.99675	3.290115	2.217231	WS2
Manes.15G081900	0	0.97475	1.395885	2.858001	2.915636	3.117462	WS2
Manes.02G194800	0	0.39594	1.043625	1.656679	1.700751	1.928238	WS2
Manes.06G066600	0	-1.26569	2.308536	3.459327	3.360954	3.677035	WS3
Manes.05G012100	0	-0.41254	2.498583	3.171911	3.046578	3.417056	WS3
Manes.14G104200	0	-0.61198	1.265437	1.730575	1.549176	1.770322	WS3
Manes.03G117500	0	-1.32085	0.715718	1.288063	1.511417	1.893595	WS3
Manes.12G083100	0	-0.7051	-0.6783	-1.074	-1.17723	-1.94009	WS4
Manes.05G020800	0	-1.75779	-1.79336	-2.28069	-1.90509	-3.48036	WS4
Manes.16G085200	0	-0.28051	-0.60231	-0.57777	-0.35107	-1.90725	WS4
Manes.11G134300	0	-0.9032	-1.35292	-2.0517	-2.94231	-2.89432	WS4
Manes.14G034400	0	-1.40469	-0.90077	-2.72738	-4.14816	-4.12281	WS4
Manes.02G017300	0	-1.53324	-1.05559	-3.0315	-3.59248	-3.56491	WS4
Manes.01G226200	0	-1.68131	0.866156	-0.82393	-1.27928	-2.44693	WS5
Manes.03G052800	0	-1.15586	-1.35993	-0.67669	-1.25291	-0.24161	WS5
Manes.02G074300	0	-1.4135	-0.50042	-0.84607	-1.13226	-1.41465	WS5
Manes.01G115400	0	-2.52949	-1.49411	-1.95232	-2.83033	-2.95232	WS5
Manes.08G145500	0	-1.62593	-0.39916	-0.74034	-1.42701	-1.06975	WS5
Manes.14G066200	0	-1.53032	0.165944	-0.54309	-1.69806	-1.89808	WS5
Manes.05G007400	0	0.877587	0.015069	-0.69245	-1.52699	-0.99022	WS6
Manes.08G106900	0	-0.25017	-1.15104	-1.87197	-2.28279	-1.94508	WS6
Manes.02G047900	0	0.235605	-0.84089	-1.31043	-1.89057	-1.61838	WS6
Manes.14G077700	0	0.339822	-0.72023	-1.72309	-1.39821	-2.24948	WS6
Manes.01G074400	0	0.309875	-0.74637	-0.9487	-1.40163	-2.76415	WS6
Manes.04G144400	0	-0.79236	-1.58251	-2.88897	-3.59423	-2.84581	WS6
Manes.11G127600	0	-0.50512	-0.84296	-2.45878	-2.18246	-1.52367	WS6
Manes.08G151100	0	0.34426	-0.54519	-1.69385	0.340847	-0.86145	WS6
Manes.15G006600	0	0.766807	-1.09974	-0.84192	-2.21759	-1.75341	WS6
Manes.01G057200	0	-0.75706	-1.32301	-2.9737	-2.74371	-2.90725	WS6
Manes.11G020400	0	-0.54036	-1.52077	-1.80239	-2.09357	-1.46395	WS6
Manes.02G034300	0	0.482848	-0.86907	-1.81502	-1.87673	-1.7471	WS6
Manes.06G136500	0	-0.89943	-1.75195	-3.88257	-4.39593	-5.29336	WS6
Manes.09G135700	0	0.887369	0.358846	-0.55216	1.384492	-0.19429	WS6
Manes.14G061700	0	0.735262	0.257976	-1.32554	-0.42721	-1.68919	WS6

Additional table 4. SOTA clustering of R2R3 MYB genes expressed in response to ethylene induced abscission.

id	T1	T2	T3	T4	T5	T6	Cluster
Manes.05G098700	0	0.378401	0.295723	0.309293	0.330788	1.226632	ES1
Manes.05G114400	0	0.032524	2.284218	2.313391	3.320066	3.402067	ES1
Manes.03G052800	0	0.254715	1.440899	1.327343	2.012497	2.452912	ES1
Manes.05G012100	0	-1.02503	2.830783	2.769857	4.062286	4.049247	ES1
Manes.11G020600	0	-0.53659	0.170822	0.678342	1.653381	1.914985	ES1
Manes.01G231300	0	0	3.559369	3.728149	6.024694	5.911814	ES1
Manes.15G081900	0	0	1.402067	1.005113	2.770745	3.310718	ES1
Manes.03G208500	0	0.460061	2.393691	2.481971	3.336812	3.544498	ES1
Manes.02G194800	0	-0.2676	1.159177	1.148934	1.460166	1.606774	ES1
Manes.15G003000	0	0	1.142087	0.562865	1.231371	3.047573	ES1
Manes.08G151800	0	2.484628	2.692851	2.938963	3.175397	3.567424	ES2
Manes.06G066600	0	1.813607	3.888714	4.453695	5.505084	5.668091	ES2
Manes.15G040700	0	0.370388	3.316334	2.624148	2.019844	2.270021	ES2
Manes.14G104200	0	1.143589	2.738746	3.645852	4.119688	4.351876	ES2
Manes.03G117500	0	2.709379	3.747226	4.096388	3.943528	3.920389	ES2
Manes.05G177900	0	0.205393	1.185486	1.056167	0.925696	0.847115	ES2
Manes.18G103600	0	0.606821	1.480782	1.623961	2.052346	2.047818	ES2
Manes.01G083100	0	0.750349	2.637147	4.49211	0.901572	1.47254	ES2
Manes.05G020800	0	-1.74082	-1.66658	-3.4112	-1.36885	-1.45759	ES3
Manes.14G066200	0	-1.01829	-2.39593	-2.91376	-3.3555	-4.24332	ES3
Manes.08G011300	0	-0.70392	-1.13289	-1.07704	-1.13543	-0.56897	ES4
Manes.02G034300	0	0.02432	-2.53701	-4.27649	-1.8762	0.258459	ES4
Manes.12G108500	0	-0.53784	-0.55809	-0.80465	-1.18508	-1.33862	ES5
Manes.01G118700	0	-1.92741	-3.20423	-3.87408	-3.72738	-4.64025	ES5
Manes.01G074400	0	0.245252	-2.50144	-4.66933	-4.24332	-5.8039	ES5
Manes.11G127600	0	-0.91757	-1.82828	-2.50144	-2.10408	-2.87832	ES5
Manes.06G109000	0	-0.56235	-1.1078	-3.30472	-3.32337	-4.45166	ES5
Manes.15G006600	0	-0.0613	-3.18442	-2.67208	-4.08314	-4.42043	ES5
Manes.09G135700	0	0.173639	-0.90644	-1.09696	-1.18017	-1.69759	ES5
Manes.05G007400	0	1.044814	0.27965	0.051859	-1.04961	-1.9771	ES6
Manes.01G235800	0	-0.59074	-1.5454	-1.23175	-2.37782	-2.98051	ES6
Manes.08G106900	0	0.534958	-0.87541	-0.74855	-3.15072	-2.39289	ES6
Manes.14G077700	0	0.814509	0.28404	0.397036	-0.50943	-1.06462	ES6
Manes.16G085200	0	0.313826	0.006478	-0.04186	-1.07552	-0.96438	ES6
Manes.11G134300	0	-0.16553	-0.9526	-1.46953	-2.8954	-4	ES6
Manes.04G144400	0	0.464041	0.492315	0.62499	-1.10998	-1.82623	ES6
Manes.08G151100	0	1.007985	-0.26986	-0.21709	-0.80768	-1.80591	ES6
Manes.01G057200	0	-0.90293	-0.75901	-1.87938	-3.4852	-3.52118	ES6
Manes.06G136500	0	0.638584	-0.75633	-0.56512	-3.12532	-4.85726	ES6
Manes.14G061700	0	0.244644	-0.38007	-1.23379	-2.04931	-3.64747	ES6

Manes.S062600	0	1.351515	3.179256	1.002166	0.585251	-0.57949	ES6

Additional table 5. Summary of abiotic stress-inducible cis-elements in cassava ERF subfamily transcription factor promoter regions.

**Abiotic stress**	**Gene name** / ***cis*-element**	**motif sequence**	**Manes.15 G040700**	**Manes.05 G177900**	**Manes.05 G114400**	**Manes.01 G118700**	**Manes.15 G081900**
Drought -stress	S000133	CCACG TGG	0	0	0	0	0
	S000153	CCGAC	0	4	0	2	0
	S000174	CACATG	6	0	2	0	4
	S000175	CTAACC A	0	0	0	0	0
	S000176	CNGTTR	2	24	4	0	8
	S000177	TAACTG	0	4	0	0	2
	S000402	ACCGA C	0	0	0	2	0
	S000408	WAACC A	6	2	8	2	6
	S000413	CATGTG	6	0	2	0	4
	S000414	ACGTG	4	10	0	8	10
	S000415	ACGT	16	24	4	16	32
	S000418	RCCGA C	0	2	0	0	0
	total		**40**	60	20	30	66
Wound-stress	S000244	AACGT GT	0	0	0	0	0
	S000444	AGATCC AA	0	0	0	0	0
	S000457	TGACY	8	12	10	8	22
	S000037	AWTTC AAA	2	2	0	2	8
	total		**10**	14	10	10	30

**Abiotic stress**	**Gene name** / ***cis*-element**	**motif sequence**	**Manes.02 G194800**	**Manes.06 G066600**	**Manes.05 G012100**	**Manes.14 G104200**	**Manes.03 G117500**
Drought-stress	S000133	CCACGTGG	0	0	0	0	0
	S000153	CCGAC	0	2	2	0	4
	S000174	CACATG	0	2	2	4	4
	S000175	CTAACCA	0	0	0	0	0
	S000176	CNGTTR	16	10	4	2	2
	S000177	TAACTG	6	0	0	0	0
	S000402	ACCGAC	0	0	0	0	2
	S000408	WAACCA	10	0	2	2	2
	S000413	CATGTG	0	2	2	4	4
	S000414	ACGTG	4	2	2	4	8
	S000415	ACGT	8	4	12	24	20
	S000418	RCCGAC	0	0	0	0	2
	total		44	**22**	**26**	**40**	**48**
Wound-stress	S000244	AACGTGT	0	0	2	0	0
	S000444	AGATCCAA	0	0	0	0	0
	S000457	TGACY	8	6	14	8	16
	S000037	AWTTCAAA	0	2	4	2	0
	total		**8**	**8**	18	**10**	**16**

Additional table 6. Forward and reverse primers used for qRT-PCR analysis of R2R3 MYB gene expression.

Gene ID	Primer Designation	Sequences
Manes.15G040700	Forward	TGCTGGGAAATAGTGGTCAGC
	Reverse	TGCAAGATTCCCCTGGATGT
Manes.05G177900	Forward	CTTGAGCCTATCTCTTCCTG
	Reverse	CTGTTTCTCTACTCGATTCCAC
Manes.05G114400	Forward	CTCAAGTATCAGAATACCCAGC
	Reverse	CTTCAGATACTTAACCACTGAGT
Manes.01G118700	Forward	CTTGGAAACAGATGGTCCAGG
	Reverse	CTGAAGCAACTAACAAACAAGC
Manes.15G081900	Forward	GATGAGTAACCAAGCCATGATG
	Reverse	GATGATTGAGAAGAGACGAACG
Manes.02G194800	Forward	CGATAGGGCTACCTCCAATGC
	Reverse	CATTCAATTGCTCGAATCTAACC
Manes.06G066600	Forward	CACCTCTTCTCGATAGTACAG
	Reverse	GGACCCTAATCATCAGATCCAC
Manes.05G012100	Forward	GGCAATAGGTGGTCGAAAATAG
	Reverse	GTTCACACACTTGATGAGTCA
Manes.14G104200	Forward	TCCACCAGTGCTTGATAACAC
	Reverse	CTAACCATAATCACAGAGCCA
Manes.03G117500	Forward	AGAGCTTGACGCCATACTGTG
	Reverse	CGTAAGCTGCTTACTAGATTATC

Additional table 7. Forward and reverse primers used for qRT-PCR analysis of *CAT*s and *KNOX*s acted downstream of selected *MYB*s.

Gene ID	Primer Designation	Sequences
Manes.05G130700 (*CAT1*)	Forward	CTGGACACAGTGTGACCGAT
	Reverse	GATTCTGAACCAGTATCCTCG
Manes.05G130500 (*CAT2*)	Forward	TACTGGTCTCAGTGCGACAA
	Reverse	CTTCCTGAGAACTCGCCACC
Manes.14G138700 (*KNOX1*)	Forward	GGTGGACGAGAAAGAGCTTG
	Reverse	TCAGAGAGTGACACACAACG
Manes.06G106700 (*KNOX2*)	Forward	ATGCAGAGAGGTGGTGGTGC
	Reverse	CCTTCTGGCAATCAATATAAGC
